# Relative and Absolute Stereochemistry of Diacarperoxides: Antimalarial Norditerpene Endoperoxides from Marine Sponge *Diacarnus megaspinorhabdosa*

**DOI:** 10.3390/md12084399

**Published:** 2014-08-08

**Authors:** Fan Yang, Yike Zou, Ru-Ping Wang, Mark T. Hamann, Hong-Jun Zhang, Wei-Hua Jiao, Bing-Nan Han, Shao-Jiang Song, Hou-Wen Lin

**Affiliations:** 1 Key Laboratory for Marine Drugs, Department of Pharmacy, Renji Hospital, Shanghai Jiao Tong University School of Medicine, Shanghai 200127, China; E-Mails: bill1985@126.com (F.Y.); wangruping_sy@163.com (R.-P.W.); jiaoweihua1982@sina.com (W.-H.J.); hanbingnan@shsmu.edu.cn (B.-N.H.); 2 Department of Pharmacognosy and National Center for Natural Products Research (NCNPR), School of Pharmacy, The University of Mississippi, Oxford, MS 38677, USA; E-Mails: yzou@olemiss.edu (Y.Z.); mthamann@olemiss.edu (M.T.H.); 3 School of Traditional Chinese Materia Medica, Shenyang Pharmaceutical University, Shenyang 110016, China; 4 Dujiangyan Center of Aeromedical Assessment and Training of Air Force, Dujiangyan 611830, China; E-Mail: chinabludo@126.com; 5 Laboratory of Marine Drugs, Department of Pharmacy, Changzheng Hospital, Second Military Medical University, Shanghai 200003, China

**Keywords:** antimalarial, marine sponge, endoperoxide

## Abstract

Five new norditerpene endoperoxides, named diacarperoxides H–L (**1**–**5**), and a new norditerpene diol, called diacardiol B (**6**), were isolated from the South China Sea sponge, *Diacarnus megaspinorhabdosa*. Their structures, including conformations and absolute configurations, were determined by using spectroscopic analyses, computational approaches and chemical degradation. Diacarperoxides H–J (**1**–**3**) showed some interesting stereochemical issues, as well as antimalarial activity.

## 1. Introduction

**Figure 1:**
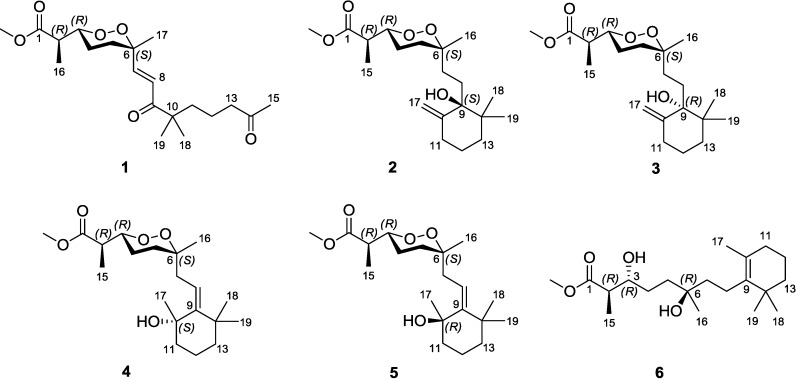
Chemical structures of Compounds **1**–**6**.

Malaria kills a child every minute, 154–289 million people are infected each year [1]. The plant-derived quinine family of antimalarials has provided centuries of relief for this disease. The long history of usage had, however, produced drug resistance, which was then relieved by incorporating the artemisinin class isolated from the plant *Artemisia annua* [2,3]. Exploring bioactive secondary metabolites from marine invertebrates has provided us potent leads fighting against this disease [4], the marine-derived endoperoxide family has been known for decades and has generated interest for drug discovery [5,6]. Computation is now seeing greater utility in the natural product field, including quantitative modeling on the molecular scale to disclose structural information, as well as in complex molecular systems to rationalize bioactivities targeting fatal diseases [7,8,9]. By using computational approaches, spectroscopic analyses and chemical degradation, we have assigned five new norditerpene endoperoxides, named diacarperoxides H–L (**1**–**5**), together with a new norditerpene diol, diacardiol B (**6**) from the marine sponge, *Diacarnus megaspinorhabdosa* (Figure 1). Herein, we report the details of the isolation and structural elucidation of these compounds.

## 2. Results and Discussion

Five new norditerpene endoperoxides (**1**–**5**) were obtained from the CH_2_Cl_2_ extract of the sponge. The signal patterns for a 1,2-dioxane ring and a substituted methyl propionate group were acquired from NMR, establishing a scaffold of the marine-derived endoperoxide class [10,11]. Further analysis of the NMR spectra (Table 1 and Table 2) provided evidence for the existence of monoterpene substitutions different from reported derivatives [12,13]. The stereochemical issues were encountered either because of conformational flexibility or two distal stereogenic centers (*vide infra*). Traditionally, the determination of the relative configurations of this type of compound counted on the chemical shift differences based on the empirical rule developed by Capon and Macleod [14], and the absolute configuration primarily relied on optical rotation [15] or conducting semisynthesis, since only three XRD (X-ray diffraction) structures in this class were reported [6,16,17]. In this work, the structures of the new norditerpene endoperoxides were determined by using spectroscopic analyses and computational approaches, including DFT (density functional theory)-based molecular modeling, conformational analysis and NMR and ECD (electronic circular dichroism) calculations.

**Figure 2:**
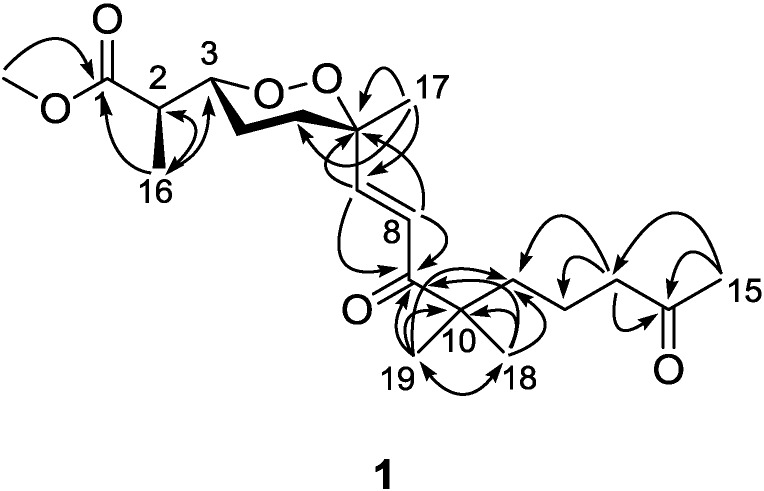
Key HMBC correlations for Compound **1**.

**Figure 3:**
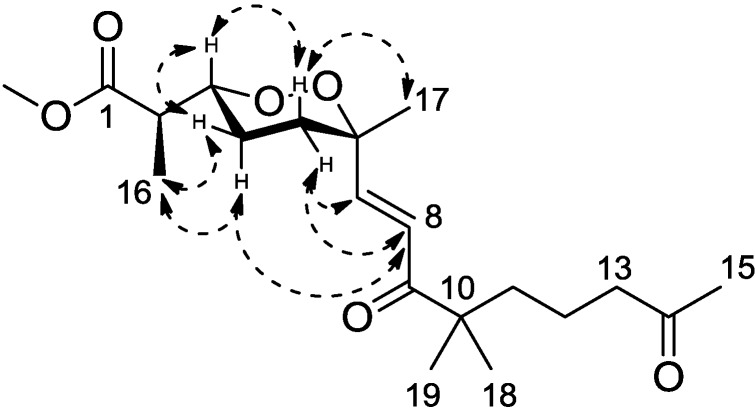
Key NOESY correlations for Compound **1**.

**Figure 4:**
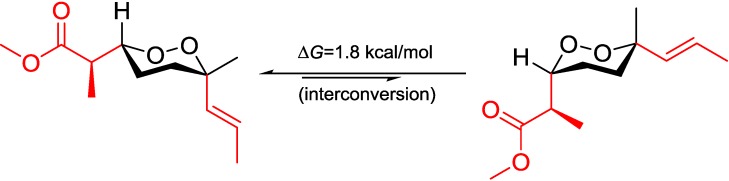
*A*-value determination of the endoperoxide core.

Diacarperoxide H (**1**) was isolated as colorless oil. The molecular formula of C_20_H_32_O_6_ was determined by the analysis of HRESIMS (high resolution electrospray ionization mass spectroscopy) data with five degrees of unsaturation. The ^1^H and ^13^C NMR signal patterns suggested a norditerpene endoperoxide core and established an isolated spin system, allowing the assignment of C-1–C-6, C-16 and C-17 positions [18,19]. Analyzing from the remaining signals in the low field region of ^13^C NMR, another two carbonyl functional groups (δ_C_ 208.5 and 203.8) and two olefinic carbons (δ_C_ 147.6 and 124.3) were deduced. The sp^2^ carbons (δ_C_ 124.3 and 147.6) were proven to be located at the α and β positions of the carbonyl (δ_C_ 203.8), based on the HMBC correlations from the corresponding olefinic protons H-7 (δ_H_ 6.88) and H-8 (δ_H_ 6.75) to the carbonyl C-9 (δ_C_ 203.8) (Figure 2). This olefinic functional group was attached to the quaternary C-6 (δ_C_ 80.9) on the endoperoxide core, which was deduced from the HMBC correlations from H-7 (δ_H_ 6.88) and H-8 (δ_H_ 6.75) to C-6, thus assigning the positions of C-7 and C-8, respectively. The large coupling constant of the two olefinic protons (15.6 Hz) and the NOESY correlation between H-8 (δ_H_ 6.75) and 17-methyl protons (δ_H_ 1.24) determined a *trans* double bond in an isolated spin system. Another quaternary carbon (C-10, δ_C_ 46.5) with geminal dimethyl groups was connected to the carbonyl C-9 according to the HMBC correlations from six overlapped methyl protons (δ_H_ 1.14) to C-9. The remaining one covalent connection of C-10 elongated an unbranched alkyl chain through three consecutive methylene groups to a terminal acetyl functional group, establishing the last spin system, on the basis of COSY correlations of 11-CH_2_ and 12-CH_2_ (δ_H_ 1.51) with 13-CH_2_ (δ_H_ 2.41). The HMBC correlations from dimethyl protons 18-CH_3_ and 19-CH_3_ (δ_H_ 1.14) to the methylene carbon C-11 (δ_C_ 38.8) and from the methylene protons 13-CH_2_ and methyl protons 15-CH_3_ (δ_H_ 2.12) to the carbonyl C-14 (δ_C_ 208.5), aligning the positions C-11–C-15. Attaching to the endoperoxide core, the acyclic side chain (C-7–C-15) was elucidated furnishing the gross structure of **1**. The signal splitting pattern of H-3 (δ_H_ 4.32, *J* = 9.0, 2.5 Hz) suggested *anti* and *gauche* relationships between H-3-H-4_ax_ and H-3-H-4_eq_, respectively. According to the Karplus equation [20], H-3 was therefore in the axial position representing major conformations. These spatial relationships were then confirmed by the strong NOESY correlations of H-3 (δ_H_ 4.32)/H-4b (δ_H_ 1.68) and H-5a (δ_H_ 1.83). On the other side, the olefinic C-7 was in the axial position based on the NOESY correlation between H-7 (δ_H_ 6.88) and H-5b (δ_H_ 2.03), H-5a (δ_H_ 1.83) and Me-17 (δ_H_ 1.24), H-5b and H-8 (δ_H_ 6.75), H-4a (δ_H_ 1.51) and H-8 (Figure 3). Calculations were then conducted to confirm this conformation. Resulting from conformational analyses and DFT calculations, an averaged *A*-value of 1.8 kcal/mol was attributed to **1** from our model study (Figure 4) [21].

**Table 1:** ^1^H NMR data of Compounds **1**–**6** in CDCl_3_ (500 MHz).

Position	1, δ_H_, mult. (*J* in Hz)	2, δ_H_, mult. (*J* in Hz)	3, δ_H_, mult. (*J* in Hz)	4, δ_H_, mult. (*J* in Hz)	5, δ_H_, mult. (*J* in Hz)	6, δ_H_, mult. (*J* in Hz)
2	2.41, t (7.2)	2.54, quin (6.5)	2.60, quin (7.5)	2.59, quin (7.5)	2.65, quin (7.5)	2.56, quin (7.0)
3	4.32, td (9.0, 2.5)	4.23, td (9.0, 2.5)	4.24, td (9.0, 1.5)	4.23, ddd (8.0, 6.5, 2.0)	4.23, ddd (8.0, 6.5, 1.5)	3.70, m
4	H_4a(ax)_, 1.51, m	1.67, m	1.64, m	1.72, m	1.72, m	H_4a_, 1.56, m
	H_4b(eq)_, 1.68, m					H_4b_, 1.69, m
5	H_5a(ax)_, 1.83, td (13.2, 4.5)	1.67, m	1.64, m	H_5a(ax)_, 1.72, m	1.72, m	H_5a_, 1.59, m
	H_5b(eq)_, 2.03, dt (13.5, 3.6)			H_5b(eq)_, 1.83, m	1.83, m	H_5b_, 1.69, m
7	6.88, d (15.6)	H_7a_, 1.30, td (8.0, 4.5)	H_7a_, 1.18, m	H_7a_, 2.42, dd (15.5, 8.0)	H_7a_, 2.83, dd (15.5, 7.0)	1.56, m
		H_7b_, 1.67, m	H_7b_, 1.90, m	H_7b_, 3.36, dd (16.0, 7.0)	H_7b_, 2.97, dd (15.0, 7.5)	
8	6.75, d (15.6)	H_8a_, 1.51, m	H_8a_, 1.64, m	5.54, t (7.5)	5.59, t (7.5)	2.03, dd (11.0, 5.0)
		H_8b_, 1.87, td (13.0, 3.0)	H_8b_, 1.90, m			
11	1.51, m	H_11a(ax)_, 2.11, td (13.0, 3.0)	H_11a(ax)_, 1.77, td (13.5, 5.5)	H_11a(ax)_,1.56, m	H_11a(ax)_,1.56, m	1.90, t (6.0)
		H_11b(eq)_, 2.27, br.d (13.0)	H_11b(eq)_, 2.30, br.d (13.5)	H_11b(eq)_,1.91, m	H_11b(eq)_,1.83, m	
12	1.51, m	1.51, m	1.64, m	1.56, m	1.56, m	H_12a_, 1.41, m
						H_12b_, 1.56, m
13	2.41, t (7.2)	H_13a(ax)_, 1.36, m	H_13a(ax)_, 1.38, br.d (13.0)	1.41, m	1.41, m	1.41, m
		H_13b(eq)_, 1.67, m	H_13b(eq)_, 1.64, m			
15	2.12, s	1.12, d (7.0)	1.14, d (7.0)	1.12, d (7.0)	1.14, d (7.0)	1.21, d (7.2)
16	1.10, d (7.2)	1.09, s	1.09, s	1.13, s	1.13, s	1.21, s
17	1.24, s	H_17a_, 4.81, br.s	H_17a_, 4.89, br.s	1.42, s	1.41, s	1.59, s
		H_17b_, 4.89, br.s	H_17b_, 4.91, br.s			
18	1.14, s	0.97, s	0.98, s	1.18, s	1.17, s	0.99, s
19	1.14, s	0.89, s	0.91, s	1.07, s	1.08, s	0.99, s
1-OCH_3_	3.70, s	3.69, s	3.70, s	3.70, s	3.70, s	3.71, s

**Table 2:** ^13^C NMR data and DEPT analysis of Compounds **1**–**6** in CDCl_3_ (125 MHz).

Position	1, δ_C_, DEPT	2, δ_C_, DEPT	3, δ_C_, DEPT	4, δ_C_, DEPT	5, δ_C_, DEPT	6, δ_C_, DEPT
1	174.1, C	174.3, C	174.3, C	174.6, C	174.6, C	176.4, C
2	42.5, CH	42.6, CH	42.5, CH	42.5, CH	42.5, CH	45.4, CH
3	81.6, CH	81.0, CH	81.2, CH	81.6, CH	81.4, CH	73.7, CH
4	23.5, CH_2_	22.4, CH_2_	22.4, CH_2_	22.7, CH_2_	22.5, CH_2_	28.8, CH_2_
5	33.5, CH_2_	33.3, CH_2_	32.9, CH_2_	32.3, CH_2_	32.4, CH_2_	37.3, CH_2_
6	80.9, C	79.9, C	79.9, C	80.2, C	80.4, C	72.7, C
7	147.6, CH	28.3, CH_2_	28.4, CH_2_	37.5, CH_2_	37.5, CH_2_	42.2, CH_2_
8	124.3, CH	25.6, CH_2_	26.1, CH_2_	120.1, CH	120.6, CH	22.8, CH_2_
9	203.8, C	80.0, C	79.6, C	152.6, C	151.6, C	136.7, C
10	46.5, C	151.3, C	150.3, C	73.9, C	74.0, C	127.0, C
11	38.8, CH_2_	33.8, CH_2_	34.0, CH_2_	44.2, CH_2_	43.9, CH_2_	32.8, CH_2_
12	19.0, CH_2_	22.8, CH_2_	22.8, CH_2_	19.4, CH_2_	19.3, CH_2_	19.5, CH_2_
13	43.9, CH_2_	38.0, CH_2_	38.1, CH_2_	39.9, CH_2_	39.9, CH_2_	39.9, CH_2_
14	208.5, C	39.9, C	39.8, C	37.5, C	37.5, C	35.1, C
15	29.8, CH_3_	12.5, CH_3_	12.8, CH_3_	13.0, CH_3_	13.0, CH_3_	14.2, CH_3_
16	12.6, CH_3_	23.9, CH_3_	23.9, CH_3_	23.8, CH_3_	24.2, CH_3_	26.6, CH_3_
17	26.1, CH_3_	107.9,CH_2_	108.6, CH_2_	29.2, CH_3_	29.4, CH_3_	19.8, CH_3_
18	24.1, CH_3_	24.1, CH_3_	24.2, CH_3_	32.3, CH_3_	32.4, CH_3_	28.7, CH_3_
19	23.9, CH_3_	22.2, CH_3_	22.4, CH_3_	32.0, CH_3_	32.0, CH_3_	28.7, CH_3_
20	51.9, OCH_3_	51.8, OCH_3_	51.9, OCH_3_	51.9, OCH_3_	51.9, OCH_3_	51.7, OCH_3_

**Figure 5:**
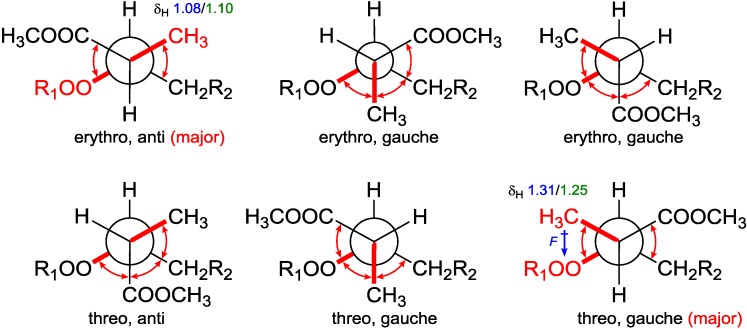
Conformational analysis of C-2–C-3 using Newman projection (blue and green numeric numbers indicating calculated and experimental values, respectively; curved red arrows indicating gauche interactions).

From the assigned conformation, the relative configuration of C-2/C-3 was then suggested as *erythro* (2*R*,3*R* or 2*S*,3*S*) by the empirical method, which was based on the ^1^H NMR chemical shift difference of the methyl group C-16 between the *erythro* and *threo* configuration [14]. To backup this method, NMR calculations were performed at different theoretical levels [22]. The calculated chemical shift for H-16 (δ_calc._ 1.08/δ_exp._ 1.10) at the B3LYP/6-31G(d,p) level matched precisely with our experimental value; while for the supposed *threo* configuration (2*R*,3*S* or 2*S*,3*R*), the calculated value for H-16 (δ_calc. epimer_ 1.31/δ_exp._ 1.24–1.26) showed further downfield from Capon’s report [14]. These results confirmed the *erythro* configuration of C-2 with C-3 and secured Capon’s method. Not being mentioned by the report, probably because of the trivial differences, C-16 chemical shifts for those *threo* configurations were slightly upfield compared with the *erythro* ones. DFT calculations were able to track this trend and resolve the small differences, although the relative chemical shifts for ^13^C NMR could not be predicted as accurately as for the proton NMR, resulting in C-16 (δ_C_ 12.4–12.8/δ_calc._ 15.7–16.1 for *erythro*, and δ_C_ 13.2–13.5/δ_calc._ 17.0–18.1 for *threo*) [14]. NMR calculations for **2**–**5** (*vide infra*) were also conducted and matched well with the experiments (Table 3). The chemical shift differences between the two possible diastereomers revealed that C-16 was anti to the peroxide group in the *erythro* configuration, whereas it was gauche in the *threo* configuration representing the major conformations. These preferred arrangements were due to minimizing the unfavored gauche interactions (Figure 5). Calculations for the minor conformations of *erythro*/gauche and *threo*/anti showed mismatching (Table 3).

**Table 3:** Calculated NMR chemical shifts for the predominant conformations of Compounds **1**–**5** using different methods applying the PCM (polarizable continuum model) solvation model.

Species	B3LYP	MPW1PW91	Experimental	B3LYP	MPW1PW91	Experimental
	(*erythro*/*threo*)	(*erythro*/*threo*)		(*erythro*/*threo*)	(*erythro*/*threo*)	
**1**	δ_H16_ 1.08/1.31	δ_H16_ 1.01/1.25	δ_H16_ 1.10	δ_C16_ 15.8/17.5	δ_C16_ 15.0/16.7	δ_C16_ 12.6
**2**	δ_H15_ 1.11/1.29	δ_H15_ 1.03/1.24	δ_H15_ 1.09	δ_C15_ 16.1/17.0	δ_C15_ 15.0/16.2	δ_C15_ 12.5
**3**	δ_H15_ 1.13/1.35	δ_H15_ 1.06/1.29	δ_H15_ 1.09	δ_C15_ 15.9/17.9	δ_C15_ 15.0/17.0	δ_C15_ 12.8
**4**	δ_H15_ 1.11/1.34	δ_H15_ 1.04/1.26	δ_H15_ 1.13	δ_C15_ 15.7/17.9	δ_C15_ 15.0/16.6	δ_C15_ 13.0
**5**	δ_H15_ 1.12/1.36	δ_H15_ 1.05/1.30	δ_H15_ 1.13	δ_C15_ 16.1/18.1	δ_C15_ 15.3/17.2	δ_C15_ 13.0

Calculated chemical shifts (in ppm) of C-16 and H-16 for **1**, and C-15 and H-15 of the same position for **2**–**5**, basis set of 6-31G(d,p).

On the grounds of these results, we believed that it is the field effect produced by the peroxide moiety withdrawing electrons from the methyl C-16 in the *threo* configuration, thus deshield it to low field, whereas C-16 in the *erythro* configuration is distal from the peroxy; thus, no deshielding was observed (Figure 5). Although no XRD structure of **1** could be obtained, our calculations showed the same conformation (Figure S52 in Supplementary Information) of the endoperoxide core as all of the three closely-related crystal structures [12,13,14]. Back to our calculations, it is the configurationally dependent conformations that thus dictate the chemical shift differences and underlie the empirical rule.

**Figure 6:**
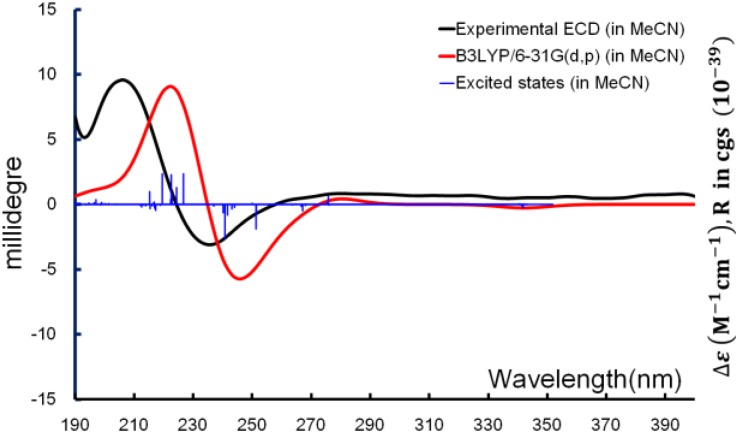
Boltzmann averaged ECD of diacarperoxide H (**1**).

With the relative configuration and major conformations of **1** established, ECD computation was used for determining the absolute configuration. Time-dependent density function theory (TDDFT) was applied for the excited-state calculations, and the experimental ECD spectrum was used for comparison. The exciton from π→π* transition of the α,β-unsaturated carbonyl producing the CE (Cotton effect) around 240 nm was able to be reproduced *in silico*. From the overlaid spectra (Figure 6), the absolute configuration of **1** was thus elucidated as 2*R*,3*R*,6*S*.

Diacarperoxide I (**2**) was obtained as a colorless oil with a molecular formula of C_20_H_34_O_5_ deduced from the HRESIMS data, implying four degrees of unsaturation. Three degrees were assigned to a carbonyl group (δ_C_ 174.3), a pair of olefinic carbons (δ_C_ 151.3, 107.9) and a typical endoperoxide ring characterized by two carbons (δ_C_ 81.0/δ_H_ 4.23, δ_C_ 79.9), while the remaining one degree of unsaturation suggested an additional ring closure. It was demonstrated containing the endoperoxide core as in **1** by comparing their 1D and 2D NMR data. The attachment on C-6 was, however, significantly different from **1**; instead of an olefinic substitution, a group of two consecutive methylene was attached to C-6, supported by the HMBC correlations of H-16 (δ_H_ 1.09)/C-7 (δ_C_ 28.3) and H-7 (δ_H_ 1.30)/C-16 (δ_C_ 23.9), in addition to the COSY correlations of H-7a (δ_H_ 1.30) with CH_2_-8 (δ_H_ 1.51, 1.87), together assigning the methylene C-7 (δ_C_ 28.3) and C-8 (δ_C_ 25.6). Another three methylenes, a dimethylated quaternary carbon, a disubstituted olefinic carbon and an oxyl quaternary carbon lynchpin tethered a six-membered ring. This arrangement was elucidated starting from the strong HMBC cross-peaks from two methyl protons 18-CH_3_ (δ_H_ 0.97, s) and 19-CH_3_ (δ_H_ 0.89, s) to the quaternary C-14 (δ_C_ 39.9) to the methyl carbon of each other and to the quaternary carbon C-9 (δ_C_ 80.0); and then, verified by the HMBC correlations from the axial H-13a (δ_H_ 1.36) to the geminal dimethyl C-18 (δ_C_ 24.1) and C-19 (δ_C_ 22.2) and from the terminal olefinic CH_2_-17 (δ_H_ 4.81, 4.89) to C-9 (δ_H_ 80.0) and C-11 (δ_H_ 33.8); and finally, confirmed by the COSY correlation of a well resolved proton resonance H-11b (δ_H_ 2.27) with CH_2_-12 (δ_H_ 1.51), fixing the positions of C-9–C-19. This enclosure together with C-8 formed a monoterpene motif attaching to the endoperoxide core.

**Figure 7:**
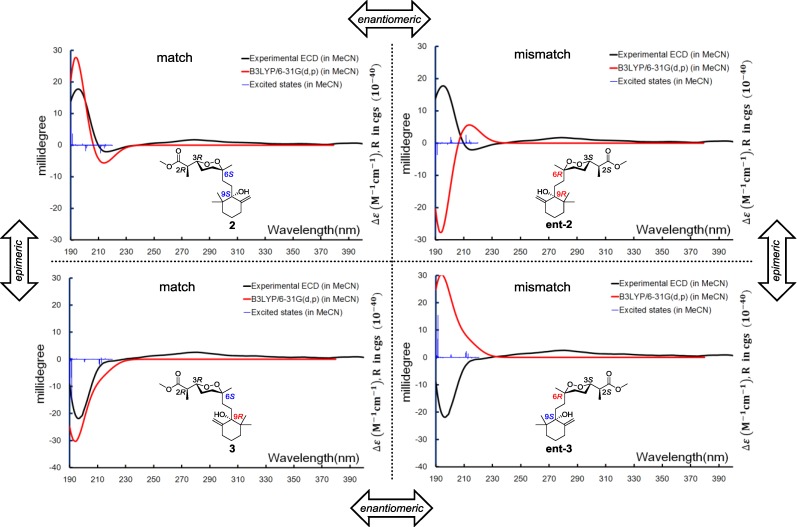
Boltzmann averaged ECD of diacarperoxides I and J (**2** and **3**).

Relative configurations for the three stereogenic centers in the endoperoxide core were determined using the same method as for **1**, but the existence of an additional center, C-9, with a tertiary hydroxyl group attachment challenged us in two different manners. First, the configuration of C-9 was difficult to be spatially related to the other three centers because of the remote distance. Second, it was difficult to handle with the limited amount of our sample by applying degradation or modification on this chemical environment. It was fortunate that a closely related compound, diacarperoxide J (**3**), was purified in our hands with the gross structure elucidated by comparing the spectroscopic data with **2**. Notably, all of the ^1^H and ^13^C NMR chemical shifts of **3** were almost identical to **2**, except for the nuclei surround C-9 (Table 1 and Table 2), which suggested a different relative configuration, but we still cannot conclude that **3** was the C-9 epimer of **2**, since the opposite stereogenicity of each center on the marine-derived endoperoxide class has been reported before [6,23,24]. Theoretically, there were four possibilities regarding the absolute configurations, e.g., for the diastereomeric-pair Compounds **2** and **3**. However, based on the fact that Compounds **2** and **3** were diastereomers other than enantiomers, there were only two possible combinations, which were 2*R*, 3*R*, 6*S*, 9*S*/2*R*, 3*R*, 6*S*, 9*R* and 2*S*, 3*S*, 6*R*, 9*R*/2*S*, 3*S*, 6*R*, 9*S*, and the Compounds **2** and **3** have to be one of those combinations. Anisotropic CEs (200 nm, positive; 210 nm, negative) of **2** and **3** were recorded by the ECD experiments, which prompted us to use exited-state calculations investigating the absolute configurations directly before establishing the relative configurations. A combination of four possible absolute configurations of **2** and **3** were modeled, and the subsequential excited-state calculations reproduced the Cotton effect, therefore assigning the absolute configurations of **2** and **3** as 2*R*,3*R*,6*S*,9*S* and 2*R*,3*R*,6*S*,9*R*, respectively (Figure 7).

**Figure 8:**
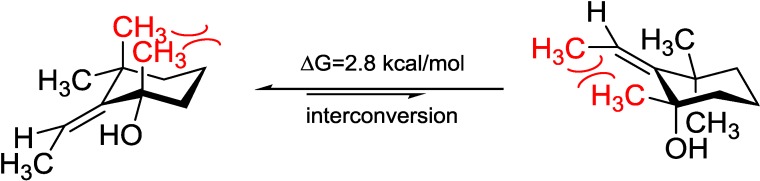
Model study of the 1,3-diaxial interaction *vs.* 1,3-allylic strain of Compounds **4** and **5** applying the solvation model.

**Figure 9:**
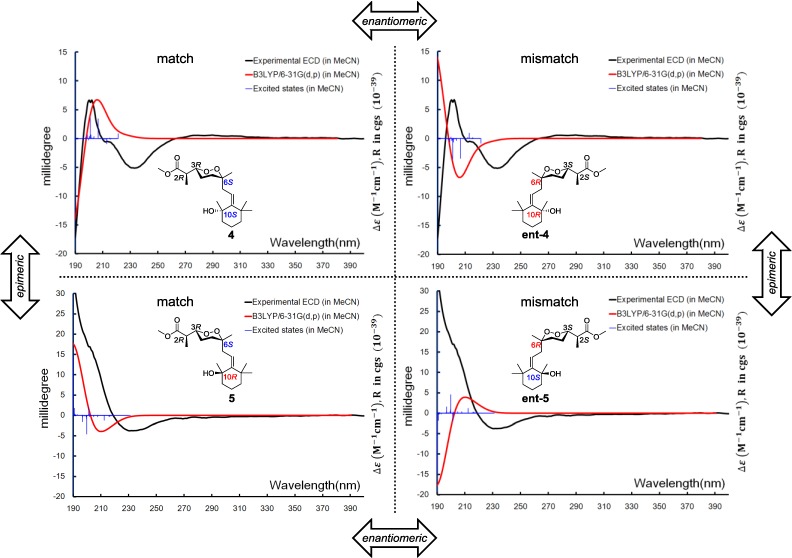
Boltzmann averaged ECD of diacarperoxides K and L (**4** and **5**).

Diacarperoxide K (**4**) was determined as a structural isomer of **2** and **3** by comparing the spectroscopic data. The differences were an extra methyl group showing 3H (δ_H_ 1.42, s) and C (δ_C_ 29.2), and a monosubstituted olefinic carbon showing C-8 (δ_C_ 120.1) and H-8 (δ_H_ 5.54, t, *J* = 7.5 Hz); in addition to the absence of C-8 methylene resonance together postulated an E1 on the tertiary hydroxyl group and a hydrolysis of the olefinic functional group of **2** or **3**. By analyzing the NMR spectra, the position of a trisubstituted olefin, assigned as C-8 and C-9, could be fixed from the COSY correlations of CH_2_-7 (δ_H_ 2.42, 3.36) with H-8 (δ_H_ 5.54). The extra tertiary methyl group showed HMBC correlations from 17-CH_3_ (δ_H_ 1.42) to C-11 (δ_C_ 44.2), C-10 (δ_C_ 73.9) and C-9 (δ_C_ 152.6), determining its position as C-17 attached to the oxyl quaternary carbon C-10 (δ_C_ 73.9), which was vicinal to the disubstituted sp^2^ C-9 (δ_C_ 152.6). The *Z* configuration of the double bond was determined by the NOESY correlations between geminal dimethyl H-18 (δ_H_ 1.18) and H-19 (δ_H_ 1.07) and the olefinic proton H-8. The remaining gross structure of **4** was unchanged from **2** and **3** by comparing the 1D and 2D NMR spectra. The same case as **2** and **3**, for the diastereomeric-pair Compounds **4** and **5**, there were only two possible combinations, which were 2*R*, 3*R*, 6*S*, 10*S*/2*R*, 3*R*, 6*S*, 10*R* and 2*S*, 3*S*, 6*R*, 10*R*/2*S*, 3*S*, 6*R*, 10*S*. The similar stereochemistry issue of C-10 was solved by comparing with the ECD spectrum of isolated epimer diacarperoxide L (**5**). Acquiring from the major conformations (Figure S52 in Supplementary Information) and the diaxial NOESY correlations of **4** and **5**, hydroxyl groups were in the equatorial positions, whereas the two methyl groups were surprisingly in the axil positions, producing inevitable 1,3-diaxial interactions, but compromising with higher *A*-values and avoiding more unfavored 1,3-allylic strains (Figure 8). Comparing the computed ECD with the experiments, the absolute configurations of **4** and **5** were thus assigned as 2*R*,3*R*,6*S*,10*S* and 2*R*,3*R*,6*S*,10*R*, respectively (Figure 9).

**Figure 10:**
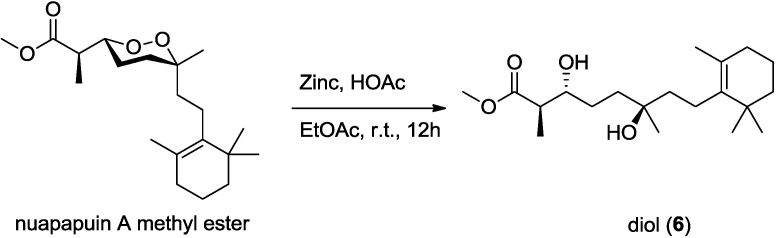
Reductive cleavage of the peroxide in nuapapuin A methyl ester.

**Figure 11:**
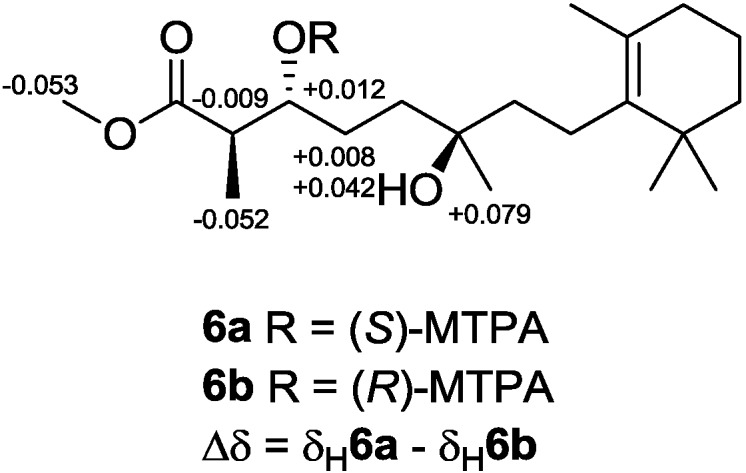
Determining the absolute configuration of diacardiol B (**6**) using the NMR anisotropy method.

Diacardiol B (**6**) was obtained as a white solid. The HRESIMS data suggested a molecular formula of C_20_H_36_O_4_ with three degrees of unsaturation. Comparing the NMR spectra with **1**–**5**, the carbon chemical shifts presumably belonging to the endoperoxide core changed significantly, in which C-3 and C-6 were upfield from 81 and 80 ppm to 73.7 and 72.7 ppm, but C-4 and C-5 were downfield from 22 and 33 ppm to 28.8 and 37.3 ppm, respectively. Compound **6** was likely generated from homolysis of the peroxy. The cyclic monoterpene part was also changed for the absence of the quaternary oxyl carbon resonance and the emergence of a tetrasubstituted olefinic C-9 (δ_C_ 136.7) and C-10 (δ_C_ 127.0). The olefin was at the C-9–C-10 position, determined by the HMBC correlations from four allylic methylene protons 8-CH_2_ (δ_H_ 2.03), 11-CH_2_ (δ_H_ 1.90) and from the vinylic methyl protons 17-CH_3_ (δ_H_ 1.59) to the olefinic carbons. Compound **6** was likely the acyclic form of nuapapuin A methyl ester. We applied chemical degradation on nuapapuin A methyl ester (which was also obtained by us from the same sponge) under reductive condition (Figure 10), and the product showed identical ^1^H NMR spectrum and similar optical rotation with **6**, verifying the structures of both of these two natural products [12,25]. To establish the absolute configuration of **6**, modified Mosher’s method [26,27] was applied, and NMR anisotropic analysis of corresponding product was used to assign the absolute configuration of **6** as 2*R*,3*R*,6*R* (Figure 11).

Diacarperoxides H–J (**1**–**3**) showed antimalarial activity against *Plasmodium falciparum* (W2 clones) *in vitro* with IC_50_ values of 12.9, 4.8 and 1.8 μM, while **2** and **3** exhibited antimalarial activity *in vitro* against *P. falciparum* (D6 clones) with IC_50_ values of 7.9 and 1.6 μM, respectively (Table 4); the IC_50_ values of the control drug artemisinin were 0.071 (D6 clones) and 0.14 μM (W2 clones). The cytotoxicity against human cancer cell lines HeLa (cervical cancer), QGY-7703 (hepatocarcinoma), MDA-MB-231 (breast adenocarcinoma) and A549 (lung carcinoma) were tested for **1**–**6** (Tables S17–S20 in Supplementary Information); none of them showed significant cytotoxicity (IC_50_ > 40 μM). Interestingly, there is no significant difference between Compounds **2** and **3** in sensitivity to the two *Plasmodium* strains above, while Compound **1** is sensitive to the W2 clone strain; this may result from the acyclic side chain (C-7–C-15). Although the security index is relatively small, this type of marine endoperoxides could still be considered as potential drug candidates acting against malaria, and structure-activity relationships (SAR) and biological studies should be encouraged to be conducted in the future.

**Table 4:** *In vitro* antimalarial activity of Compounds **1**–**3**.

Compound	*Plasmodium falciparum*			
	W2 Clone		D6 Clone	
	IC_50_ (μM)	SI ^a^	IC_50_ (μM)	SI
**1**	12.9	1.0	not active	
**2**	4.8	2.8	7.9	1.7
**3**	1.8	7.4	1.6	8.2

^a^ SI (selectivity index) = IC_50_ (Vero cells)/IC_50_ (*P. falciparum*).

## 3. Experimental Section

### 3.1. General Experimental Procedures

NMR experiments were performed on a 500 MHz instrument (Bruker Biospin Corp., Billerica, MA, USA). High-resolution mass spectroscopic data were acquired using a TOFESIMS (Waters Corp., Milford, MA, USA). ECD spectra were recorded on a CD spectropolarimeter (Jasco Inc., Tokyo, Japan) with the path length of 1 cm and a scan width of 190 to 400 nm, and optical rotation data were measured on a general polarimeter (Perkin-Elmer Inc., Waltham, MA, USA) with a sodium lamp. Reversed-phase HPLC was performed on a C_18_ column (250 × 10 mm, 5 μm) using a preparative HPLC instrument (Waters Corp., Milford, MA, USA) with a UV detector (Waters Corp., Milford, MA, USA). Column chromatography (CC) was performed on Sephadex LH-20 (Pharmacia Fine Chemicals, Piscataway, NJ, USA) and ODS-A (50 μm, YMC Co. Ltd., Kyoto, Japan) columns, and vacuum liquid chromatography (VLC) was conducted on a column packed with silica gel (200–300 mesh, Qingdao Ocean Chemical Co., Jinan, China).

### 3.2. Animal Material

The *Diacarnus megaspinorhabdosa* sample was collected from Woody Island in the South China Sea during April, 2010, and identified by Prof. Jin-He Li (Institute of Oceanology, Chinese Academy of Sciences, Qingdao, China). A voucher sample (No. XD-2010008) was deposited in the Laboratory of Marine Drugs, Department of Pharmacy, Changzheng Hospital, Second Military Medical University, China.

### 3.3. Extraction and Isolation

The sponge (2 kg, dry weight) was cut and extracted with 95% aqueous EtOH. The crude extraction was concentrated *in vacuo*, yielding a brown gum, which was then suspended in H_2_O and participated with EtOAc and *n*-BuOH to afford EtOAc and *n*-BuOH extracts, respectively. The EtOAc extract was dissolved in MeOH/H_2_O (9:1, v/v) extracted with petroleum ether to yield a brownish-red oil (30 g). The aqueous MeOH phase was then diluted to MeOH/H_2_O (3:2, v/v) and extracted with CH_2_Cl_2_ to afford a crude extract (18 g), which was then subjected to VLC packed with silica gel gradiently eluted with an *n*-hexane/acetone system to yield eight fractions (Fr. A–H). Fraction C (4.9 g) was further separated on a reversed-phase C_18_ silica gel column to obtain four subfractions (Fr. C1–C4), and subfraction C2 (210 mg) was purified by HPLC (YMC-Pack Pro C_18_ *RS*, 5 μm, 250 × 10 mm, 1.5 mL/min, UV detection at 210 and 202 nm) eluting with CH_3_OH/H_2_O (75:25) to yield **2** (16 mg) and **3** (20 mg). Similarly, subfraction C3 (150 mg) was purified by HPLC eluted with CH_3_OH/H_2_O (70:30) to yield **4** (10 mg) and **5** (7 mg). Fraction D (5.2 g) was purified by silica gel column eluted with CH_2_Cl_2_/EtOAc (8:1 to 1:1, v/v) to yield six subfractions (Fr. D1–D6), and subfraction D2 (100 mg) was isolated using a Sephadex LH-20 column eluted with CH_2_Cl_2_/MeOH (1:1, v/v) and then purified by HPLC to obtain **1** (30 mg). Fraction E (450 mg) was separated by silica gel column chromatography eluted with CH_2_Cl_2_/EtOAc (7:1–1:1, v/v) to afford **6** (18 mg).

Diacarperoxide H (**1**): colorless oil; [α]^20^_D_ +52.7 (*c* 0.28, CHCl_3_); CD (CH_3_CN, *c* 2.2 × 10^−^^4^) λ_max_ (millidegree), 192 (5.34) nm, 204 (9.43) nm, 234 (−3.05) nm; UV (CH_3_CN), λ_max_ (log ε): 196 (2.66), 224 (3.25) nm; IR (KBr) ν_max_: 3463, 2953, 1740, 1715, 1693, 1627, 1459, 1364, 1266, 1200, 1167, 1078, 1009, 982, 950, 860, 738, 477 cm^−1^; ^1^H NMR (CDCl_3_, 500 MHz) and ^13^C NMR (CDCl_3_, 125 MHz) data, see Table 1 and Table 2; HRESIMS *m*/*z* 391.2092 (calcd. for C_20_H_32_O_6_Na, 391.2091).

Diacarperoxide I (**2**): colorless oil; [α]^20^_D_ +36.5 (*c* 0.28, CHCl_3_); CD (CH_3_CN, *c* 2.2 × 10^−^^4^) λ_max_ (millidegree), 195 (17.75) nm, 215 (−2.07) nm; UV (CH_3_CN), λ_max_ (log ε): 196 (3.23), 246 (sh, 1.16) nm; IR (KBr) ν_max_: 3552, 2934, 2864, 1740, 1456, 1337, 1261, 1200, 1160, 1060, 1013, 898, 872, 802, 692, 621, 472 cm^−1^; ^1^H NMR (CDCl_3_, 500 MHz) and ^13^C NMR (CDCl_3_, 125 MHz) data, see Table 1 and Table 2; HRESIMS *m*/*z* 377.2296 (calcd. for C_20_H_34_O_5_Na, 377.2299).

Diacarperoxide J (**3**): colorless oil; [α]^20^_D_ +59.1 (*c* 0.64, CHCl_3_); CD (CH_3_CN, *c* 2.2 × 10^−^^4^) λ_max_ (millidegree), 196 (−21.89) nm; UV (CH_3_CN), λ_max_ (log ε): 204 (3.64) nm; IR (KBr) ν_max_: 3557, 2974, 2938, 2865, 1740, 1457, 1378, 1267, 1201, 1162, 1061, 1014, 901 cm^−1^; ^1^H NMR (CDCl_3_, 500 MHz) and ^13^C NMR (CDCl_3_, 125 MHz) data, see Table 1 and Table 2; HRESIMS *m*/*z* 377.2301 (calcd. for C_20_H_34_O_5_Na, 377.2299).

Diacarperoxide K (**4**): colorless oil; [α]^20^_D_ +41.0 (*c* 0.20, CHCl_3_); CD (CH_3_CN, *c* 2.2 × 10^−^^4^) λ_max_ (millidegree), 200 (6.65) nm, 232 (−5.08) nm; UV (CH_3_CN), λ_max_ (log ε): 196 (3.23), 222 (sh, 2.72) nm; IR (KBr) ν_max_: 3458, 2930, 2873, 1740, 1458, 1374, 1265, 1199, 1164, 1074, 1012, 872 cm^−1^; ^1^H NMR (CDCl_3_, 500 MHz) and ^13^C NMR (CDCl_3_, 125 MHz) data, see Table 1 and Table 2; HRESIMS *m*/*z* 377.2297 (calcd. for C_20_H_34_O_5_Na, 377.2299).

Diacarperoxide L (**5**): colorless oil; [α]^20^_D_ +23.6 (*c* 0.38, CHCl_3_); CD (CH_3_CN, *c* 2.2 × 10^−^^4^) λ_max_ (millidegree), 230 (−3.73) nm; UV (CH_3_CN), λ_max_ (log ε): 198 (3.25), 222 (sh, 2.64) nm; IR (KBr) ν_max_: 3396, 2944, 2877, 1739, 1455, 1376, 1264, 1199, 1164, 1058, 1009, 904 cm^−1^; ^1^H NMR (CDCl_3_, 500 MHz) and ^13^C NMR (CDCl_3_, 125 MHz) data, see Table 1 and Table 2; HRESIMS *m*/*z* 377.2301 (calcd. for C_20_H_34_O_5_Na, 377.2299).

Diacardiol B (**6**): colorless oil; [α]^20^_D_ −2.4 (*c* 0.50, CHCl_3_); UV (CH_3_CN), λ_max_ (log ε): 210 (3.73) nm; IR (KBr) ν_max_: 3319, 3208, 2949, 2927, 2870, 1740, 1465, 1435, 1358, 1199, 1167, 1123, 1087, 1043, 1025, 983, 968, 908, 886, 853, 754 cm^−1^; ^1^H NMR (CDCl_3_, 500 MHz) and ^13^C NMR (CDCl_3_, 125 MHz) data, see Table 1 and Table 2; HRESIMS *m*/*z* 363.2506 (calcd. for C_20_H_36_O_4_Na, 363.25058).

Nuapapuin A methyl ester: Colorless oil; [α]^20^_D_ +71.2 (*c* 0.67, CHCl_3_); ^1^H NMR (CDCl_3_, 500 MHz): δ 2.58 (1H, q, *J* = 7.5 Hz, H-2), 4.27 (1H, td, *J* = 8.5, 3.0 Hz, H-3), 1.17 (3H, d, *J* = 7.5 Hz, 2-Me), 1.16 (3H, s, 6-Me), 3.70 (3H, OCH_3_); ^13^C NMR (CDCl_3_, 125 MHz): δ 174.3 (C-1), 42.5 (C-2), 81.0 (C-3), 32.8 (C-4), 35.1 (C-5), 80.2 (C-6), 22.5 (C-7), 22.2 (C-8), 136.7 (C-9), 127.1 (C-10), 32.7 (C-11), 19.6 (C-12), 39.9 (C-13), 34.8 (C-14), 12.4 (C-15), 23.6 (C-16), 19.7 (C-17), 28.5 (C-18), 28.7 (C-19), 51.9 (OCH_3_).

### 3.4. Reductive Cleavage of Nuapapuin A Methyl Ester

A mixture of nuapapuin A methyl ester (30 mg, 88.6 μmol), HOAc (1.5 mL, 26.2 mmol) and zinc (900 mg, 13.8 mmol) in EtOAc (15 mL) was stirred at room temperature for 12 h. The crude reaction mixture was then filtered, evaporated and purified on a flash silica gel column eluted with *n*-hexane-EtOAc (1:1) to afford the corresponding diol **6** (27 mg, 89%) showing the identical proton NMR data and similar specific rotation, [α]^20^_D_ −2.0 (*c* 0.50, CHCl_3_), with **6**.

### 3.5. Preparation of (S)- and (R)-MTPA Esters of 6

Two samples of diacardiol B (**6**; 1.1 mg, 3.2 μmol and 0.9 mg, 2.6 μmol) were mixed with *S*-(+)- or *R*-(−)-MTPA-Cl (15 μL, 80.1 μmol), respectively, in freshly distilled dry pyridine (500 μL) and then stirred under N_2_ atmosphere at room temperature for 18 h, and the solvent was then removed *in vacuo*. The products were purified by using a minicolumn chromatography packed with silica gel (200 mesh; petroleum ether/EtOAc, 1:1, v/v) to afford *S*-(+)- and *R*-(−)-MTPA esters **6a** (0.7 mg, 78%) and **6b** (0.8 mg, 73%), respectively. The anisotropic analysis was done by acquiring the interfered resonances for ^1^H NMR (CDCl_3_, 500 MHz) of **6a**: δ 5.406 (1H, m, H-3), 3.598 (3H, s, COOMe), 2.866 (1H, m, H-2), 1.902 (1H, m, H-4a), 1.975 (1H, m, H-4b), 1.138 (3H, d, *J* = 6.1 Hz, H-15), and 1.157 (3H, s, H-16); and the interfered resonances for ^1^H NMR (CDCl_3_, 500 MHz) of **6b**: δ 5.394 (1H, m, H-3), 3.651 (3H, s, COOMe), 2.875 (1H, m, H-2), 1.894 (1H, m, H-4a), 1.933 (1H, m, H-4b), 1.190 (3H, d, *J* = 6.1 Hz, H-15), and 1.078 (3H, s, H-16).

### 3.6. Antimalarial Assay

Antimalarial activities were determined against chloroquine sensitive (D6, Sierra Leone) and resistant (W2, Indo China) strains of *Plasmodium falciparum* *in vitro* by measuring plasmodial LDH activity. Each testing group dissolved in DMSO with a concentration of 2 mg/mL. A 200-μL suspension of *P*. *falciparum culture* (2% parasitemia and 2% hematocrit in RPMI 1640 medium supplemented with 10% human serum and 60 μg/mL amikacin) was added to each well, containing 10 μL of serially diluted samples, of a 96-well plate. The plate was flushed with a gas mixture of 90% N_2_, 5% O_2_ and 5% CO_2_ and incubated at 37 °C for 72 h in a modular incubation chamber. Plasmodial LDH activity was determined by using Malstat reagent. Briefly, 20 μL of the incubation mixture were mixed with 100 μL of the Malstat reagent and incubated for 30 min. Twenty microliters of a 1:1 mixture of NBT/PES were then added, and the plate was further incubated in dark environment for 1 h. The test was ended by adding 100 μL of a 5% acetic acid solution. The plate was monitored at 650 nm. Artemisinin was used as the control drug.

### 3.7. MTT Cytotoxicity Assay

Briefly, human cancer cells in the exponential growth phase were harvested and then seeded into a flat-bottomed 96-well plate, and each well contained 2.0 × 10^3^ cells in 100 μL of solution. After incubation for 12 h in a 5% humidified CO_2_ incubator at 37 °C, the compound for testing was added (in triplicate experiments) at six different concentrations. After 72 h, incubating at 37 °C, 20 μL of MTT were added to each well, and the plate was incubated again at 37 °C for 3 h. Absorption was then measured by using a SpectraMAX 340 reader (Molecular Devices, Sunnyvale, CA, USA) at 550 nm, with a reference filter at 690 nm, and IC_50_ values were calculated on the basis of the percentage of inhibition using the linear regression method.

### 3.8. Computational Calculation

The MMFF (Merck molecular force field) minimized structures of Compounds **1**–**5** were used for the DFT calculations. DFT calculations were performed by applying the PCM solvation models with the dielectric constant representing acetonitrile by using the Gaussian 09 program (Revision A.1, Gaussian, Inc., Wallingford, CT, USA, 2009). Low energy conformations were optimized by using the B3LYP/6-31G(d,p) or MPWLPW91/6-31G(d,p) methods applying the PCM solvation model, and the frequency calculations were performed at the same theoretical levels to verify the true located energy minimal and to generate sets of thermodynamic data at standard condition. The optimized geometries were used for TDDFT calculations at the B3LYP/6-31G(d,p) level applying the same solvation model. The generated excitation energies and rotational strengths were Boltzmann averaged and then fitted to Gaussian functions to generate computed ECD spectra normalized and overlaid with the experimental spectra for comparison [28,29]. The NMR calculations were performed by using the Gauge-invariant atomic orbital (GIAO) method at the B3LYP/6-31G(d,p) and MPW1PW91/6-31G(d,p) levels, and the calculated chemical shifts for TMS at the corresponding levels were used as references (computational details for Compounds **1**–**5** are provided in the Supplementary Information).

## 4. Conclusions

The diacarperoxide class was likely generated from the terpene biosynthetic pathway, and the endoperoxyl functional group was probably formed by reacting singlet oxygen with an isoprene unit through a Diels–Alder reaction or intramolecular 1,4-addition from a peroxy nucleophile to an α,β-unsaturated carbonyl. These availabilities have been demonstrated synthetically [30,31]. The diacarperoxide class provides us potent compounds acting against malaria. The peroxy functional group suggests a potential pharmacophore of these compounds, which inspires further synthetic and biological study. The utilization of diverse computational approaches proved an efficient and reliable way to solve stereochemistry issues related to natural products.

## Supplementary Files

Supplementary File 1
