# Supplementary material for: Relative and Absolute Stereochemistry of Diacarperoxides: Antimalarial Norditerpene Endoperoxides from Marine Sponge Diacarnus megaspinorhabdosa
Source: Mar Drugs. 2014 Aug 8;12(8):4399–416. doi: 10.3390/md12084399 (PMC4145323; doi:10.3390/md12084399)
Supplement: Supplementary File 1 [file marinedrugs-12-04399-s001.pdf]

Supplementary Information

Figure S1. High resolution ESI mass spectrum of diacarperoxide H (**1**).
Figure S2. IR spectrum of diacarperoxide H (**1**).
Figure S3. ^{1}H NMR spectrum of diacarperoxide H (**1**) in $CDCl_3$.
Figure S4. ^{13}C NMR spectrum of diacarperoxide H (**1**) in $CDCl_3$.
Figure S5. ^{1}H-^{1}H COSY spectrum of diacarperoxide H (**1**) $CDCl_3$.
Figure S6. HSQC spectrum of diacarperoxide H (**1**) in $CDCl_3$.
Figure S7. HMBC spectrum of diacarperoxide H (**1**) in $CDCl_3$.
Figure S8. NOESY spectrum of diacarperoxide H (**1**) in $CDCl_3$.
Figure S9. High resolution ESI mass spectrum of diacarperoxide I (**2**).
Figure S10. IR spectrum of diacarperoxide I (**2**).
Figure S11. ^{1}H NMR spectrum of diacarperoxide I (**2**) in $CDCl_3$.
Figure S12. ^{13}C NMR spectrum of diacarperoxide I (**2**) in $CDCl_3$.
Figure S13. ^{1}H-^{1}H COSY spectrum of diacarperoxide I (**2**) $CDCl_3$.
Figure S14. HSQC spectrum of diacarperoxide I (**2**) in $CDCl_3$.
Figure S15. HMBC spectrum of diacarperoxide I (**2**) in $CDCl_3$.
Figure S16. NOESY spectrum of diacarperoxide I (**2**) in DMSO.
Figure S17. High resolution ESI mass spectrum of diacarperoxide J (**3**).
Figure S18. IR spectrum of diacarperoxide J (**3**).
Figure S19. ^{1}H NMR spectrum of diacarperoxide J (**3**) in $CDCl_3$.
Figure S20. ^{13}C NMR spectrum of diacarperoxide J (**3**) in $CDCl_3$.
Figure S21. ^{1}H-^{1}H COSY spectrum of diacarperoxide J (**3**) $CDCl_3$.
Figure S22. HSQC spectrum of diacarperoxide J (**3**) in $CDCl_3$.
Figure S23. HMBC spectrum of diacarperoxide J (**3**) in $CDCl_3$.
Figure S24. NOESY spectrum of diacarperoxide J (**3**) in DMSO.
Figure S25. High resolution ESI mass spectrum of diacarperoxide K (**4**).
Figure S26. IR spectrum of diacarperoxide K (**4**).
Figure S27. ^{1}H NMR spectrum of diacarperoxide K (**4**) in $CDCl_3$.
Figure S28. ^{13}C NMR spectrum of diacarperoxide K (**4**) in $CDCl_3$.
Figure S29. ^{1}H-^{1}H COSY spectrum of diacarperoxide K (**4**) $CDCl_3$.
Figure S30. HSQC spectrum of diacarperoxide K (**4**) in $CDCl_3$.
Figure S31. HMBC spectrum of diacarperoxide K (**4**) in $CDCl_3$.
Figure S32. NOESY spectrum of diacarperoxide K (**4**) in $CDCl_3$.
Figure S33. High resolution ESI mass spectrum of diacarperoxide L (**5**).
Figure S34. IR spectrum of diacarperoxide L (**5**).
Figure S35. ^{1}H NMR spectrum of diacarperoxide L (**5**) in $CDCl_3$.
Figure S36. ^{13}C NMR spectrum of diacarperoxide L (**5**) in $CDCl_3$.
Figure S37. ^{1}H-^{1}H COSY spectrum of diacarperoxide L (**5**) $CDCl_3$.
Figure S38. HSQC spectrum of diacarperoxide L (**5**) in $CDCl_3$.
Figure S39. HMBC spectrum of diacarperoxide L (**5**) in $CDCl_3$.
Figure S40. NOESY spectrum of diacarperoxide L (**5**) in $CDCl_3$.
Figure S41. High resolution ESI mass spectrum of diacardiol B (**6**).

Figure S42. IR spectrum of diacardiol B (**6**).
Figure S43. ^{1}H NMR spectrum of diacardiol B (**6**) in $CDCl_3$.
Figure S44. ^{13}C NMR spectrum of diacardiol B (**6**) in $CDCl_3$.
Figure S45. ^{1}H-^{1}H COSY spectrum of diacardiol B (**6**) $CDCl_3$.
Figure S46. HSQC spectrum of diacardiol B (**6**) in $CDCl_3$.
Figure S47. HMBC spectrum of diacardiol B (**6**) in $CDCl_3$.
Figure S48. NOESY spectrum of diacardiol B (**6**) in $CDCl_3$.
Figure S49. ^{1}H NMR spectrum of (*S*)-MTPA ester **6a** in $CDCl_3$.
Figure S50. ^{1}H NMR spectrum of (*R*)-MTPA ester **6b** in $CDCl_3$.
Figure S51. ^{1}H NMR spectrum of diol derived from nuapapuin A methyl ester in $CDCl_3$.
Figure S52. Dominate conformations of compounds **1**–**5**.
Figure S53. Calculated ECD spectra of **1**.
Figure S54. Calculated ECD spectra of **2**.
Figure S55. Calculated ECD spectra of **3**.
Figure S56. Calculated ECD spectra of **4**.
Figure S57. Calculated ECD spectra of **5**.
Table S1. Calculated thermodynamic data of **1**.
Table S2. Calculated relative Boltzmann populations of **1**.
Table S3. Calculated thermodynamic data of **2**.
Table S4. Calculated relative Boltzmann populations of **2**.
Table S5. Calculated thermodynamic data of **3**.
Table S6. Calculated relative Boltzmann populations of **3**.
Table S7. Calculated thermodynamic data of **4**.
Table S8. Calculated relative Boltzmann populations of **4**.
Table S9. Calculated thermodynamic data of **5**.
Table S10. Calculated relative Boltzmann populations of **5**.
Table S11. Cartesian coordinates of the lowest energy conformation of **1**–**5**.
Figure S58. A-value study of the endoperoxide core on **type I** and **type II** model.
Table S12. Calculated thermodynamic data of **type I** and **type II** model.
Table S13. Cartesian coordinates of the lowest energy conformation of **type I** model.
Table S14. Cartesian coordinates of the lowest energy conformation of **type II** model.
Figure S59. Study of the 1,3-diaxial interaction *vs.* 1,3-allylic strain on the model **m1** and **m2**.
Table S15. Calculated thermodynamic data of the model **m1** and **m2**.
Table S16. Cartesian coordinates of the lowest energy conformations of **m1** and **m2**.
Table S17. Bioactivities of the compound **1**–**6** against A549 (lung carcinoma).
Table S18. Bioactivities of the compound **1**–**6** against HeLa (cervical cancer).
Table S19. Bioactivities of the compound **1**–**6** against QGY-7703 (hepatocarcinoma).
Table S20. Bioactivities of the compound **1**–**6** against MDA-MB-231 (human breast adenocarcinoma).

Figure S1. High resolution ESI mass spectrum of diacarperoxide H (**1**).

Mass Spectrum Molecular Formula Report

Analysis Info

Analysis Name	D:\Data\20110707\WOL-72.d	Acquisition Date	7/7/2011 10:28:25 AM
Method	tune_50-1000_POS_20110328.m	Operator	YP
Sample Name	WOL-72	Instrument / Ser#	micrOTOF-Q 125
Comment			

Acquisition Parameter

Source Type	ESI	Ion Polarity	Positive	Set Nebulizer	0.5 Bar
Focus	Not active	Set Capillary	4500 V	Set Dry Heater	180 °C
Scan Begin	50 m/z	Set End Plate Offset	-500 V	Set Dry Gas	4.0 l/min
Scan End	3000 m/z	Set Collision Cell RF	250.0 Vpp	Set Divert Valve	Source

Generate Molecular Formula Parameter

Formula, min.	C20H32Na1O6				
Formula, max.					
Measured m/z	391.209	Tolerance	5 ppm	Charge	1
Check Valence	no	Minimum	0	Maximum	0
Nirogen Rule	no	Electron Configuration	both		
Filter H/C Ratio	no	Minimum	0	Maximum	3
Estimate Carbon	yes				

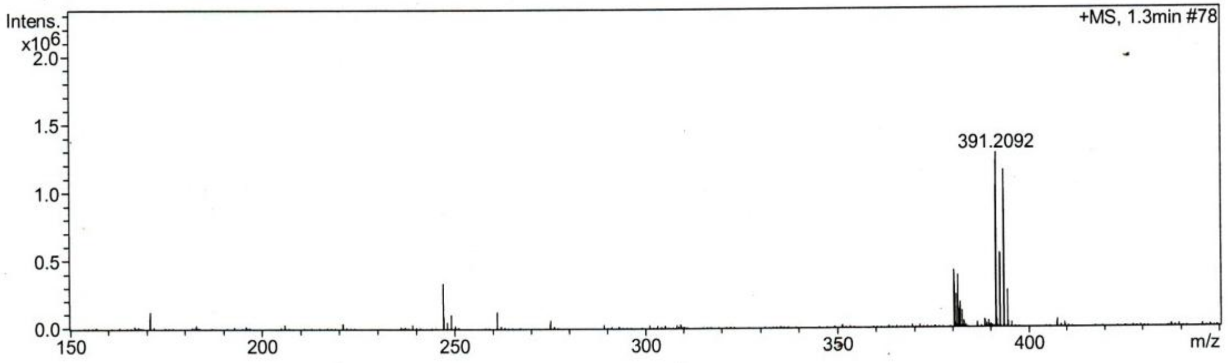

#	m/z	I
1	391.2092	1274688

Sum Formula	Sigma	m/z	Err [ppm]	Mean Err [ppm]	Err [mDa]	rdb	N Rule	e⁻
C 20 H 32 Na 1 O 6	0.130	391.2091	-0.20	-0.20	-0.08	4.50	ok	even

Figure S2. IR spectrum of diacarperoxide H (**1**).

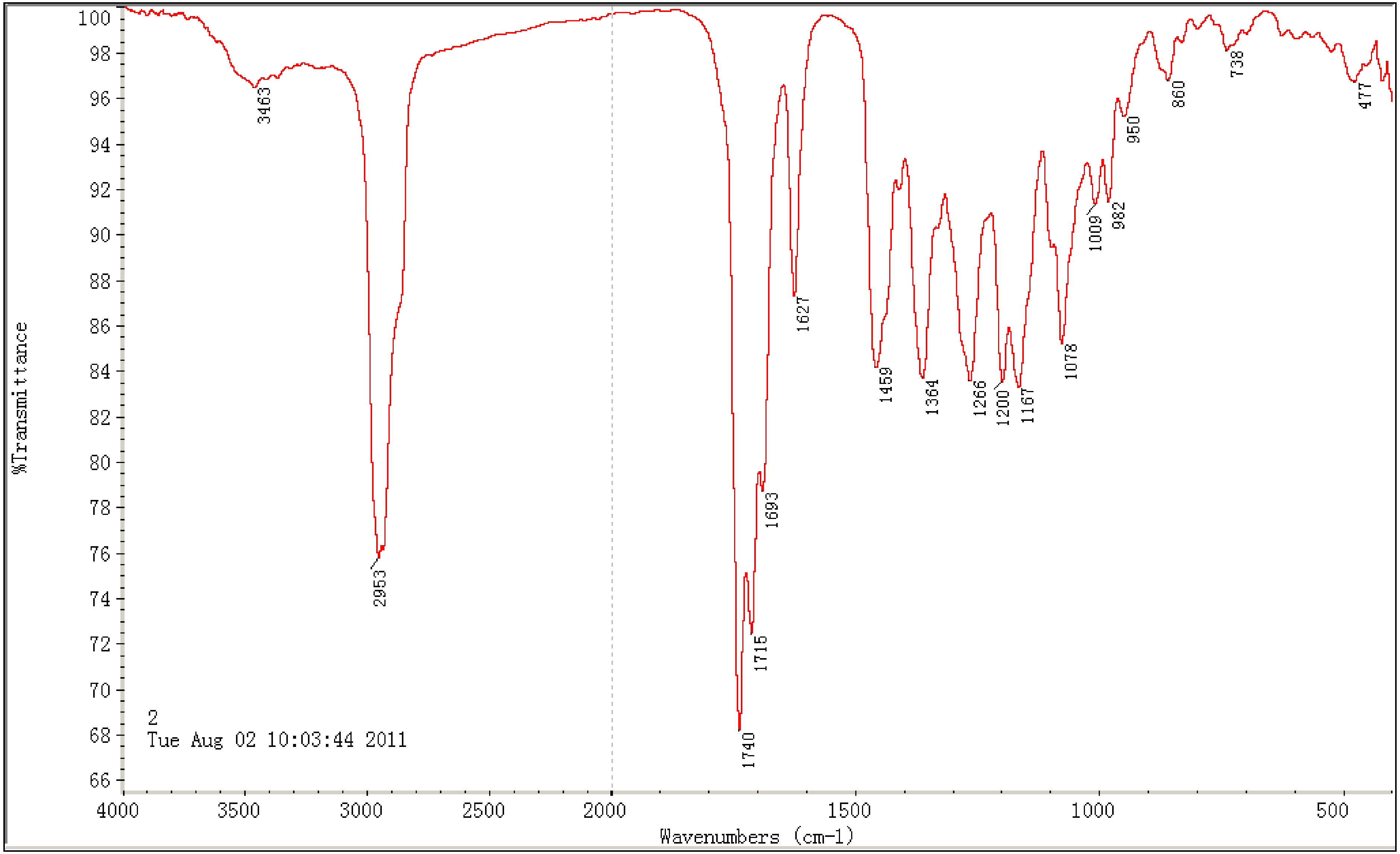

Figure S3. ^{1}H NMR spectrum of diacarperoxide H (**1**) in $CDCl_3$.

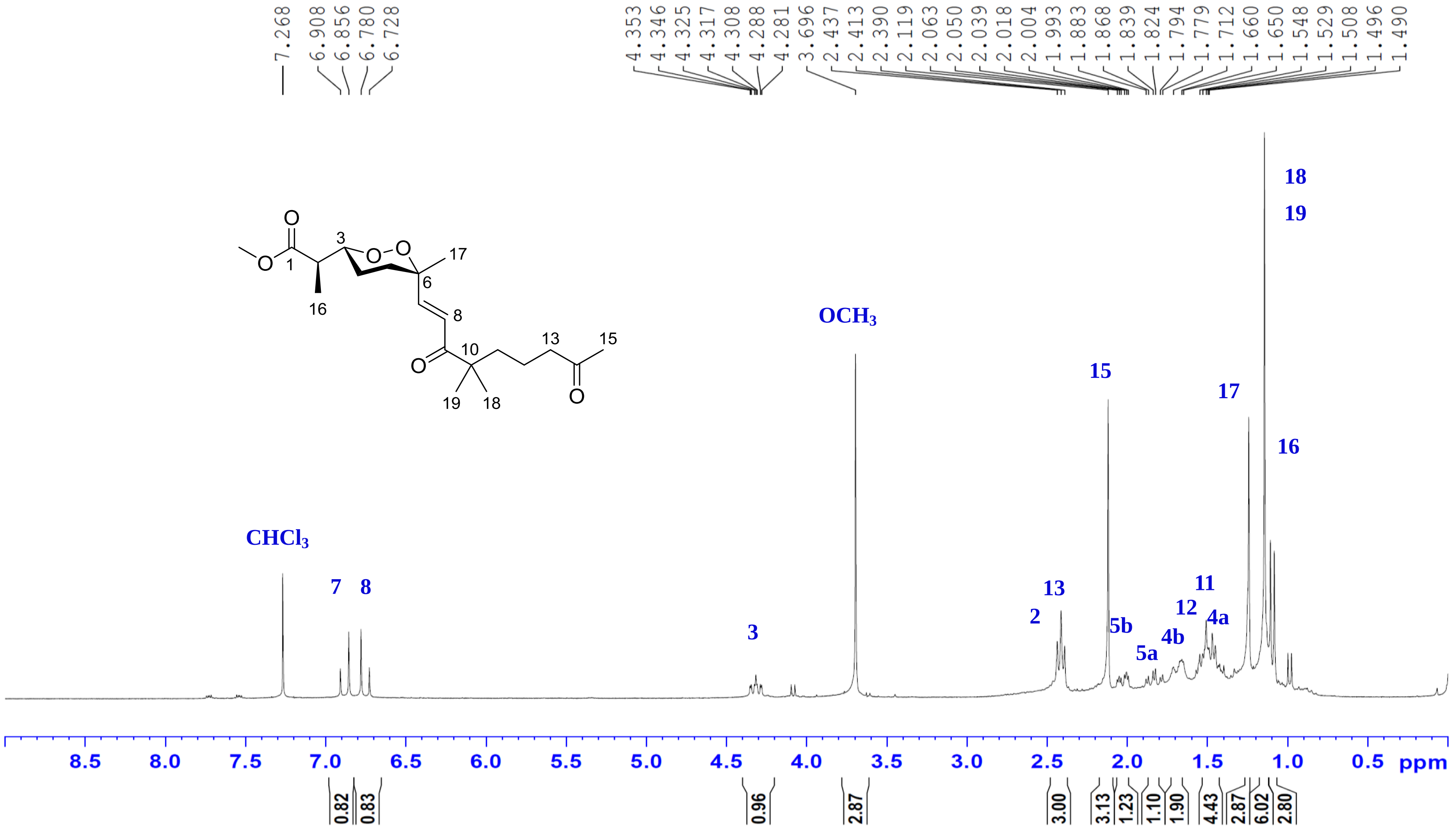

Figure S4. ^{13}C NMR spectrum of diacarperoxide H (**1**) in $CDCl_3$.

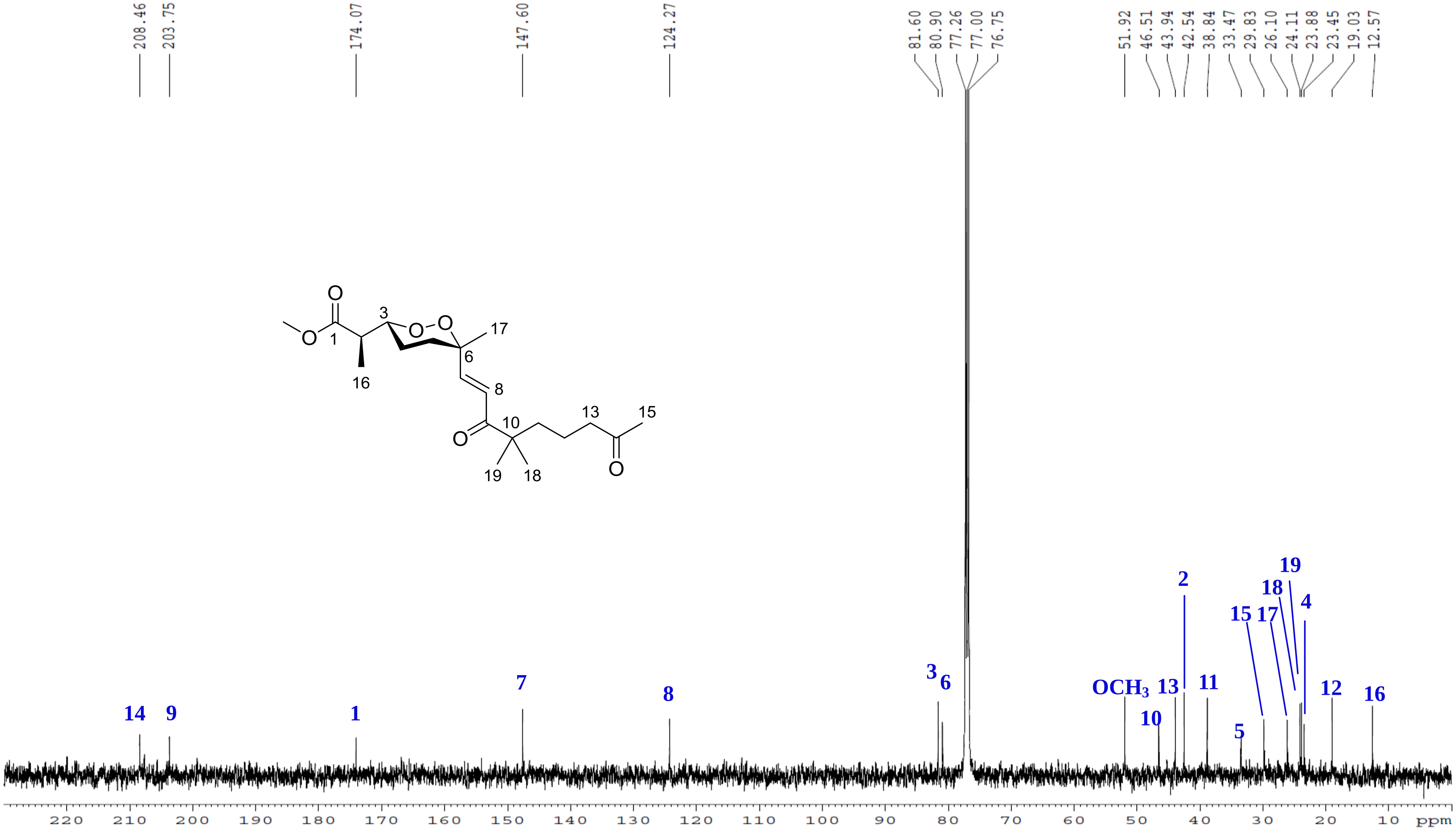

Figure S5. ^{1}H-^{1}H COSY spectrum of diacarperoxide H (**1**) in $CDCl_3$.

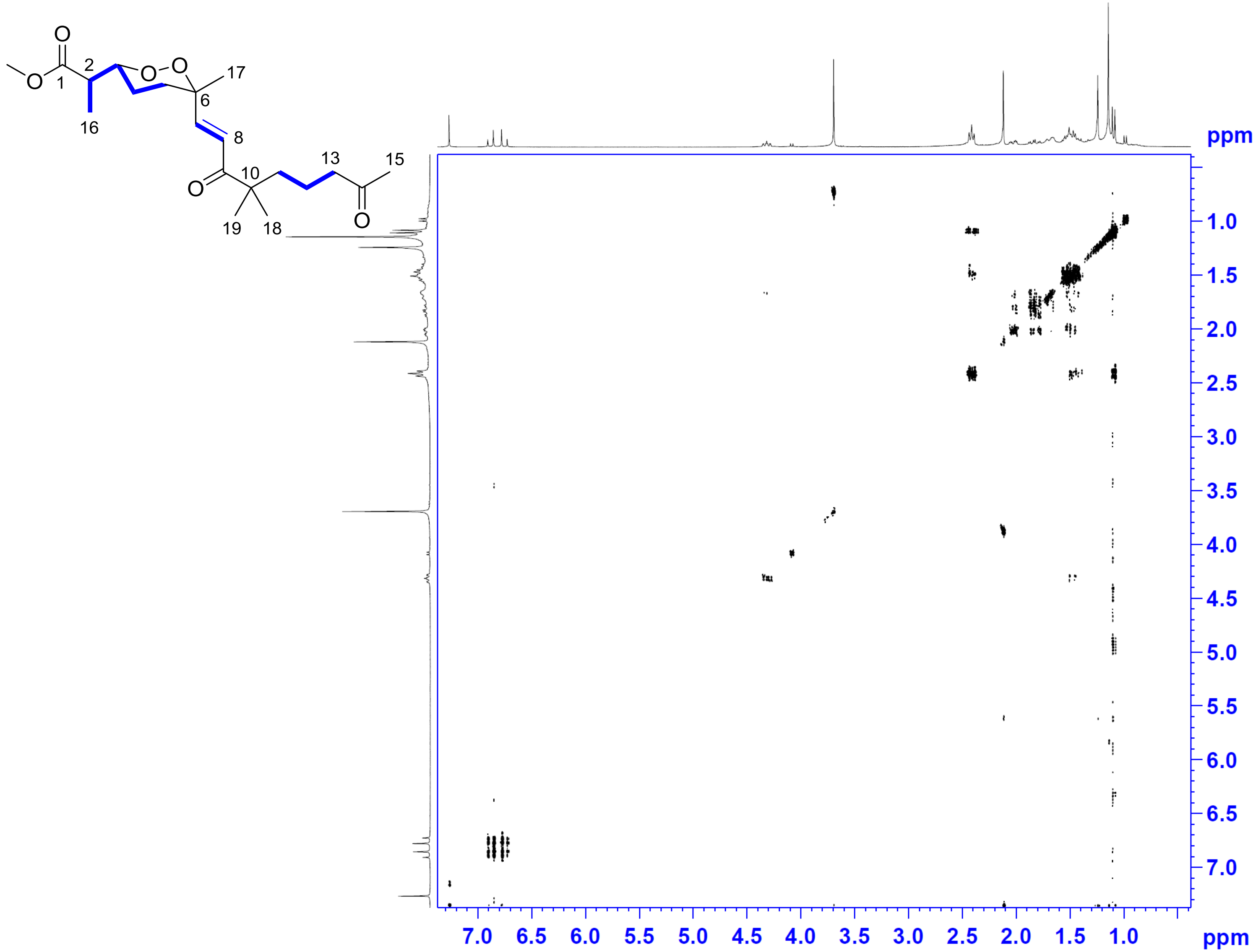

Figure S6. HSQC spectrum of diacarperoxide H (**1**) in $CDCl_3$.

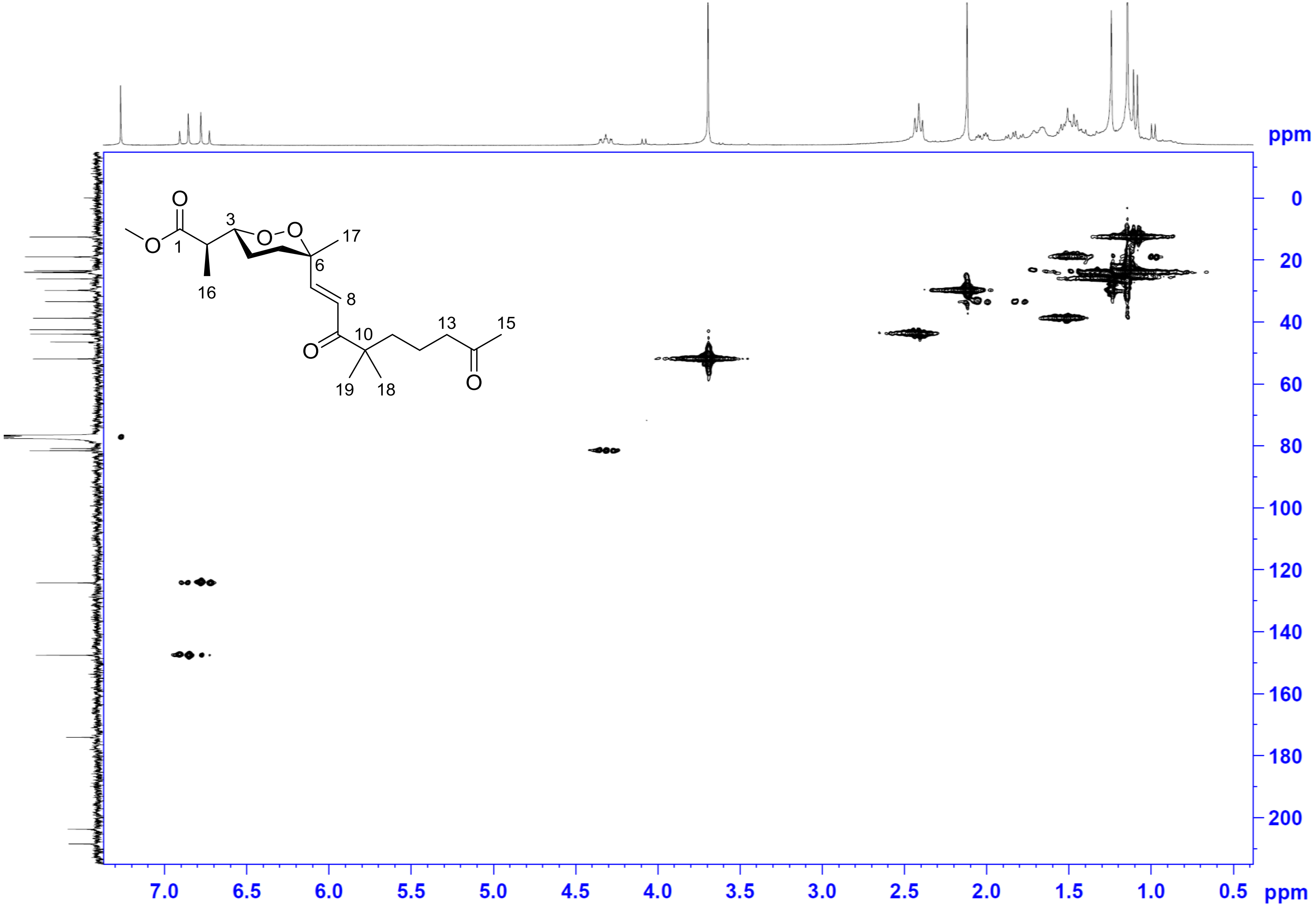

Figure S7. HMBC spectrum of diacarperoxide H (**1**) in $CDCl_3$.

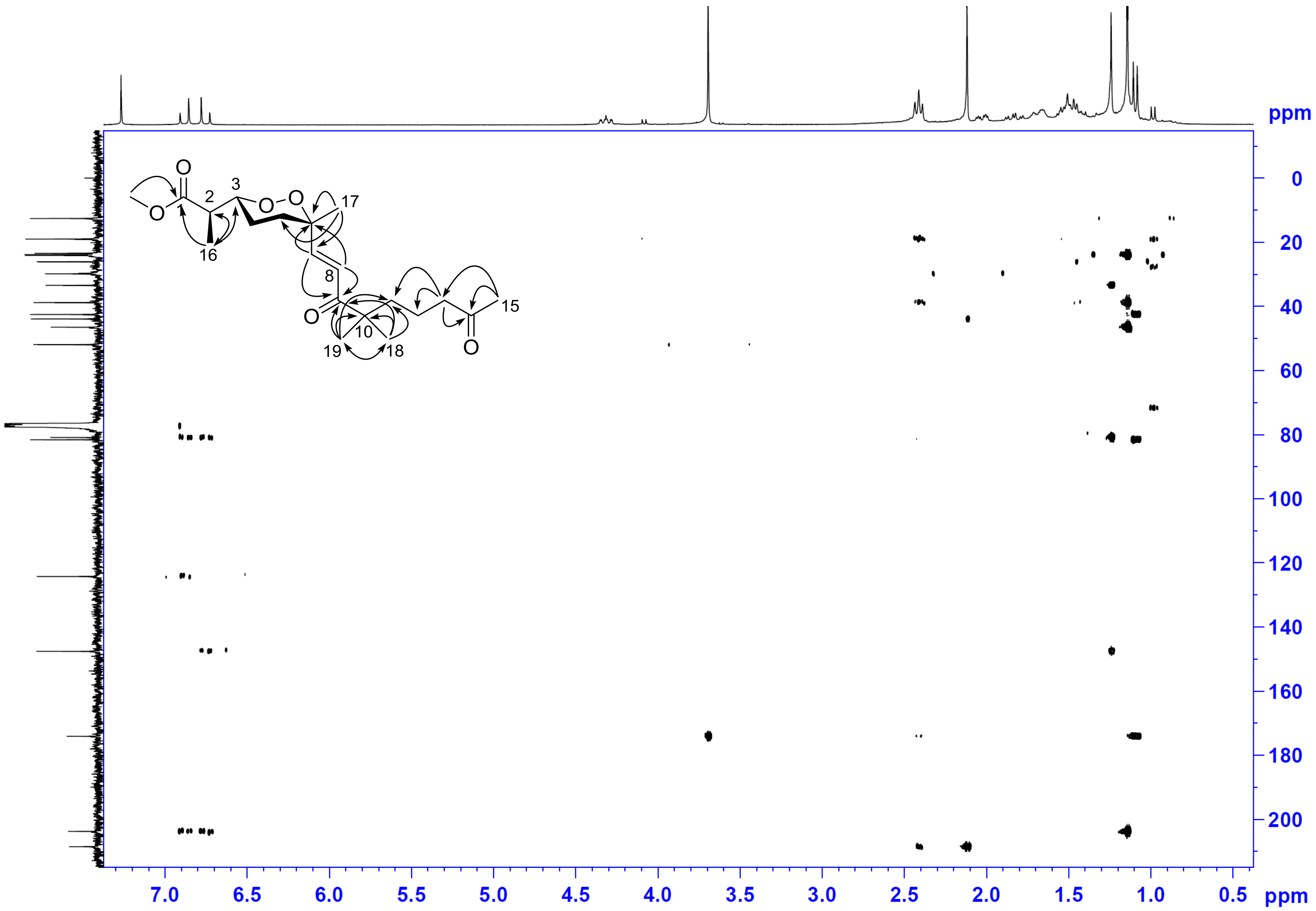

Figure S8. NOESY spectrum of diacarperoxide H (**1**) in $CDCl_3$.

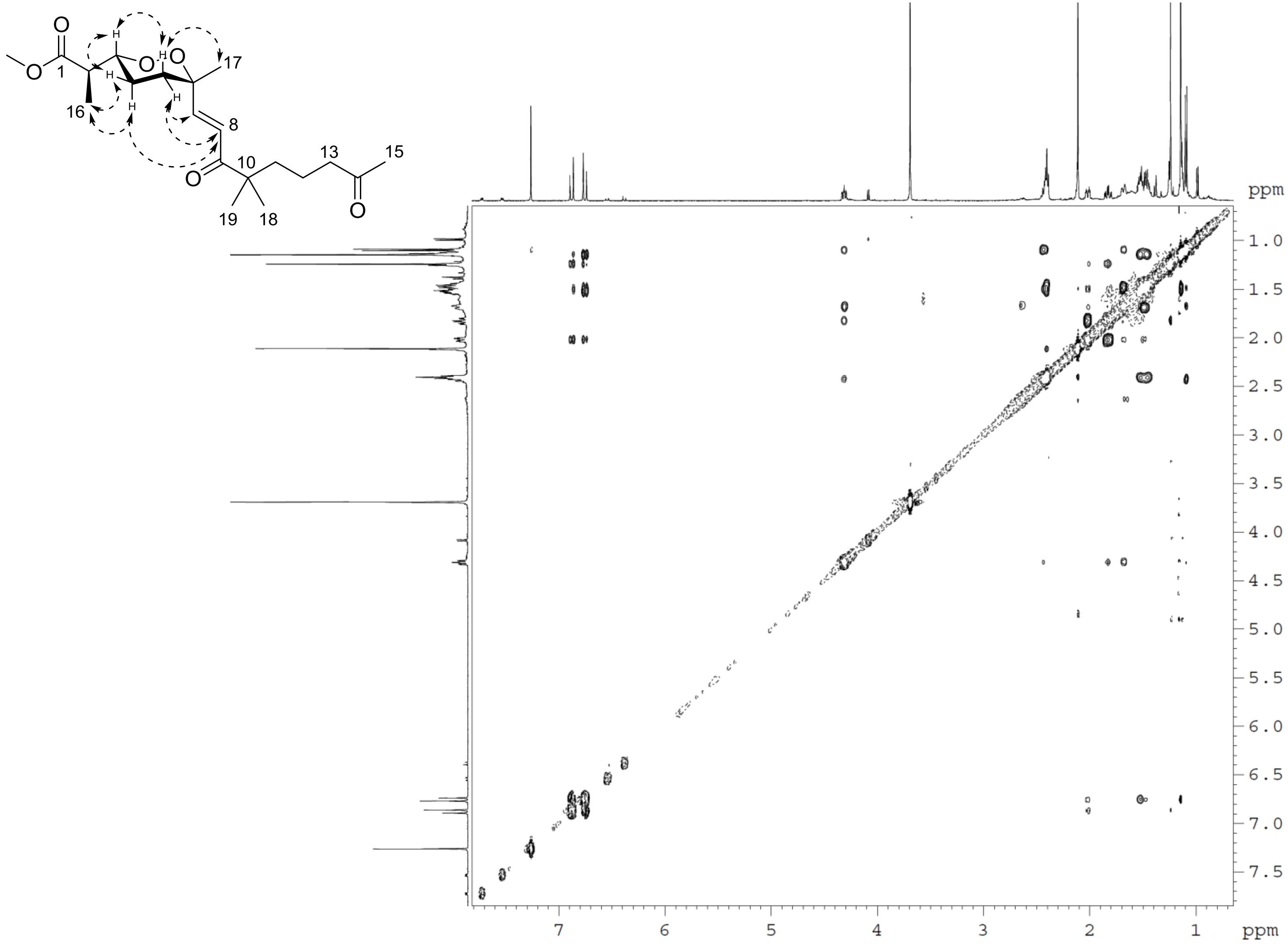

Figure S9. High resolution ESI mass spectrum of diacarperoxide I (**2**).

Mass Spectrum Molecular Formula Report

Analysis Info

Analysis Name	D:\Data\20110707\WOL-50.d	Acquisition Date	7/7/2011 10:36:42 AM
Method	tune_50-1000_POS_20110328.m	Operator	YP
Sample Name	WOL-50	Instrument / Ser#	micrOTOF-Q 125
Comment			

Acquisition Parameter

Source Type	ESI	Ion Polarity	Positive	Set Nebulizer	0.5 Bar
Focus	Not active	Set Capillary	4500 V	Set Dry Heater	180 °C
Scan Begin	50 m/z	Set End Plate Offset	-500 V	Set Dry Gas	4.0 l/min
Scan End	3000 m/z	Set Collision Cell RF	250.0 Vpp	Set Divert Valve	Source

Generate Molecular Formula Parameter

Formula, min.	C20H34Na1O5				
Formula, max.					
Measured m/z	377.23	Tolerance	5 ppm	Charge	1
Check Valence	no	Minimum	0	Maximum	0
Nirogen Rule	no	Electron Configuration	both		
Filter H/C Ratio	no	Minimum	0	Maximum	3
Estimate Carbon	yes				

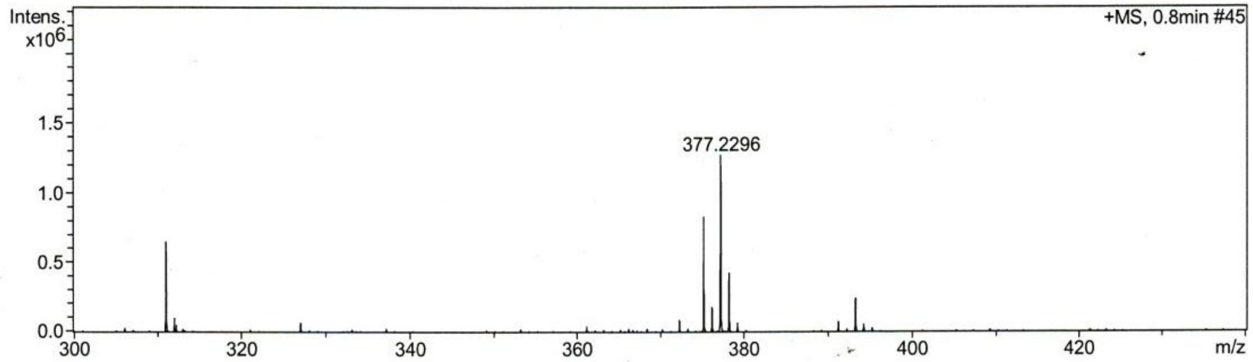

#	m/z	I
1	377.2296	1268272

Sum Formula	Sigma	m/z	Err [ppm]	Mean Err [ppm]	Err [mDa]	rdb	N Rule	e⁻
C 20 H 34 Na 1 O 5	0.130	377.2298	0.68	0.68	0.26	3.50	ok	even

Figure S10. IR spectrum of diacarperoxide I (**2**).

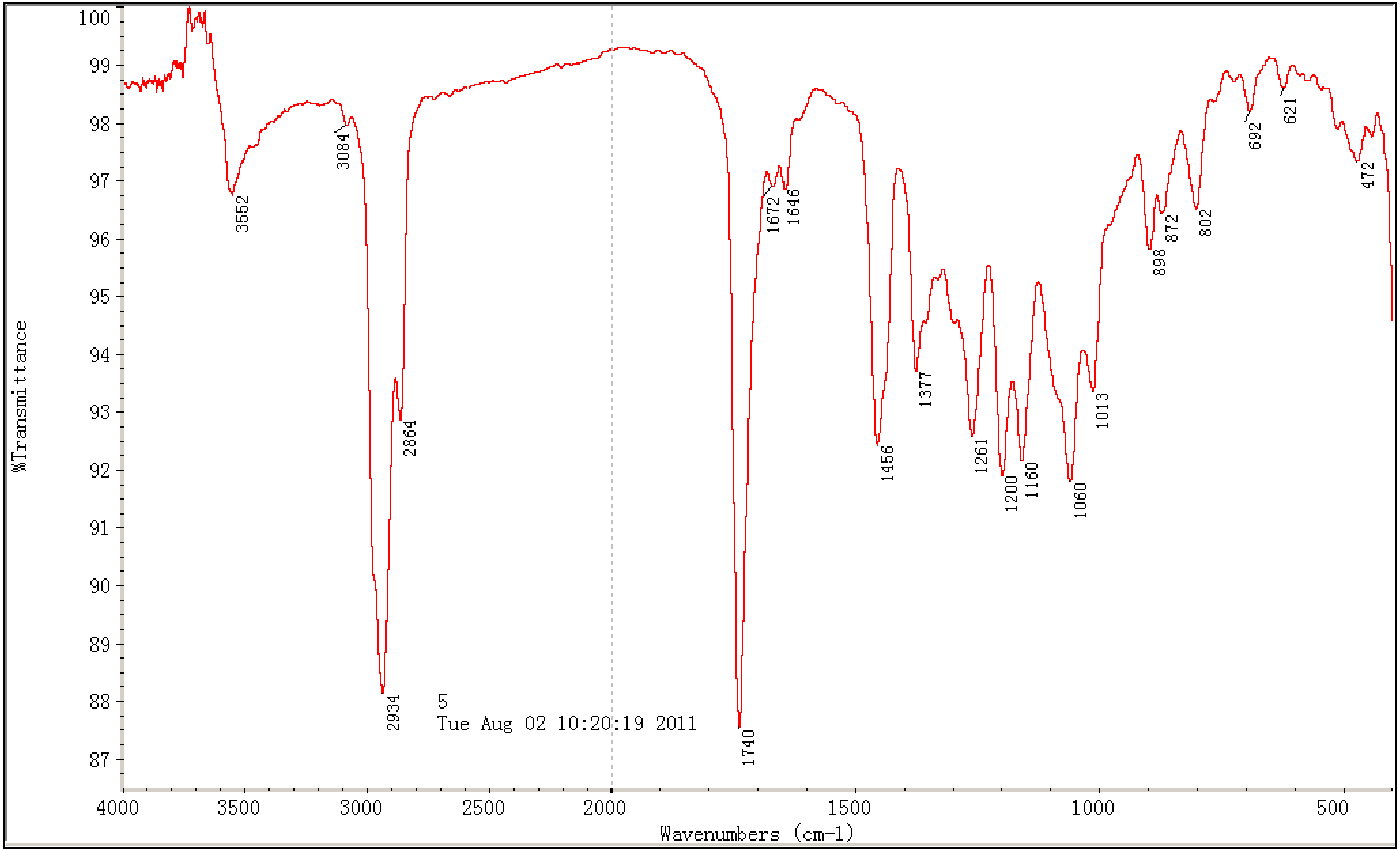

Figure S11. ^{1}H NMR spectrum of diacarperoxide I (**2**) in $CDCl_3$.

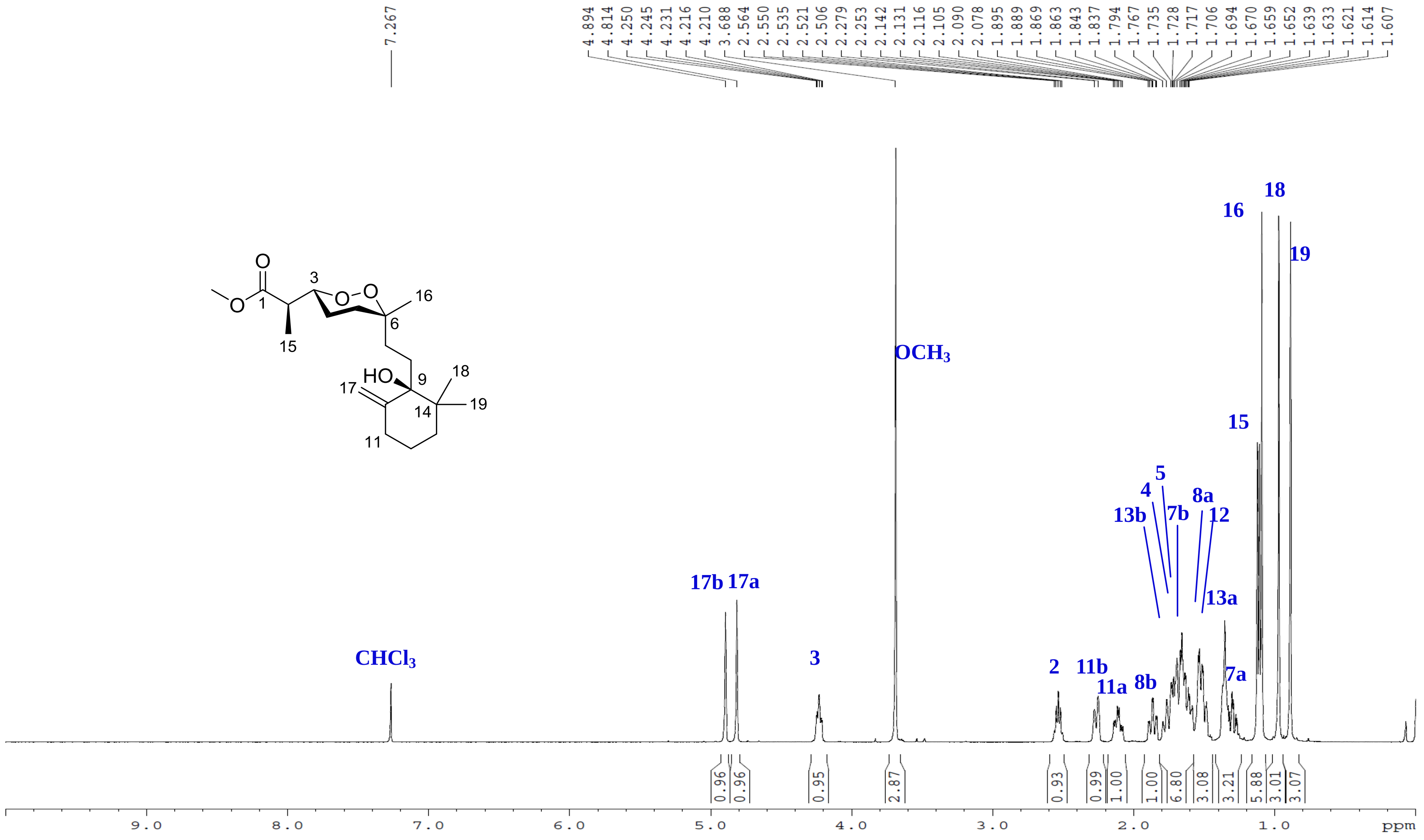

Figure S12. ^{13}C NMR spectrum of diacarperoxide I (**2**) in $CDCl_3$.

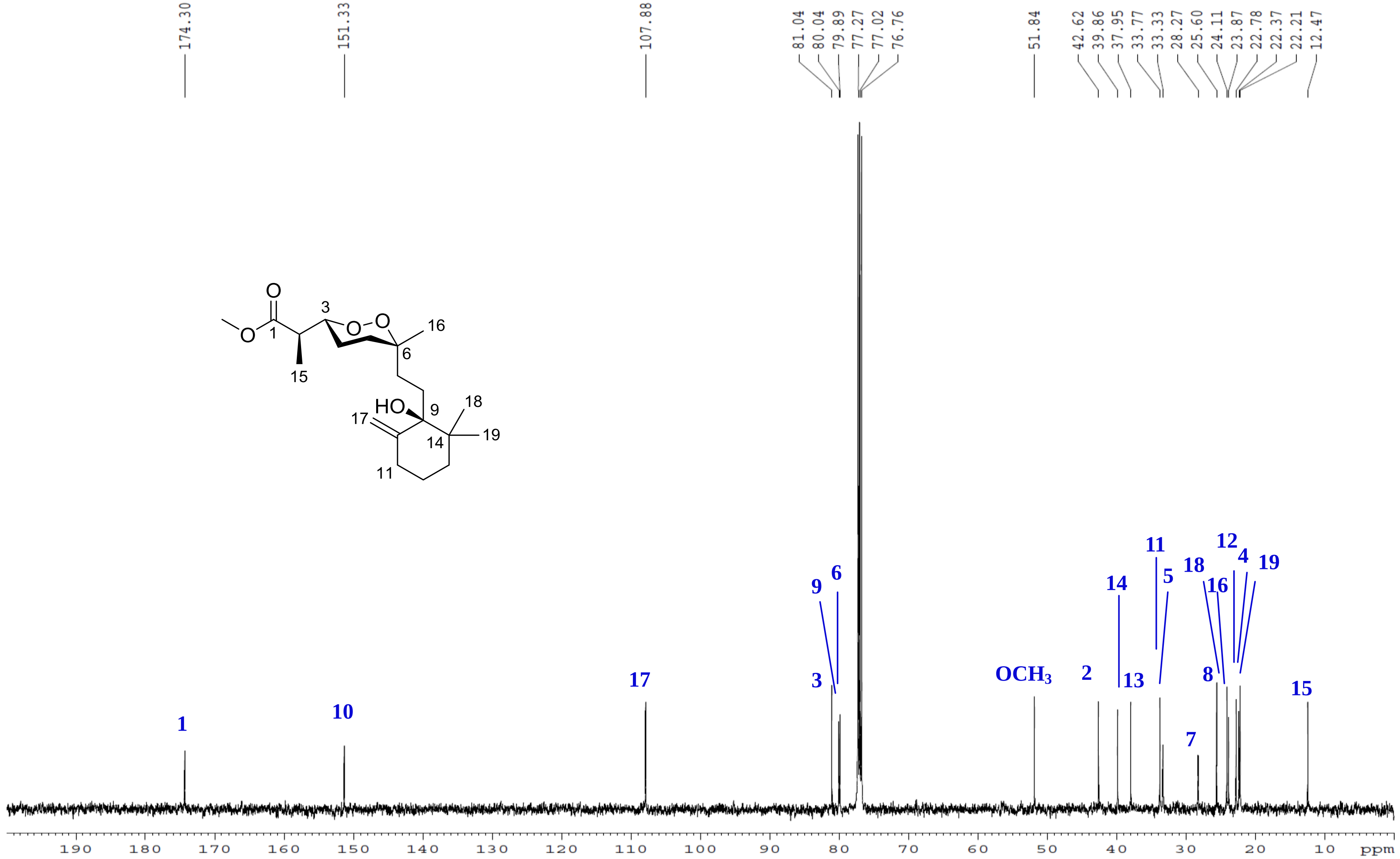

Figure S13. ^{1}H-^{1}H COSY spectrum of diacarperoxide I (**2**) in $CDCl_3$.

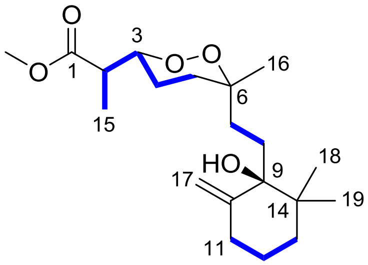

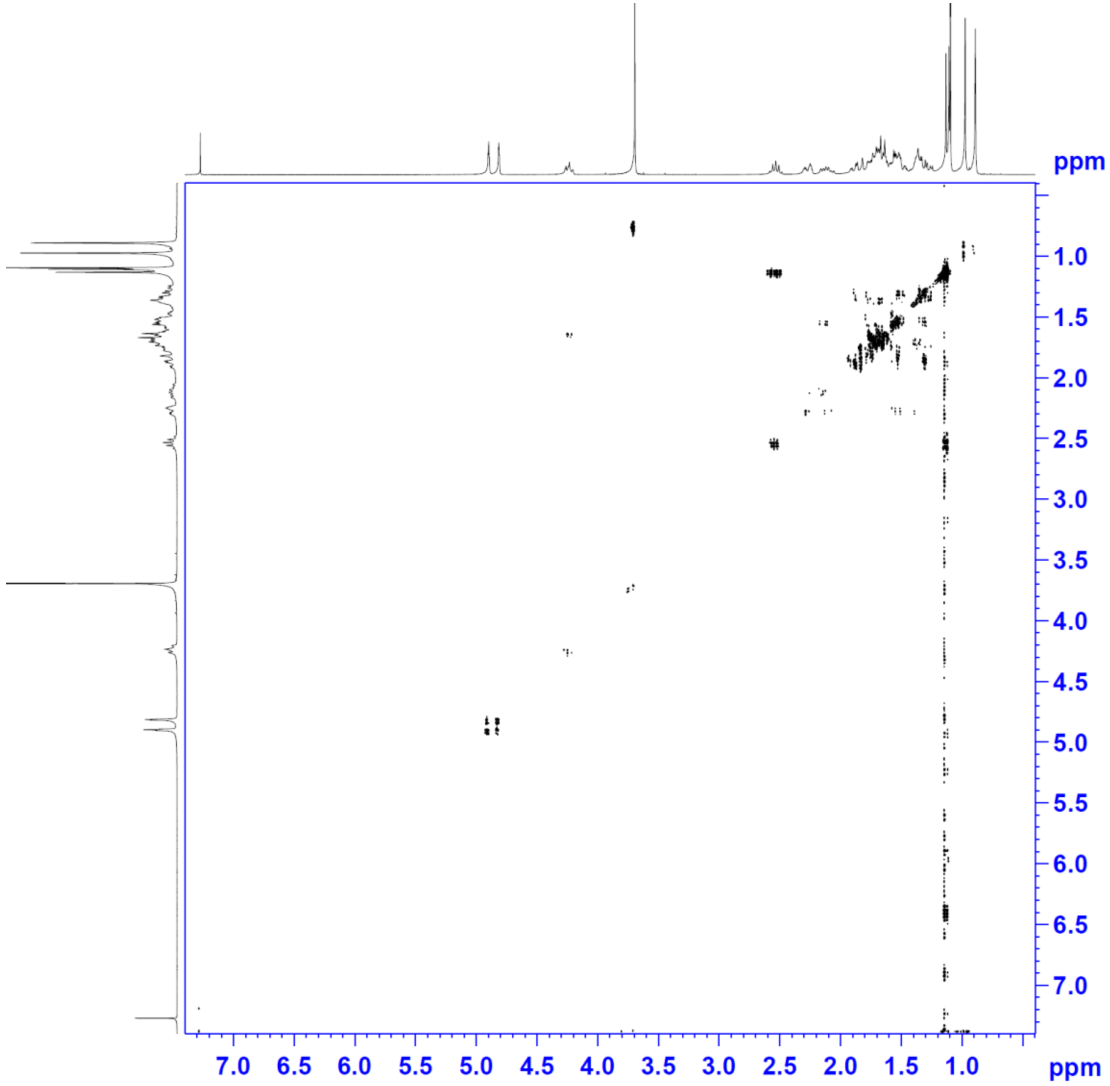

Figure S14. HSQC spectrum of diacarperoxide I (**2**) in $CDCl_3$.

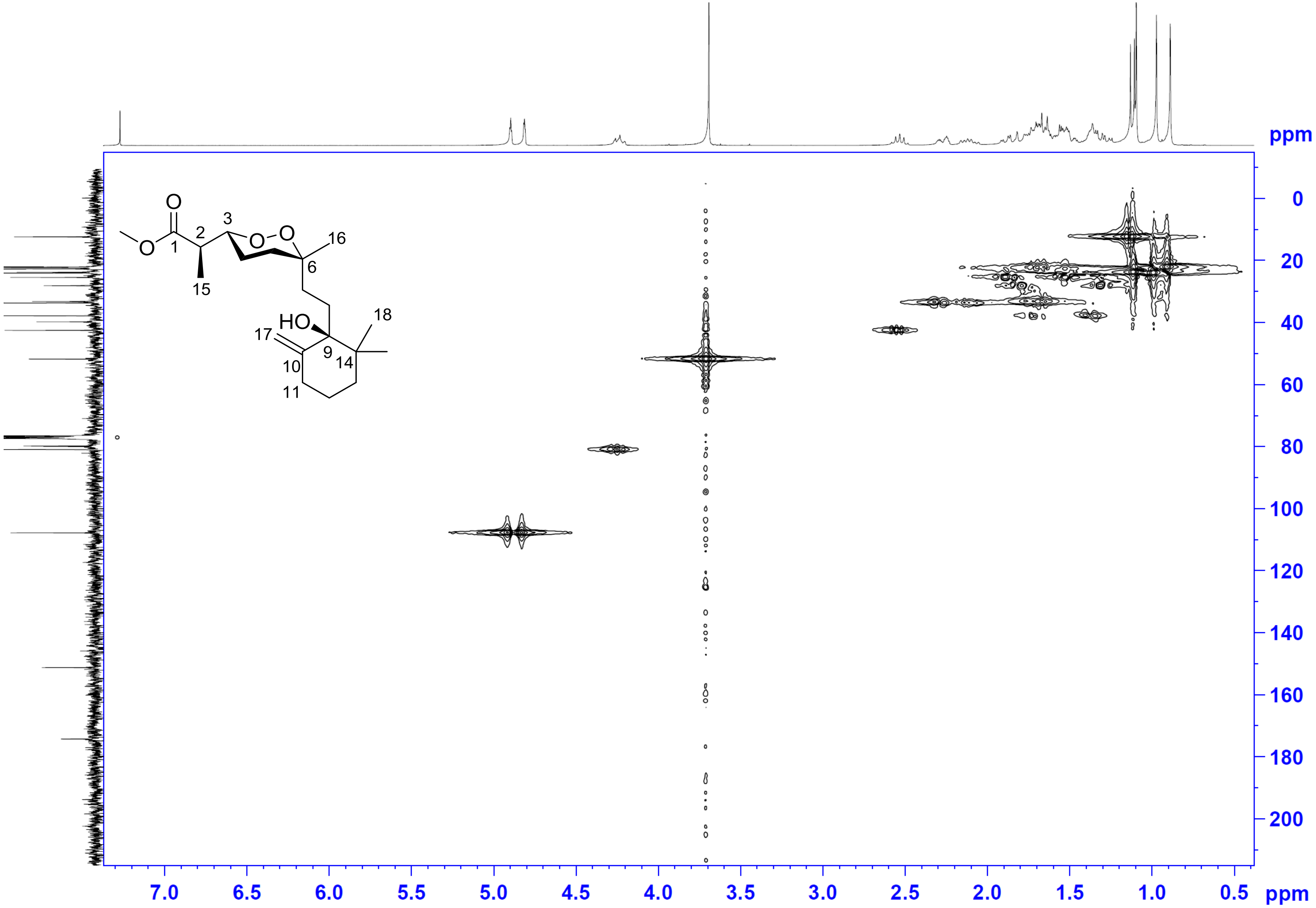

Figure S15. HMBC spectrum of diacarperoxide I (**2**) in $CDCl_3$.

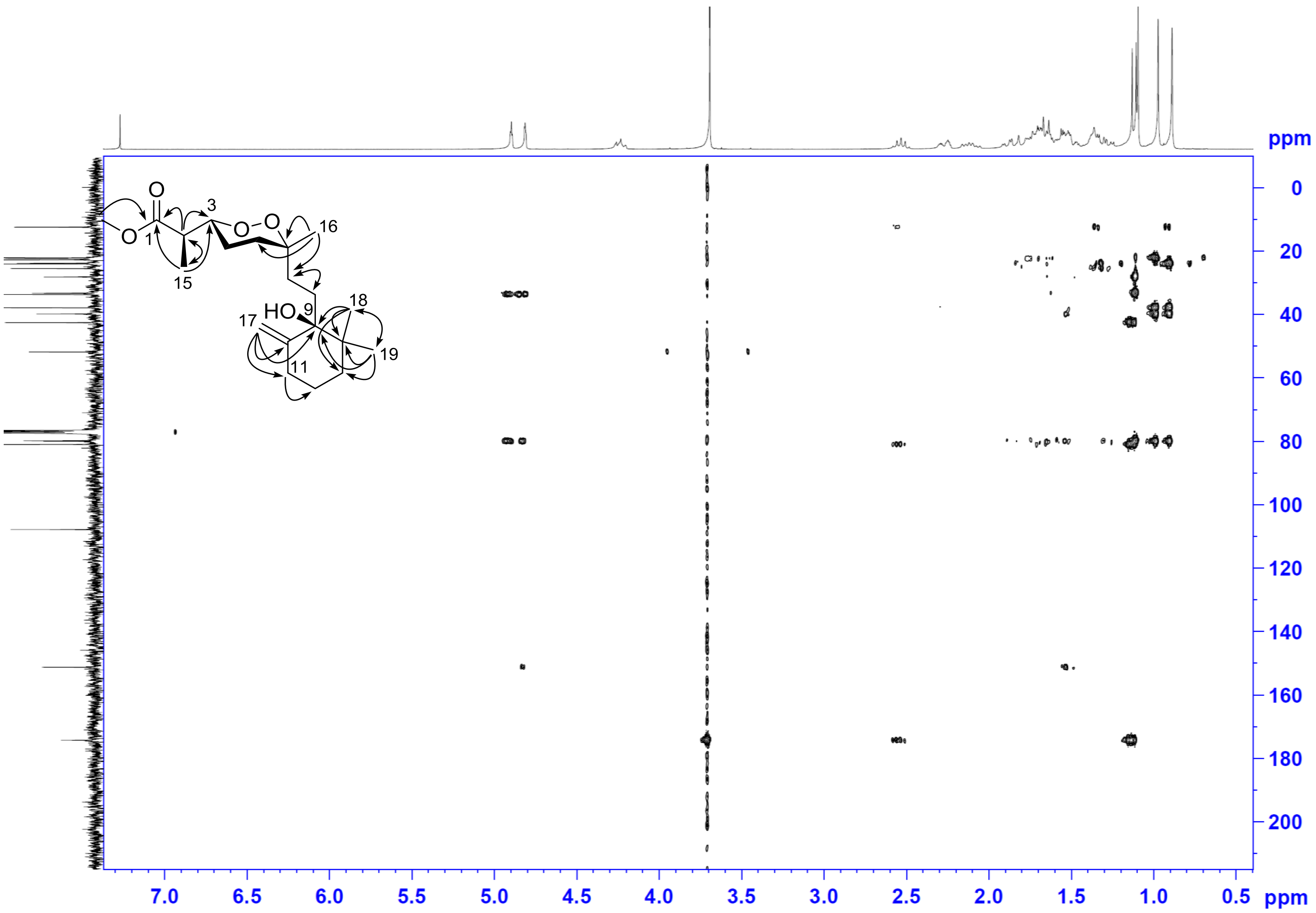

Figure S16. NOESY spectrum of diacarperoxide I (**2**) in DMSO.

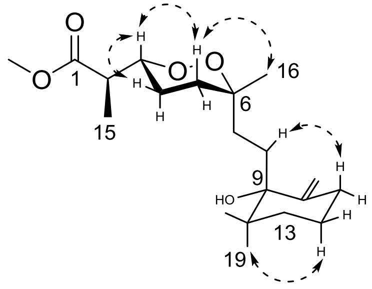

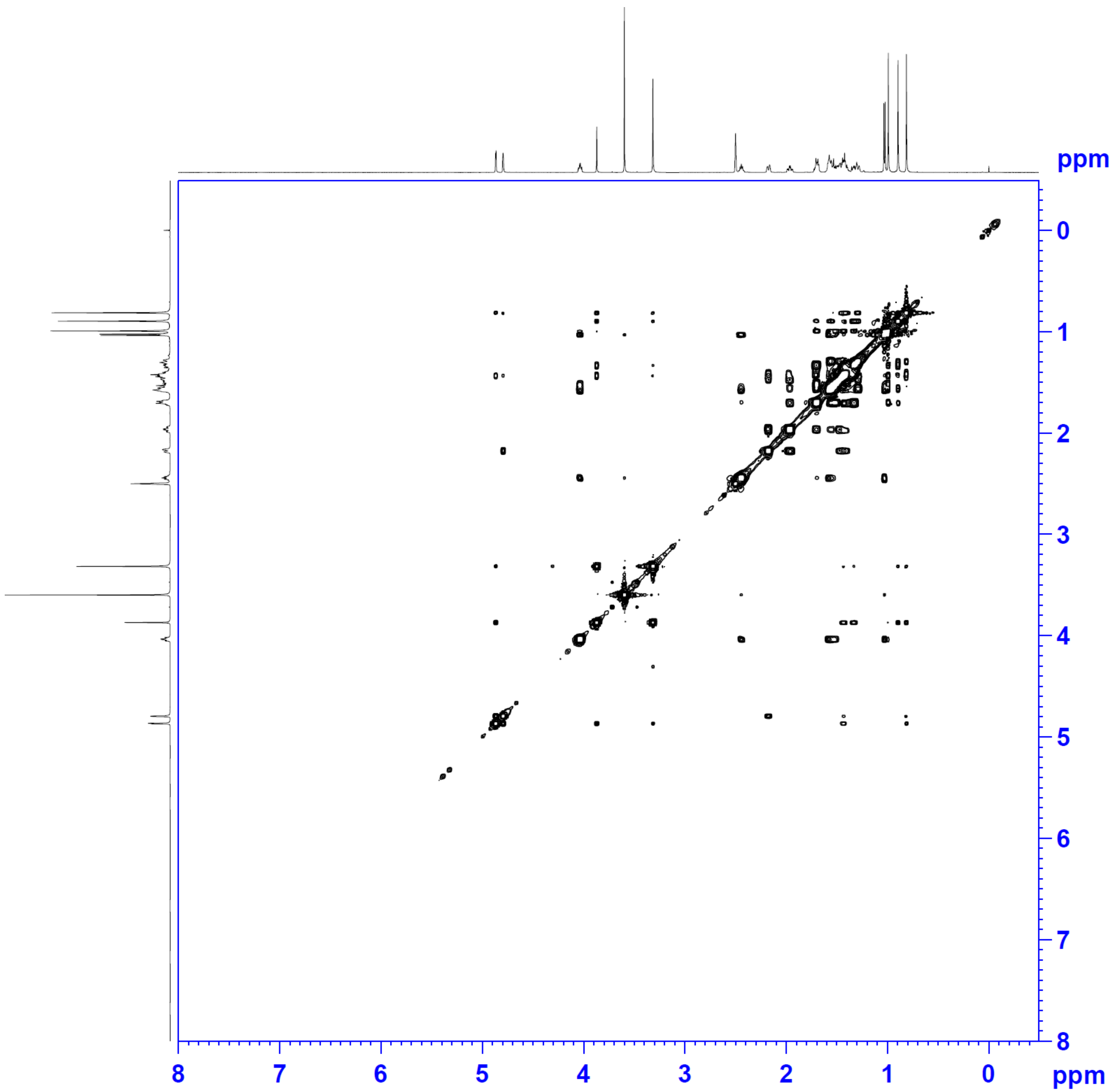

Figure S17. High resolution ESI mass spectrum of diacarperoxide J (**3**).

Mass Spectrum Molecular Formula Report

Analysis Info

Analysis Name D:\Data\20110707\WOL-51.d
Method tune_50-1000_POS_20110328.m
Sample Name WOL-51
Comment

Acquisition Date 7/7/2011 10:53:15 AM
Operator YP
Instrument / Ser# micrOTOF-Q 125

Acquisition Parameter

Source Type	ESI	Ion Polarity	Positive	Set Nebulizer	0.5 Bar
Focus	Not active	Set Capillary	4500 V	Set Dry Heater	180 °C
Scan Begin	50 m/z	Set End Plate Offset	-500 V	Set Dry Gas	4.0 l/min
Scan End	3000 m/z	Set Collision Cell RF	250.0 Vpp	Set Divert Valve	Source

Generate Molecular Formula Parameter

Formula, min.	C20H34Na1O5				
Formula, max.					
Measured m/z	377.23	Tolerance	5 ppm	Charge	1
Check Valence	no	Minimum	0	Maximum	0
Nirogen Rule	no	Electron Configuration	both		
Filter H/C Ratio	no	Minimum	0	Maximum	3
Estimate Carbon	yes				

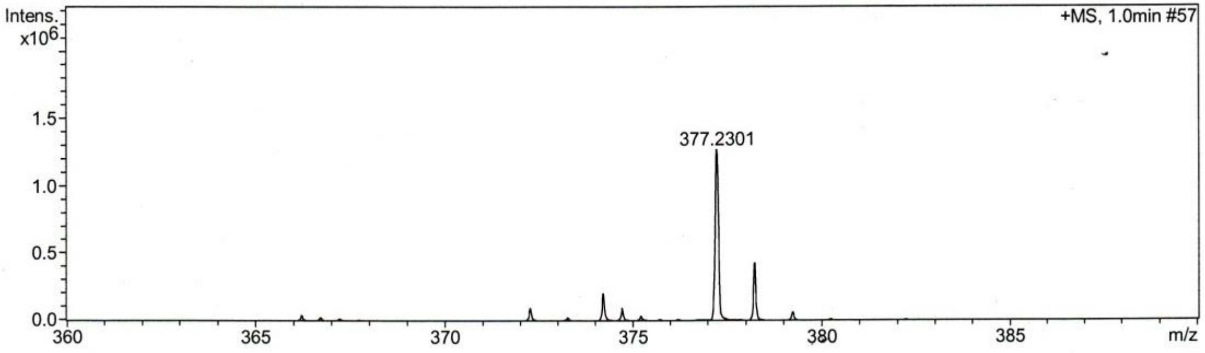

#	m/z	I
1	377.2301	1268128

Sum Formula	Sigma	m/z	Err [ppm]	Mean Err [ppm]	Err [mDa]	rdb	N Rule	e⁻
C 20 H 34 Na 1 O 5	0.130	377.2298	-0.78	-0.78	-0.29	3.50	ok	even

Figure S18. IR spectrum of diacarperoxide J (**3**).

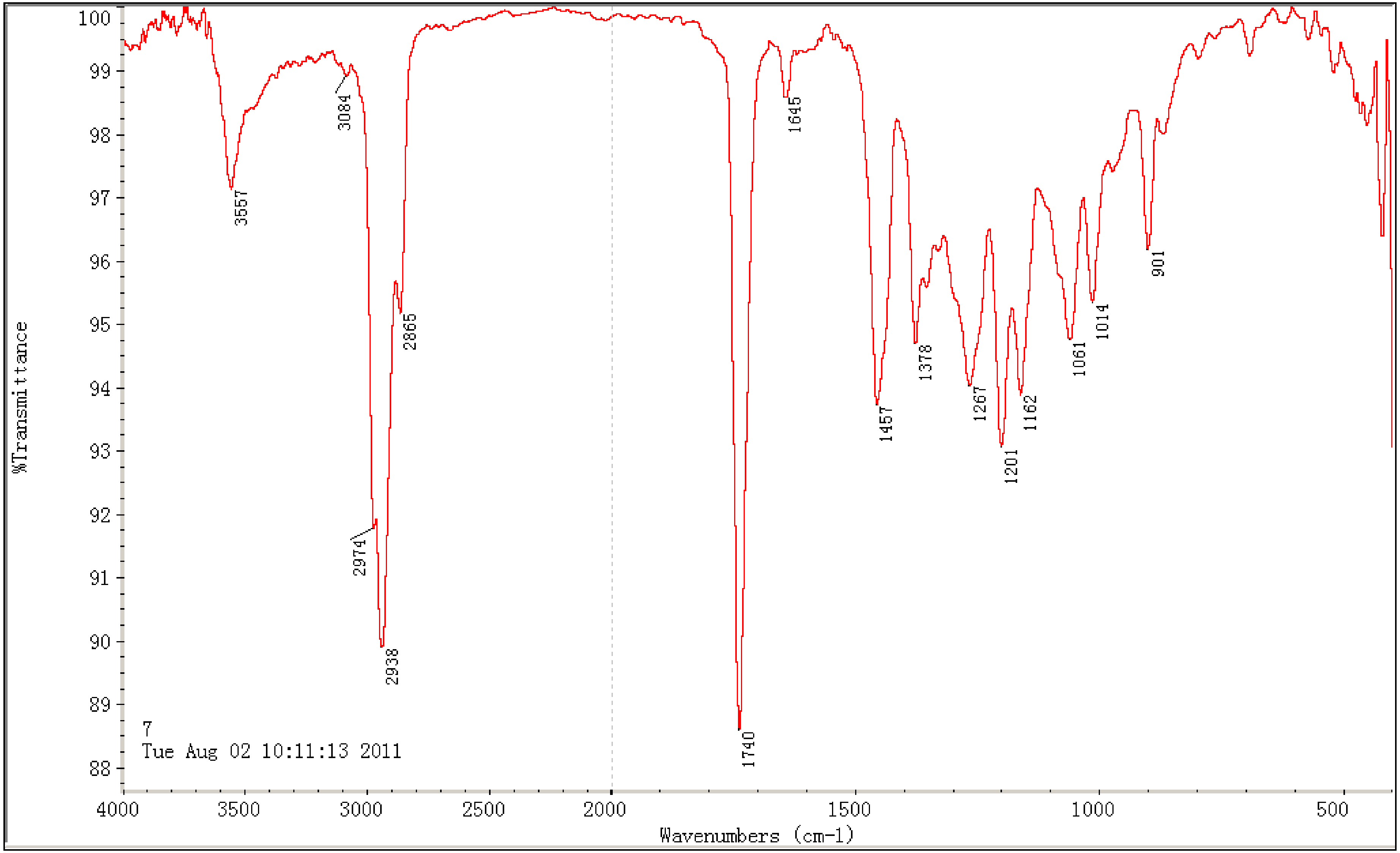

Figure S19. ^{1}H NMR spectrum of diacarperoxide J (**3**) in $CDCl_3$.

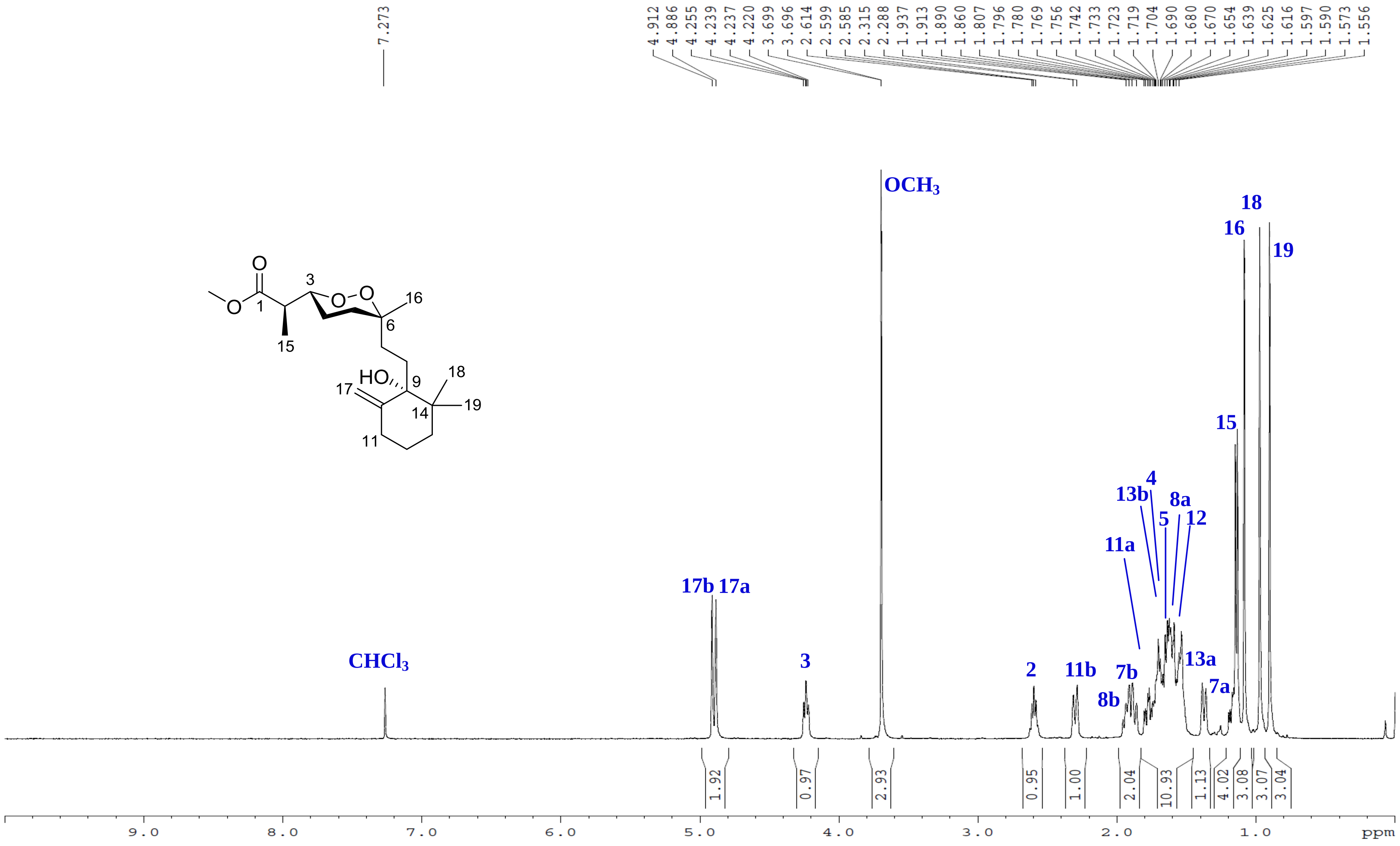

Figure S20. ^{13}C NMR spectrum of diacarperoxide J (**3**) in $CDCl_3$.

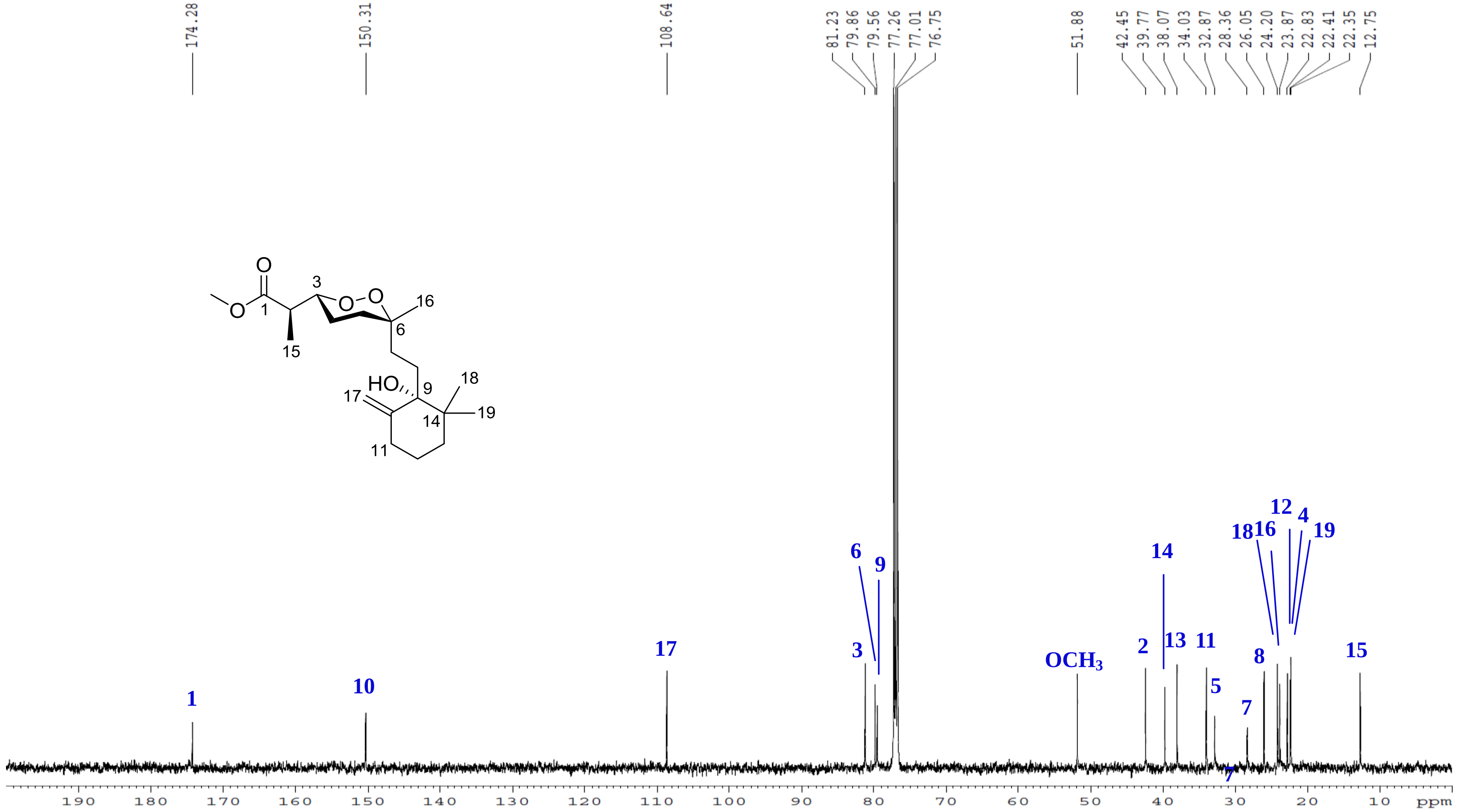

Figure S21. ^{1}H-^{1}H COSY spectrum of diacarperoxide J (**3**) in $CDCl_3$.

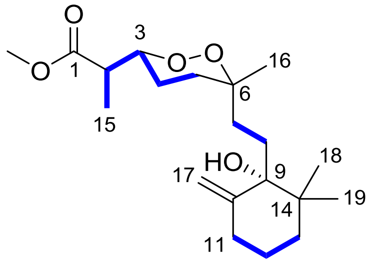

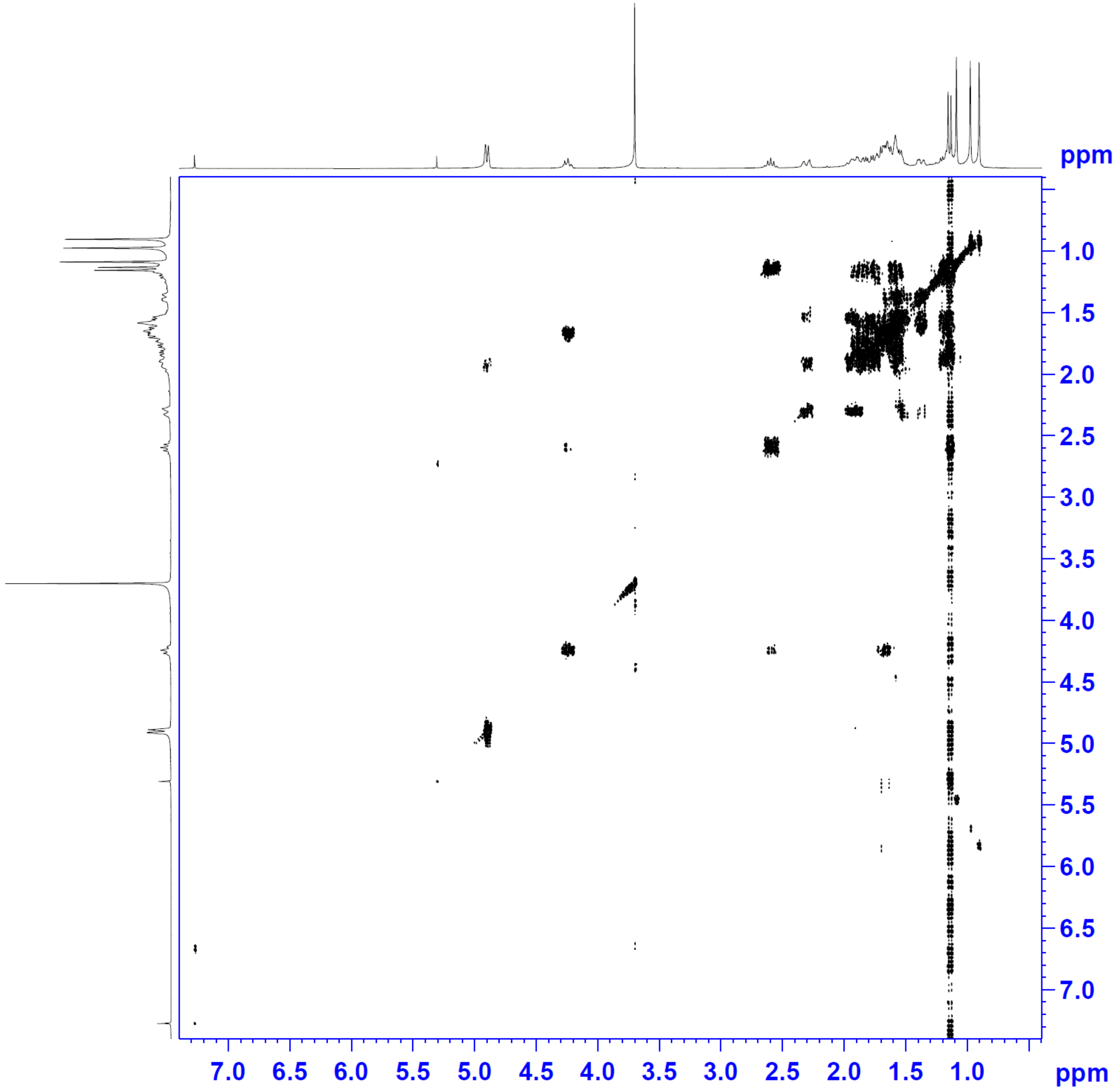

Figure S22. HSQC spectrum of diacarperoxide J (**3**) in $CDCl_3$.

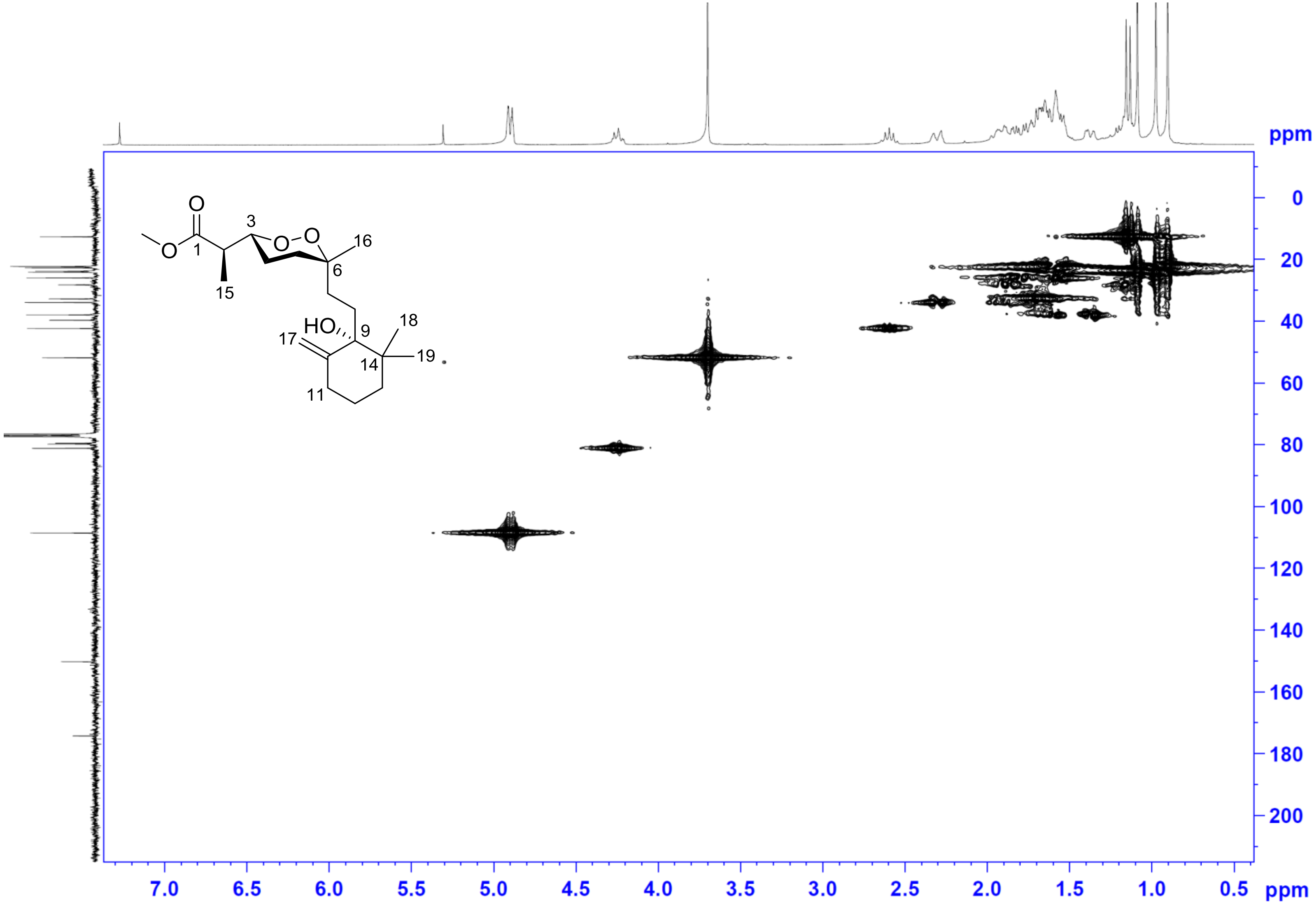

Figure S23. HMBC spectrum of diacarperoxide J (**3**) in $CDCl_3$.

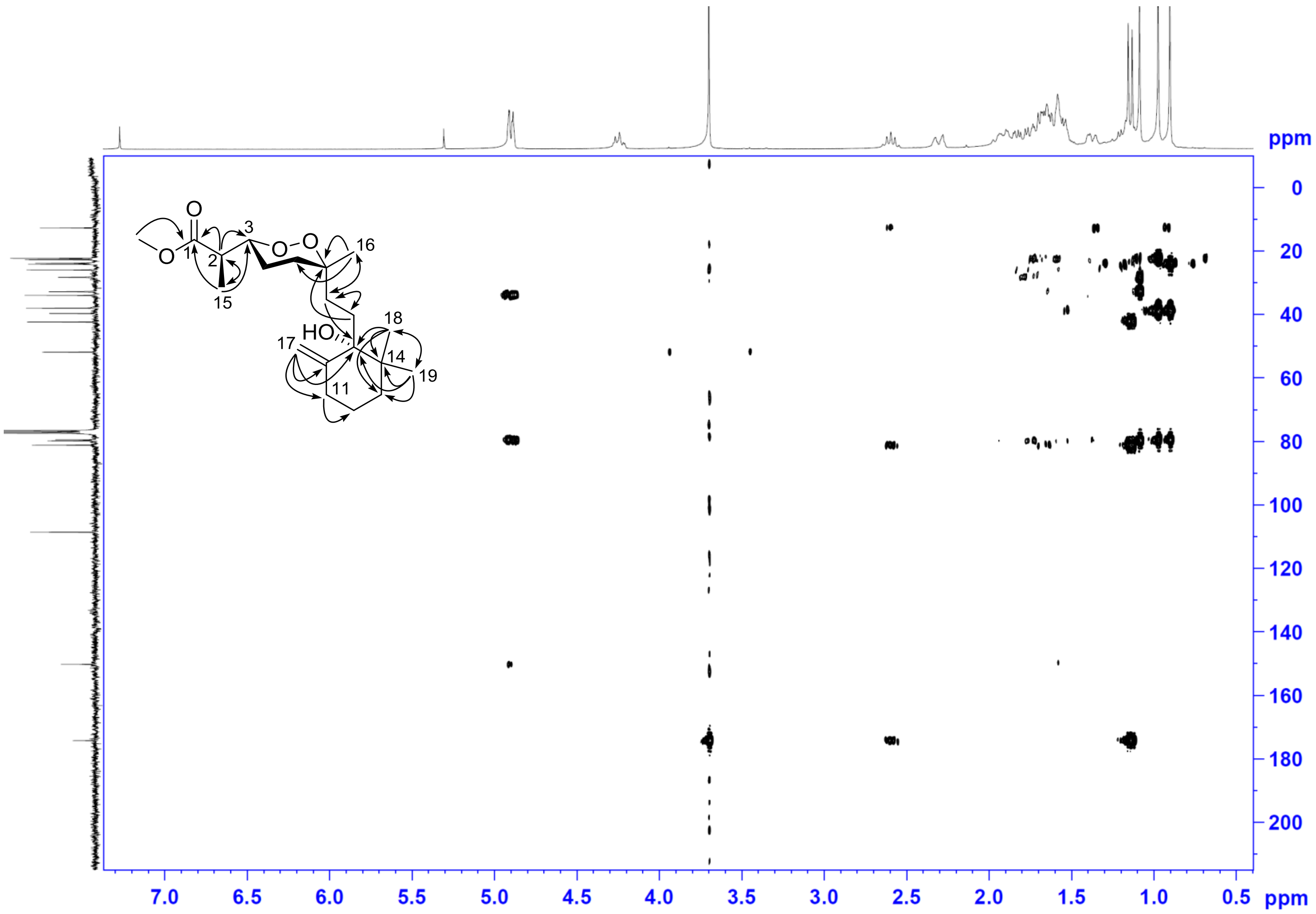

Figure S24. NOESY spectrum of diacarperoxide J (**3**) in DMSO.

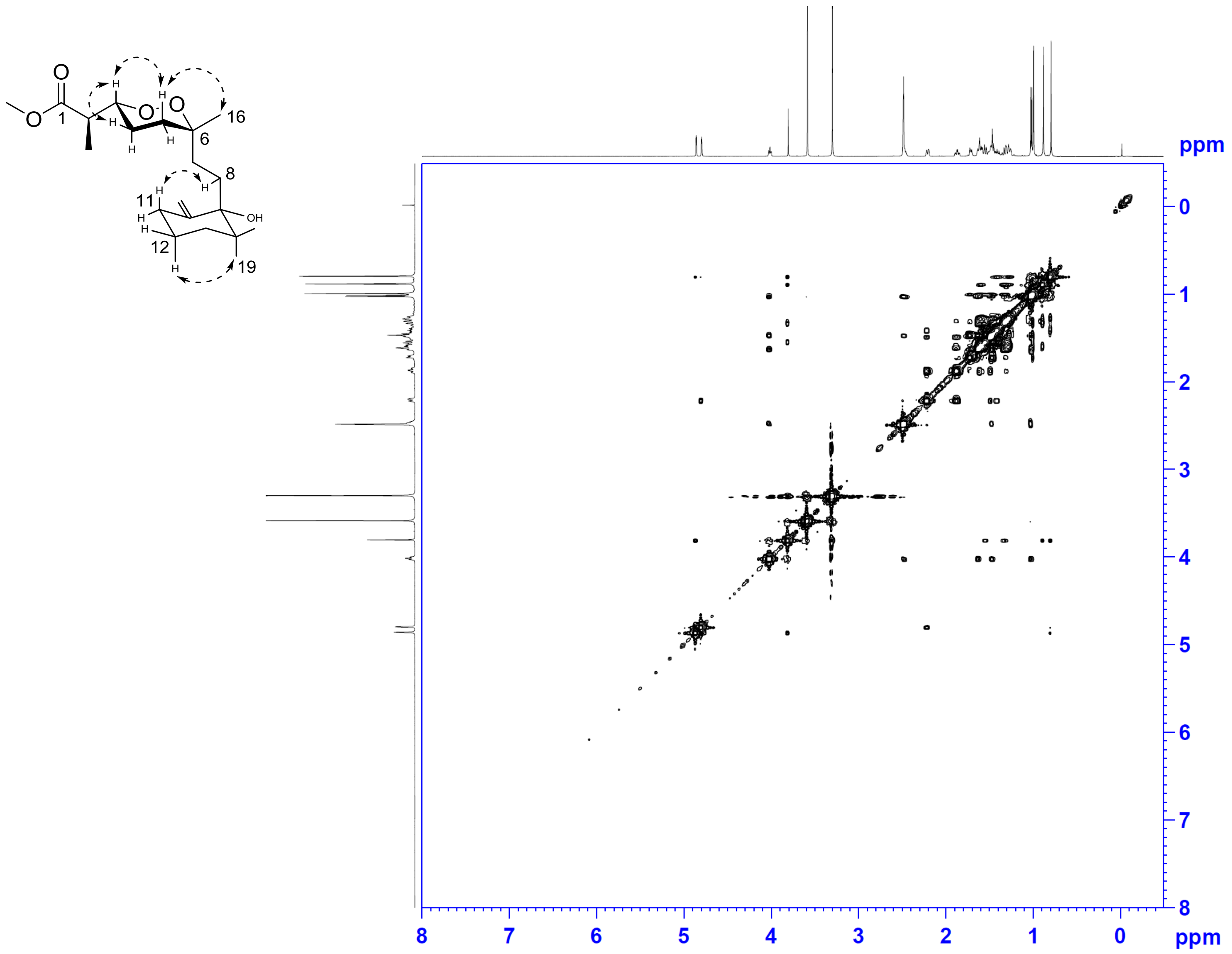

Figure S25. High resolution ESI mass spectrum of diacarperoxide K (**4**).

Mass Spectrum Molecular Formula Report

Analysis Info

Analysis Name	D:\Data\20110707\2COM.d	Acquisition Date	7/7/2011 10:56:47 AM
Method	tune_50-1000_POS_20110328.m	Operator	YP
Sample Name	2COM	Instrument / Ser#	micrOTOF-Q 125
Comment			

Acquisition Parameter

Source Type	ESI	Ion Polarity	Positive	Set Nebulizer	0.5 Bar
Focus	Not active	Set Capillary	4500 V	Set Dry Heater	180 °C
Scan Begin	50 m/z	Set End Plate Offset	-500 V	Set Dry Gas	4.0 l/min
Scan End	3000 m/z	Set Collision Cell RF	80.0 Vpp	Set Divert Valve	Source

Generate Molecular Formula Parameter

Formula, min.	C20H34Na1O5				
Formula, max.					
Measured m/z	377.23	Tolerance	5 ppm	Charge	1
Check Valence	no	Minimum	0	Maximum	0
Nirogen Rule	no	Electron Configuration	both		
Filter H/C Ratio	no	Minimum	0	Maximum	3
Estimate Carbon	yes				

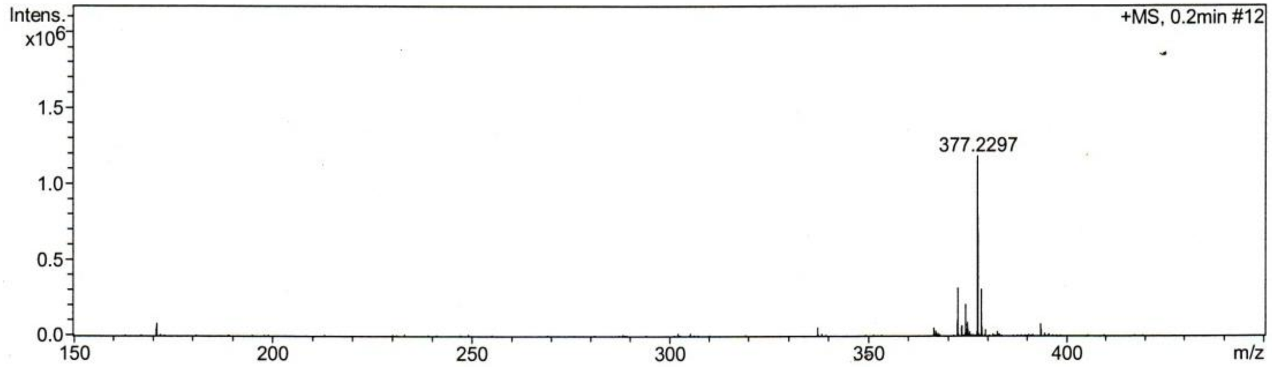

#	m/z	I
1	377.2297	1179875

Sum Formula	Sigma	m/z	Err [ppm]	Mean Err [ppm]	Err [mDa]	rdb	N Rule	e⁻
C 20 H 34 Na 1 O 5	0.130	377.2298	0.34	0.34	0.13	3.50	ok	even

Figure S26. IR spectrum of diacarperoxide K (**4**).

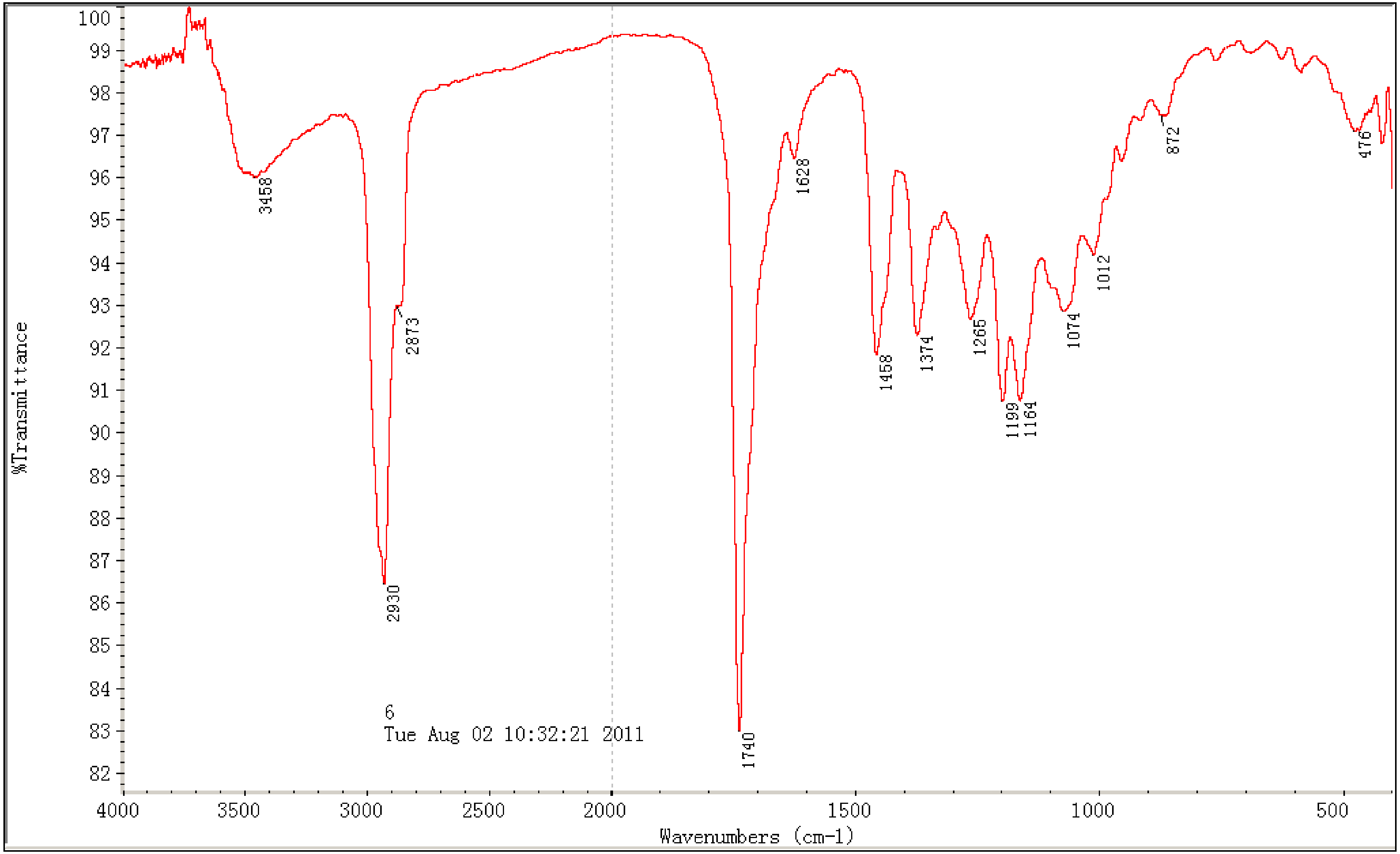

Figure S27. ^{1}H NMR spectrum of diacarperoxide K (**4**) in $CDCl_3$.

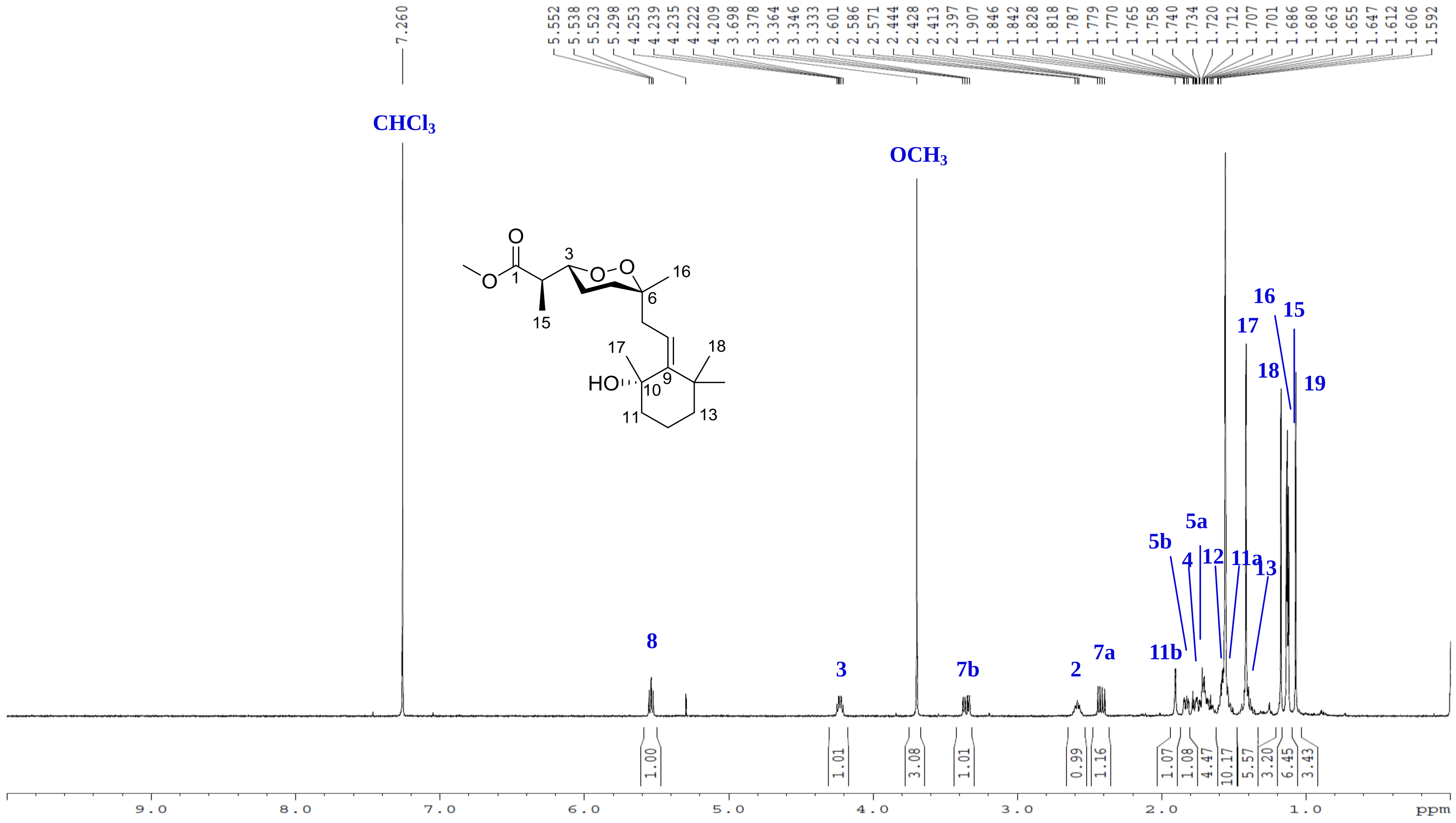

Figure S28. ^{13}C NMR spectrum of diacarperoxide K (**4**) in $CDCl_3$.

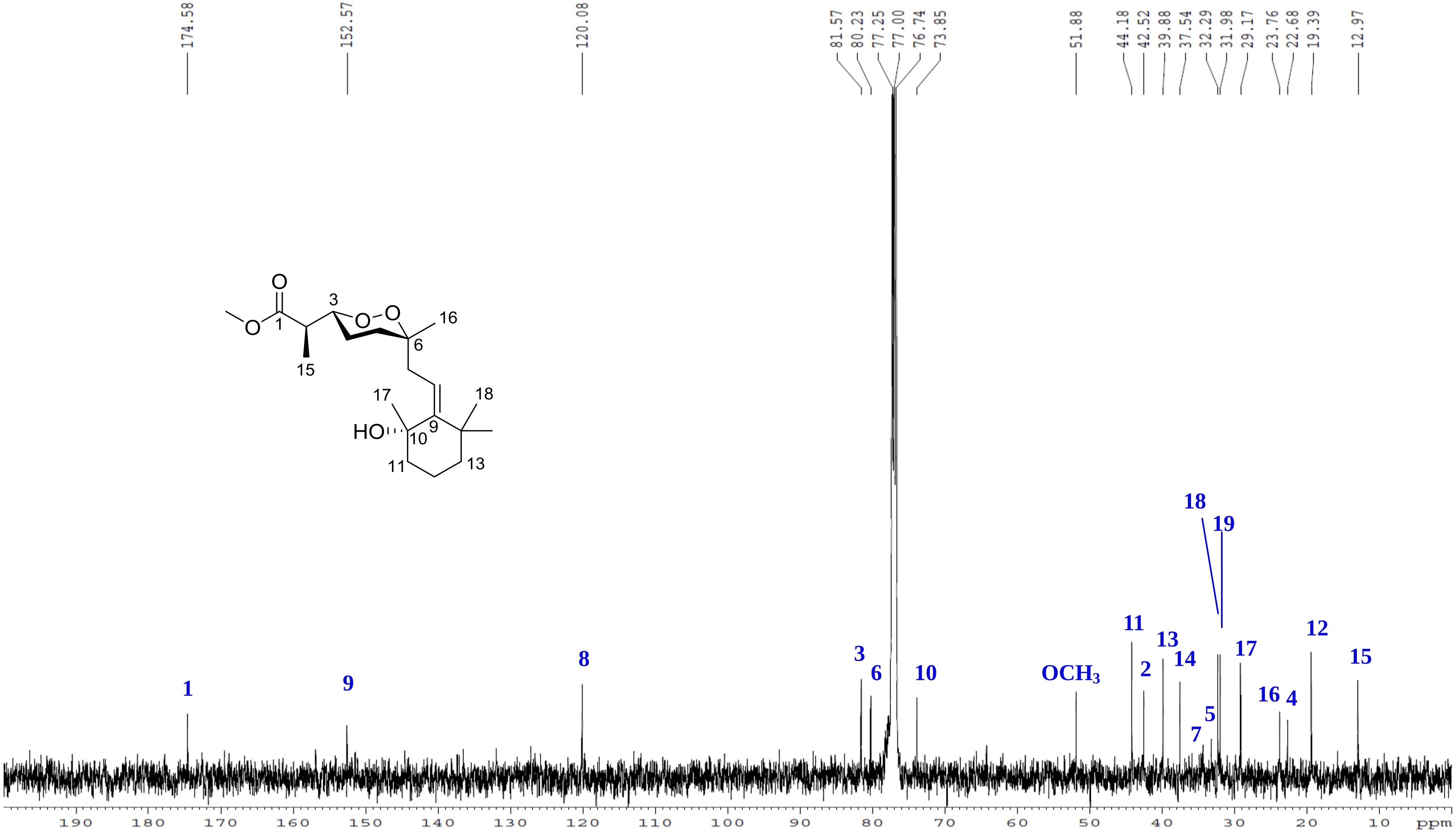

Figure S29. ^{1}H-^{1}H COSY spectrum of diacarperoxide K (**4**) in $CDCl_3$.

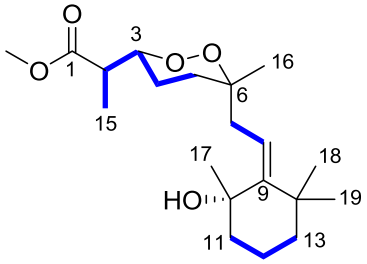

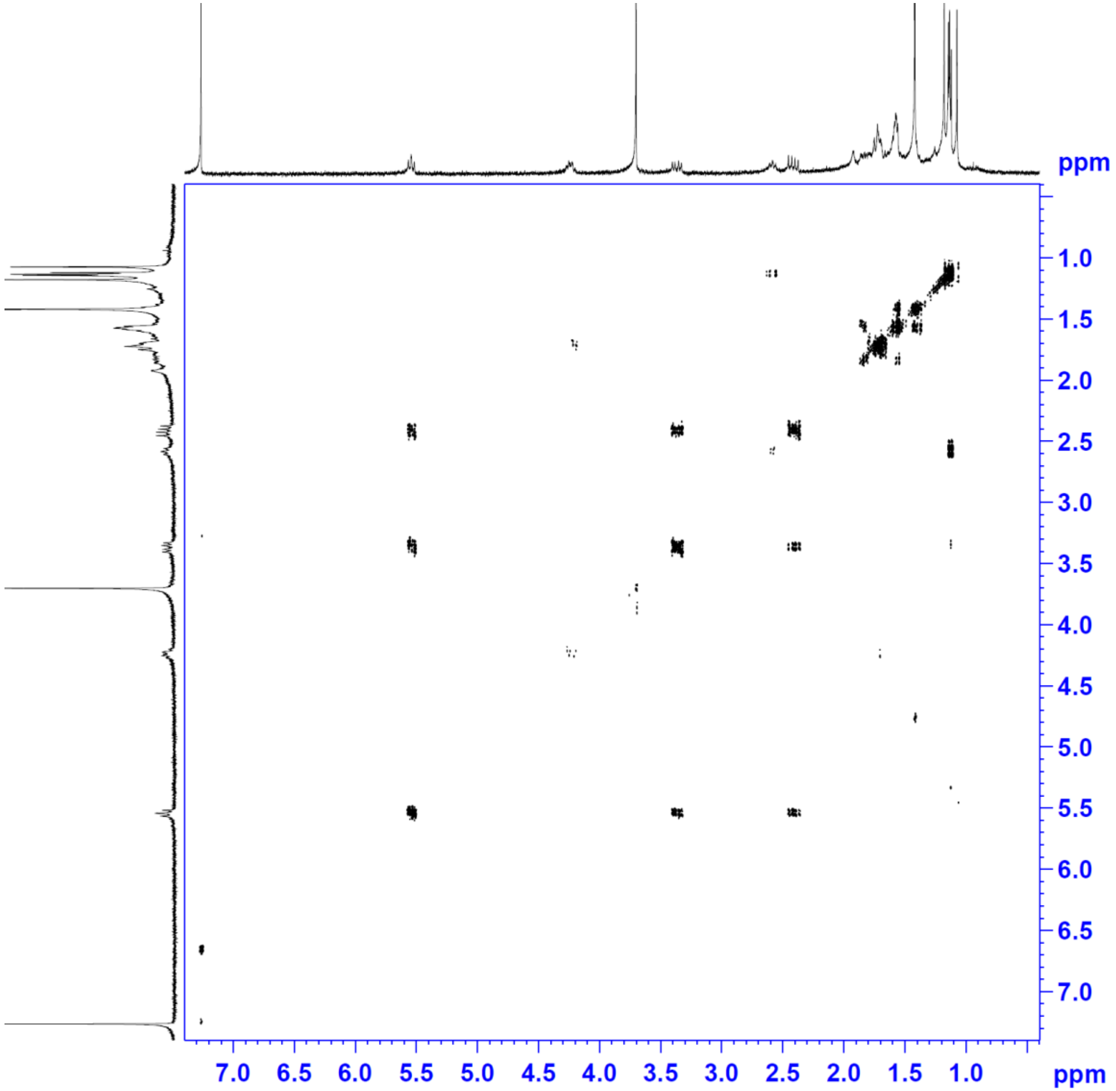

Figure S30. HSQC spectrum of diacarperoxide K (**4**) in $CDCl_3$.

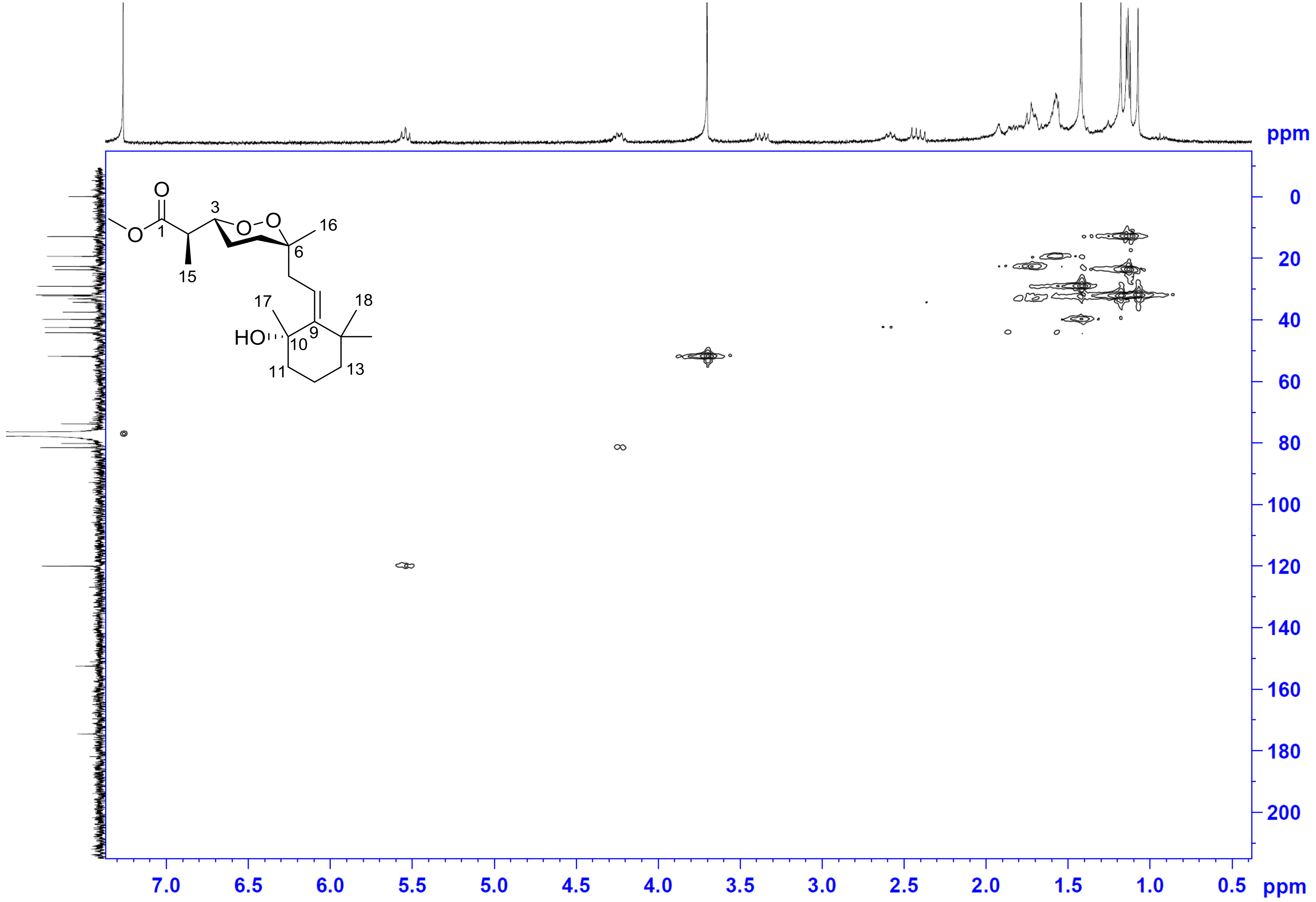

Figure S31. HMBC spectrum of diacarperoxide K (**4**) in $CDCl_3$.

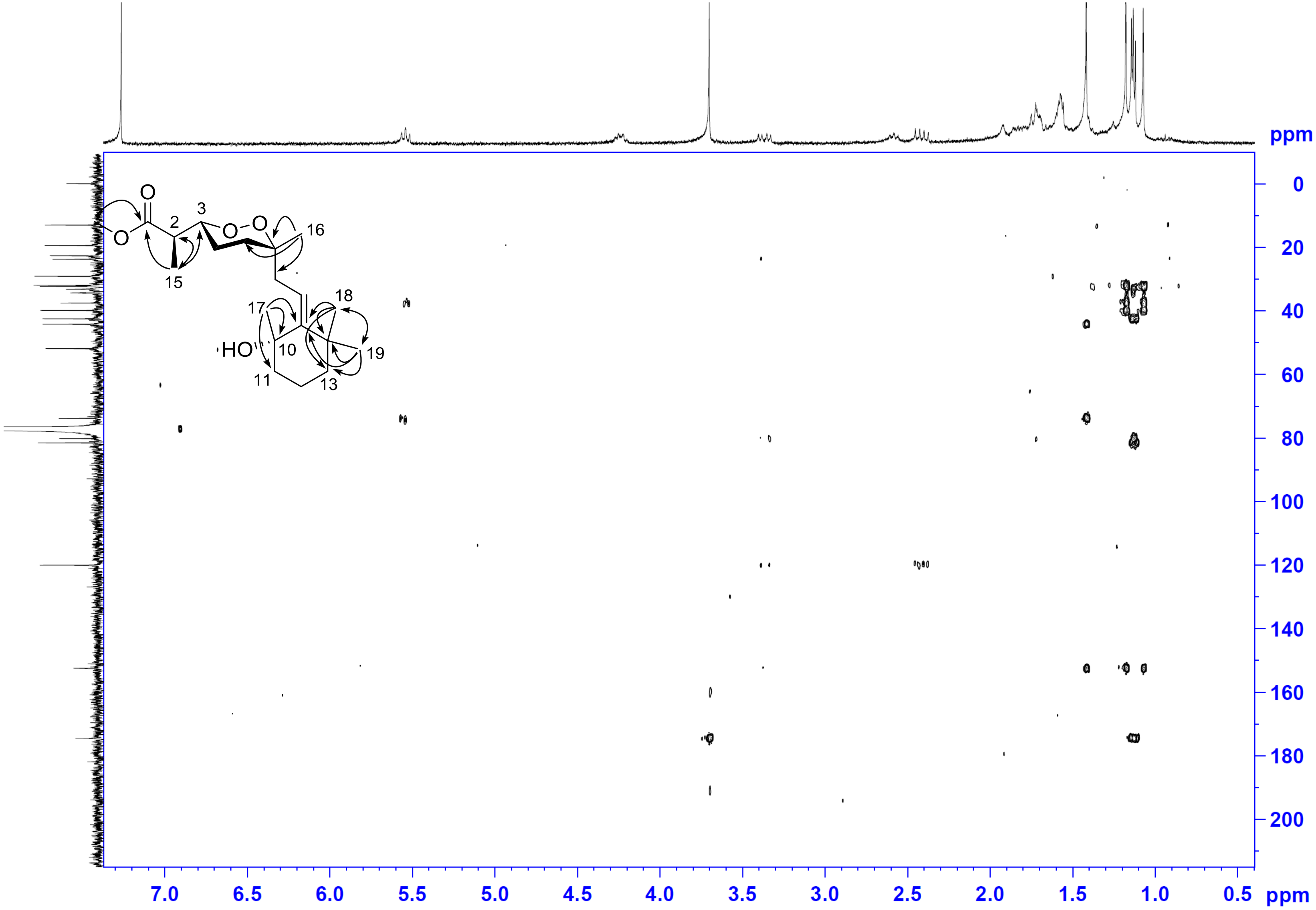

Figure S32. NOESY spectrum of diacarperoxide K (**4**) in $CDCl_3$.

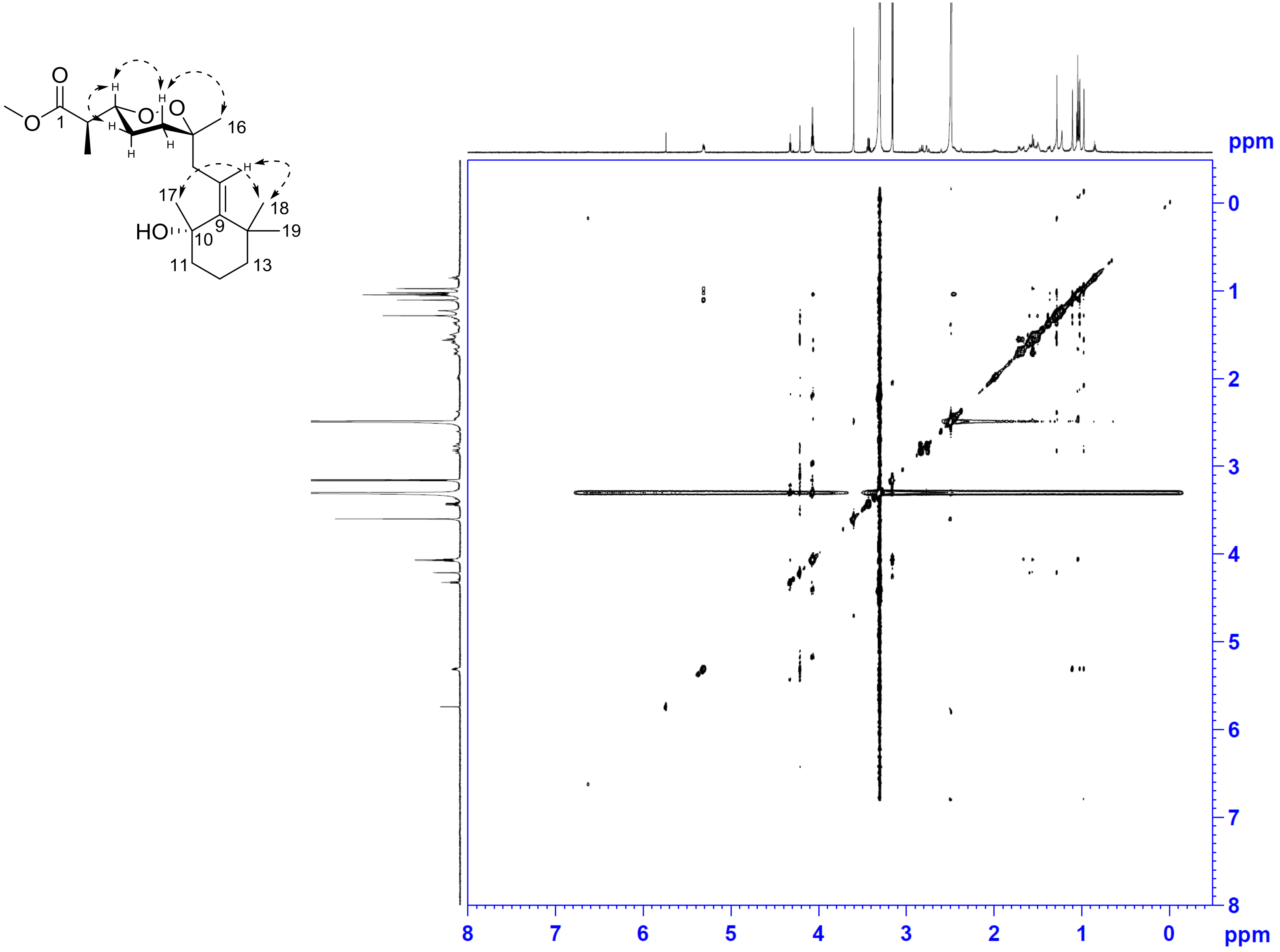

Figure S33. High resolution ESI mass spectrum of diacarperoxide L (**5**).

Mass Spectrum Molecular Formula Report

Analysis Info

Analysis Name	D:\Data\20110707\WOL-13.d	Acquisition Date	7/7/2011 11:03:06 AM
Method	tune_50-1000_POS_20110328.m	Operator	YP
Sample Name	2COM	Instrument / Ser#	micrOTOF-Q 125
Comment			

Acquisition Parameter

Source Type	ESI	Ion Polarity	Positive	Set Nebulizer	0.5 Bar
Focus	Not active	Set Capillary	4500 V	Set Dry Heater	180 °C
Scan Begin	50 m/z	Set End Plate Offset	-500 V	Set Dry Gas	4.0 l/min
Scan End	3000 m/z	Set Collision Cell RF	70.0 Vpp	Set Divert Valve	Source

Generate Molecular Formula Parameter

Formula, min.	C20H34Na1O5				
Formula, max.					
Measured m/z	377.23	Tolerance	5 ppm	Charge	1
Check Valence	no	Minimum	0	Maximum	0
Nirogen Rule	no	Electron Configuration	both		
Filter H/C Ratio	no	Minimum	0	Maximum	3
Estimate Carbon	yes				

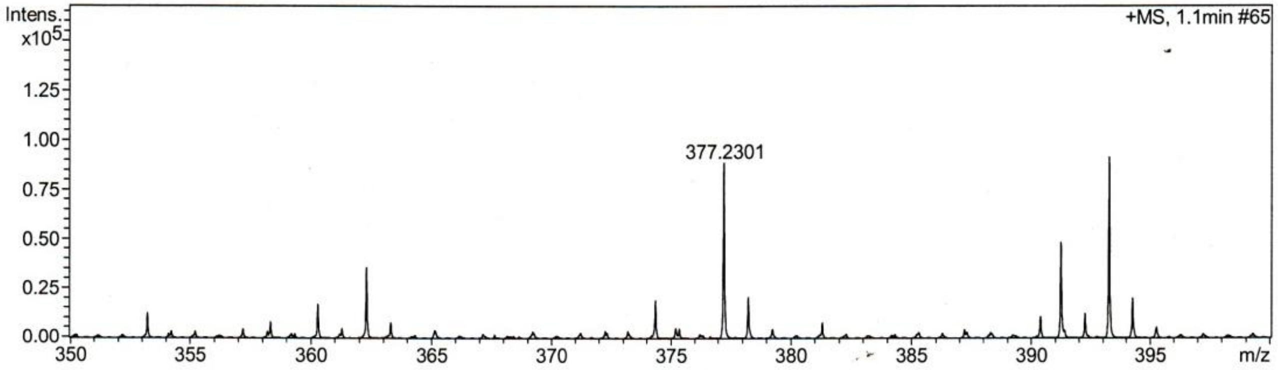

#	m/z	I
1	377.2301	88491

Sum Formula	Sigma	m/z	Err [ppm]	Mean Err [ppm]	Err [mDa]	rdb	N Rule	e⁻
C 20 H 34 Na 1 O 5	0.130	377.2298	-0.72	-0.72	-0.27	3.50	ok	even

Figure S34. IR spectrum of diacarperoxide L (**5**).

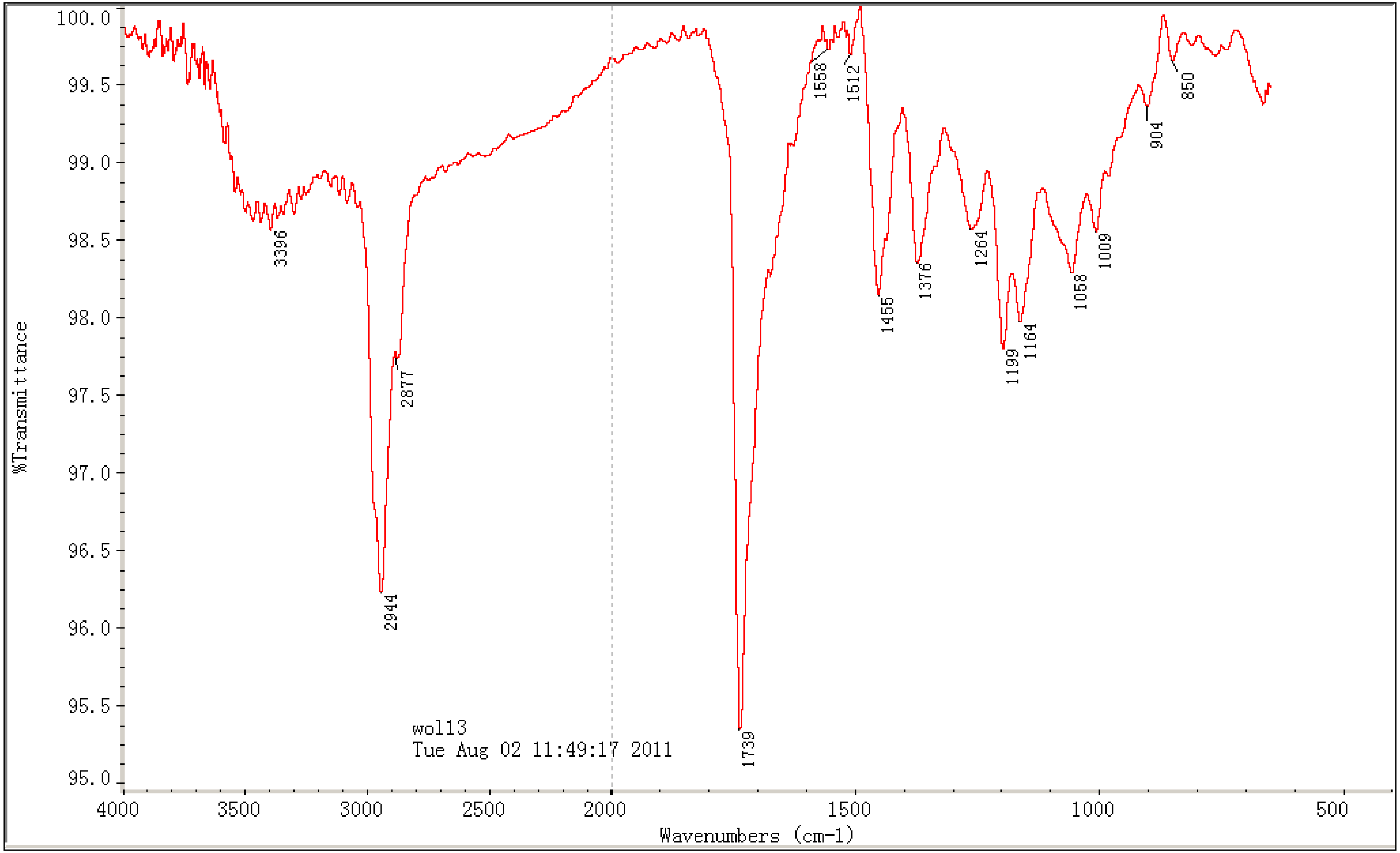

Figure S35. ¹H NMR spectrum of diacarperoxide L (**5**) in $CDCl_3$.

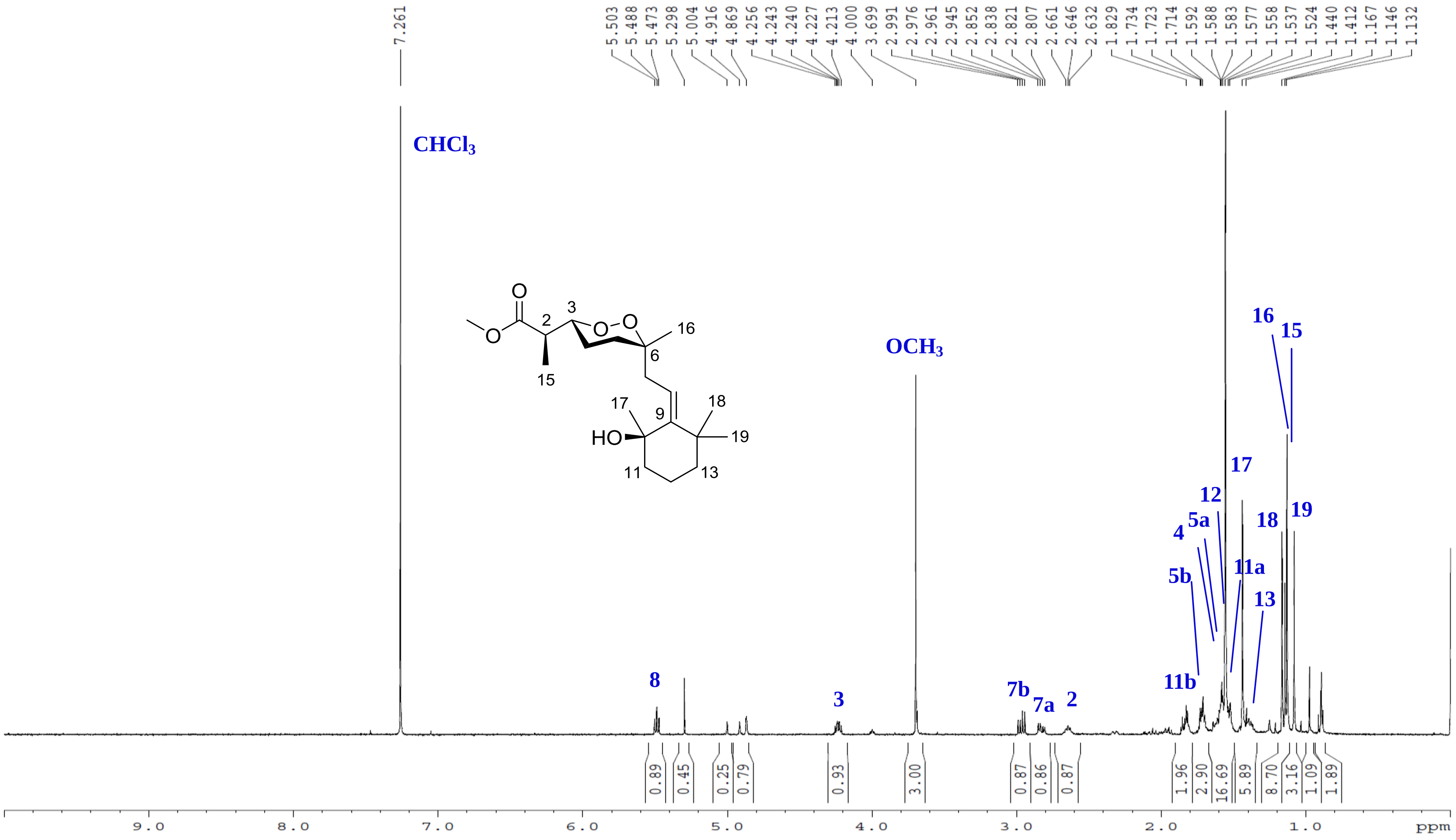

Figure S36. ^{13}C NMR spectrum of diacarperoxide L (**5**) in $CDCl_3$.

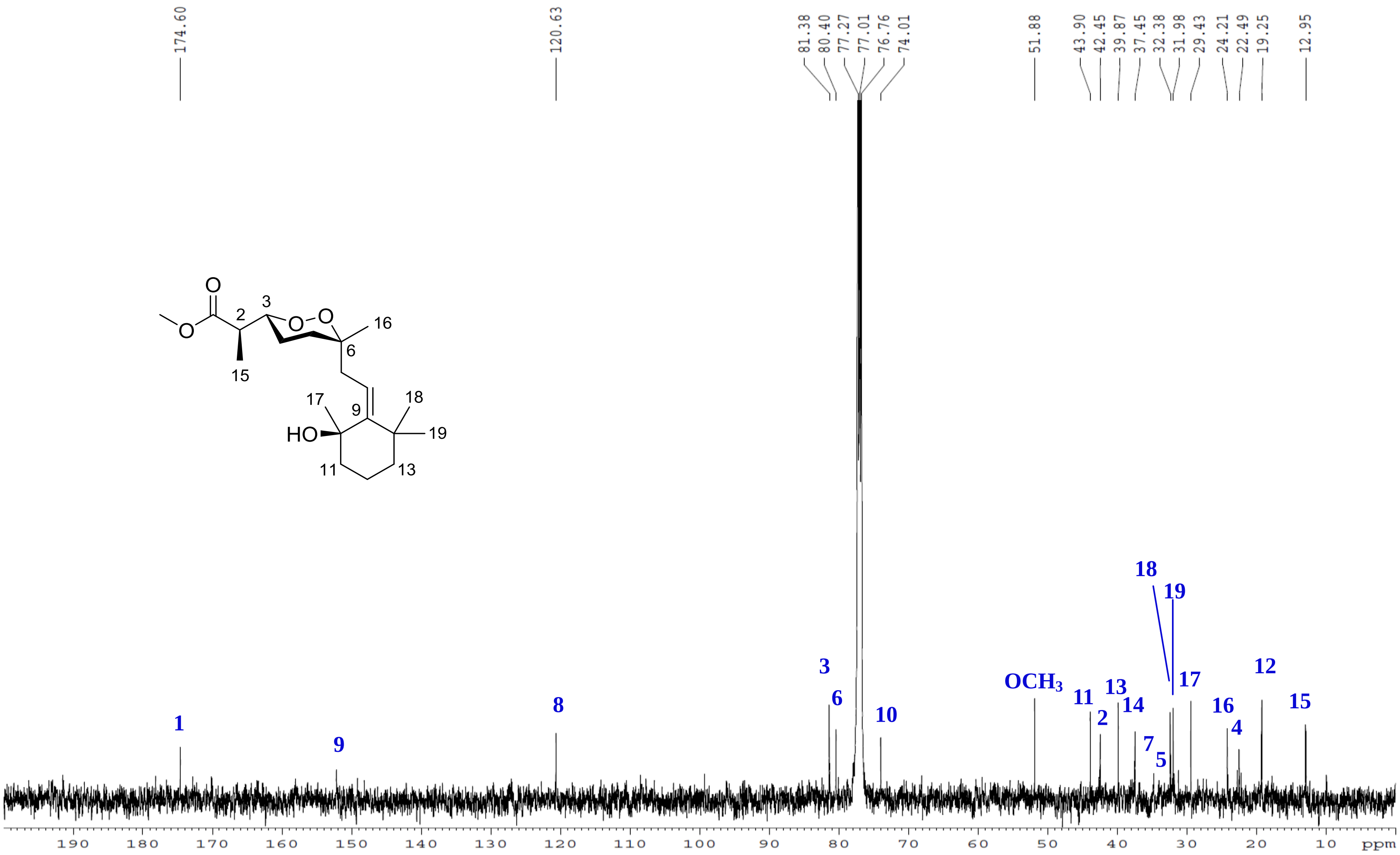

Figure S37. ^{1}H-^{1}H COSY spectrum of diacarperoxide L (**5**) in $CDCl_3$.

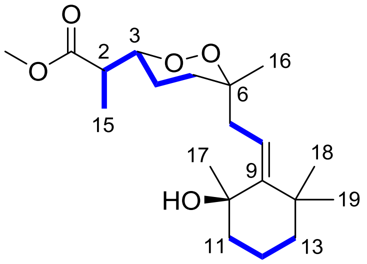

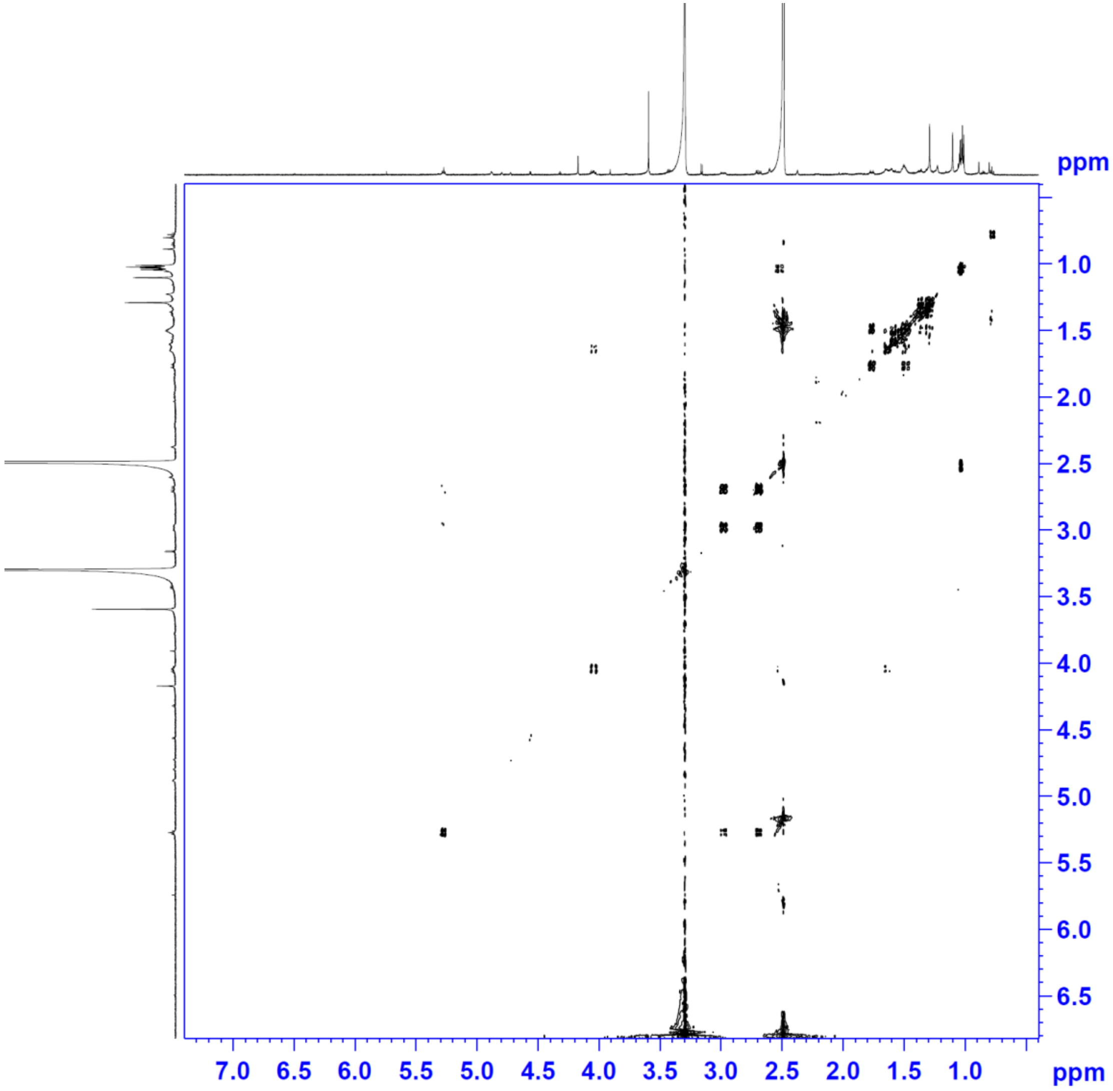

Figure S38. HSQC spectrum of diacarperoxide L (**5**) in $CDCl_3$.

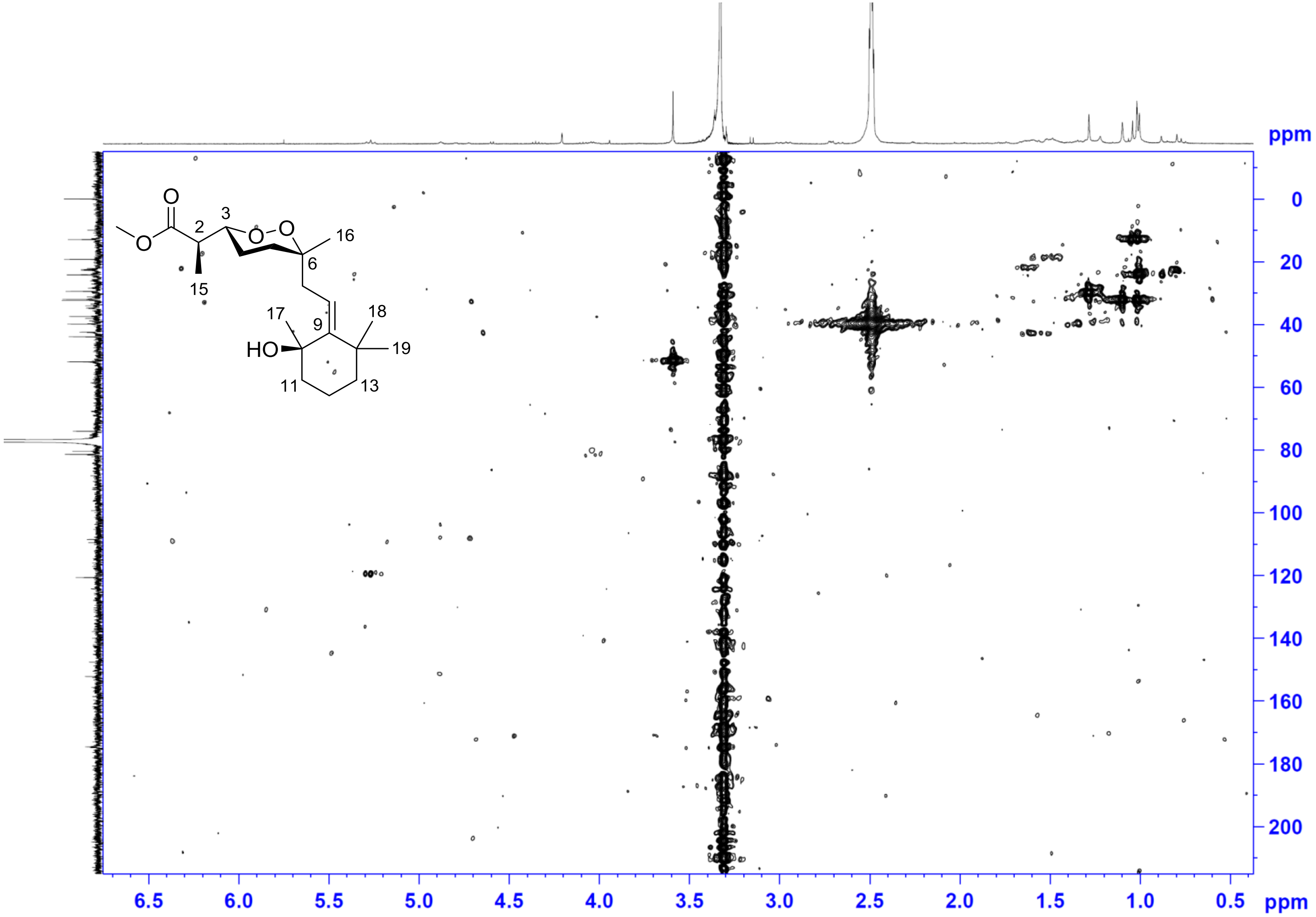

Figure S39. HMBC spectrum of diacarperoxide L (**5**) in $CDCl_3$.

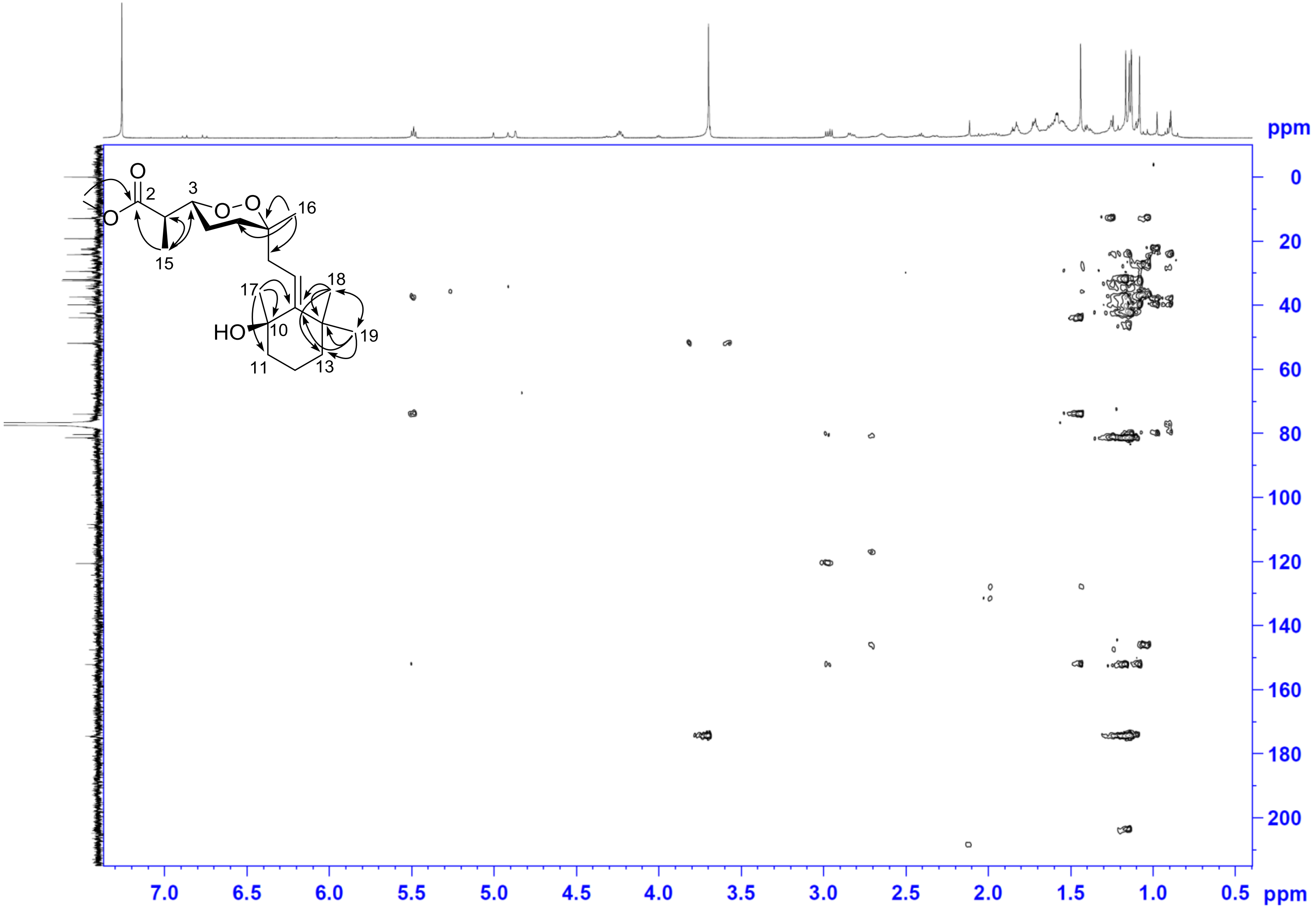

Figure S40. NOESY spectrum of diacarperoxide L (**5**) in $CDCl_3$.

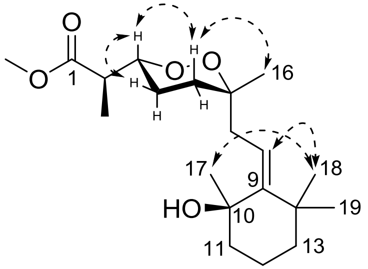

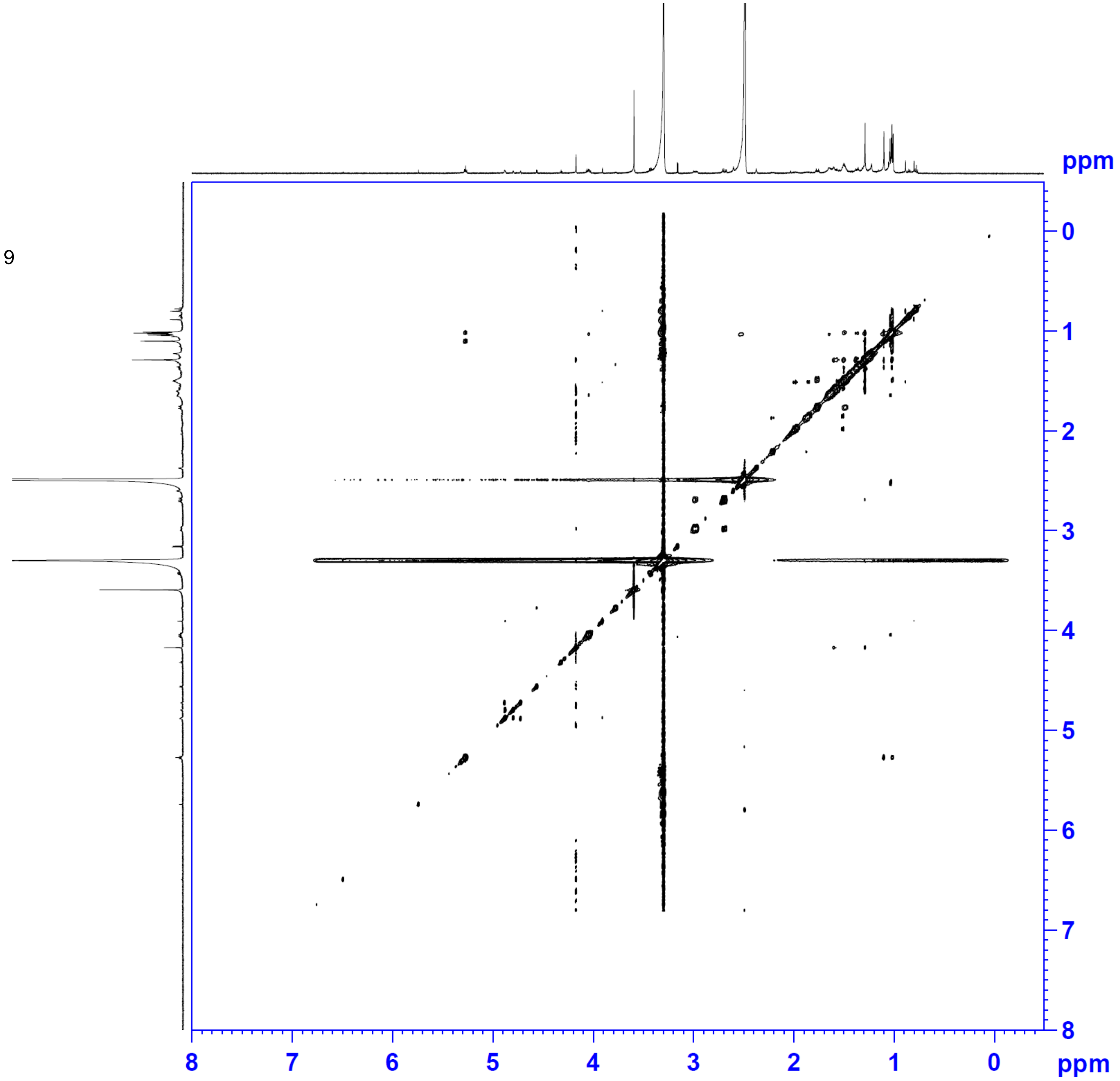

Figure S41. High resolution ESI mass spectrum of diacardiol B (**6**).

Mass Spectrum Molecular Formula Report

Analysis Info

Analysis Name	D:\Data\20110707\WOL-80.d	Acquisition Date	7/7/2011 10:14:46 AM
Method	tune_50-1000_POS_20110328.m	Operator	YP
Sample Name	WOL-80	Instrument / Ser#	micrOTOF-Q 125
Comment			

Acquisition Parameter

Source Type	ESI	Ion Polarity	Positive	Set Nebulizer	0.5 Bar
Focus	Not active	Set Capillary	4500 V	Set Dry Heater	180 °C
Scan Begin	50 m/z	Set End Plate Offset	-500 V	Set Dry Gas	4.0 l/min
Scan End	3000 m/z	Set Collision Cell RF	250.0 Vpp	Set Divert Valve	Source

Generate Molecular Formula Parameter

Formula, min.	C20H36Na1O4				
Formula, max.					
Measured m/z	363.251	Tolerance	5 ppm	Charge	1
Check Valence	no	Minimum	0	Maximum	0
Nirogen Rule	no	Electron Configuration	both		
Filter H/C Ratio	no	Minimum	0	Maximum	3
Estimate Carbon	yes				

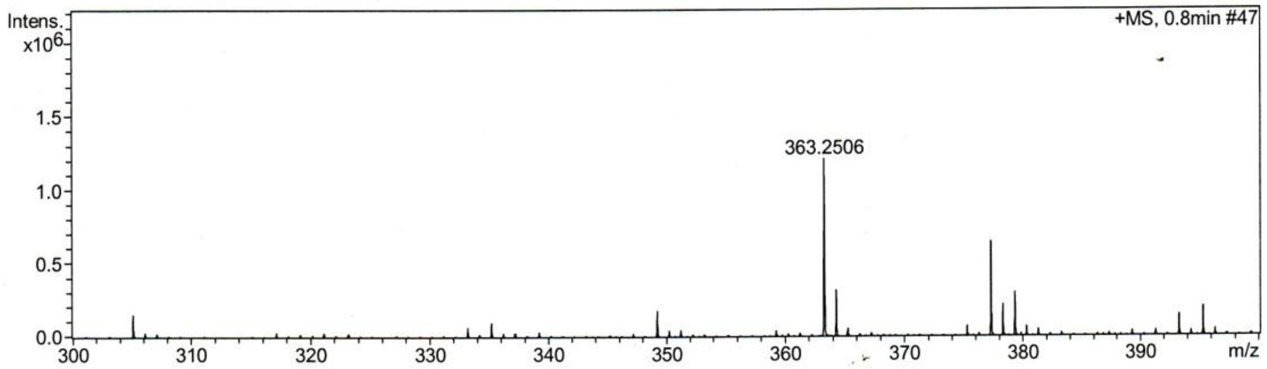

#	m/z	I
1	363.2506	1206064

Sum Formula	Sigma	m/z	Err [ppm]	Mean Err [ppm]	Err [mDa]	rdb	N Rule	e⁻
C 20 H 36 Na 1 O 4	0.129	363.2506	-0.17	-0.17	-0.06	2.50	ok	even

Figure S42. IR spectrum of diacardiol B (**6**).

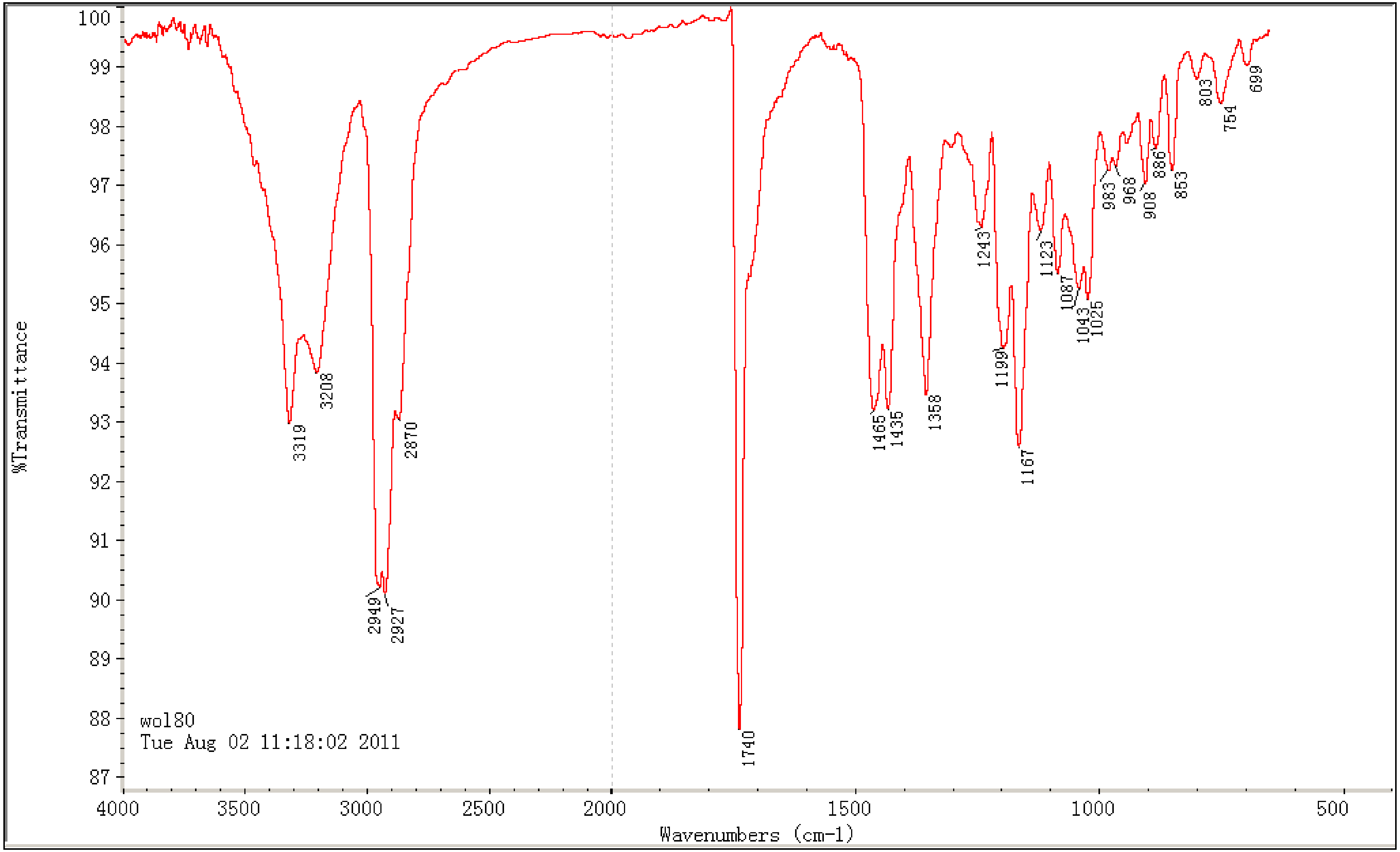

Figure S43. ^{1}H NMR spectrum of diacardiol B (**6**) in $CDCl_3$.

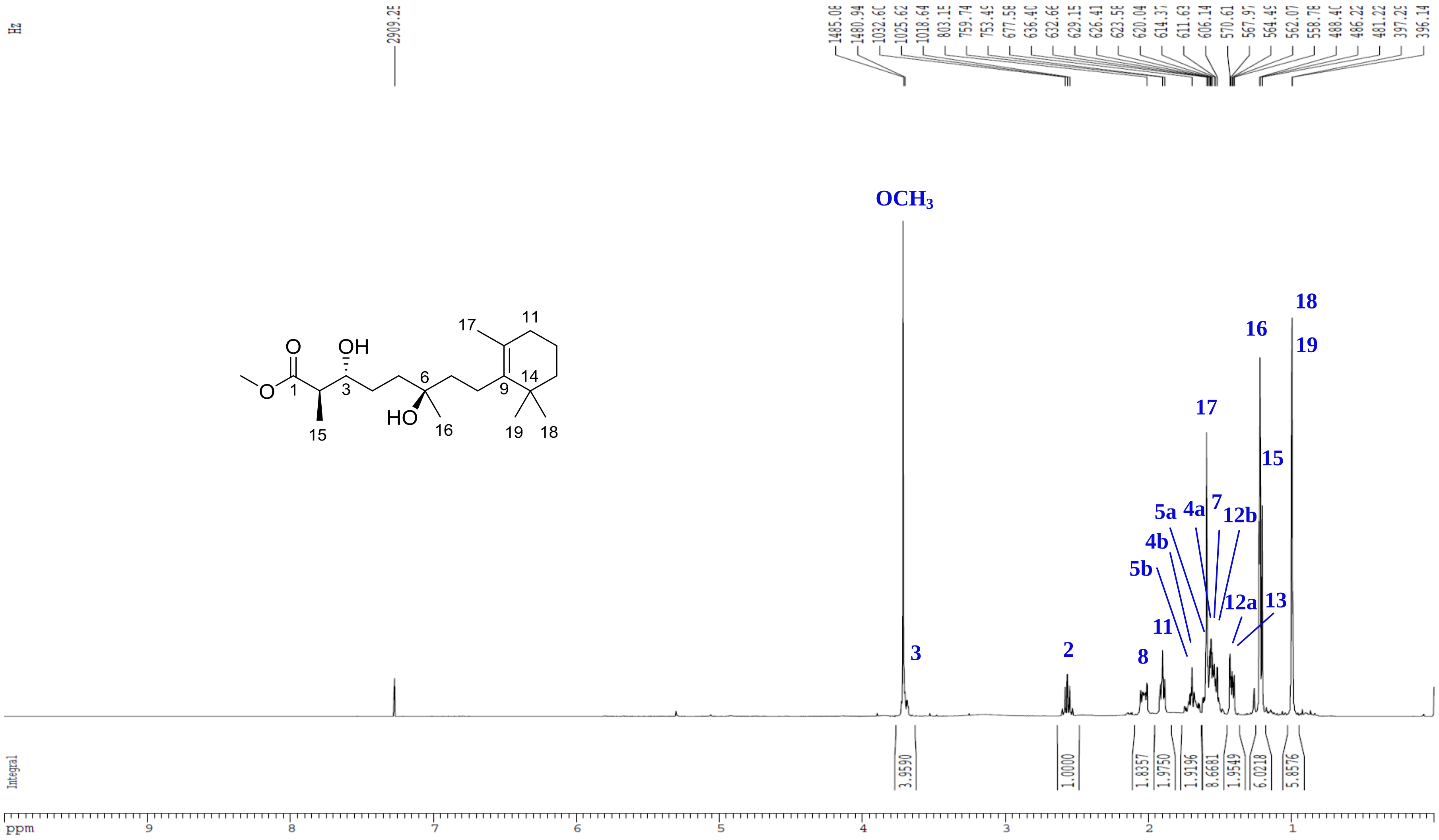

Figure S44. ^{13}C NMR spectrum of diacardiol B (**6**) in $CDCl_3$.

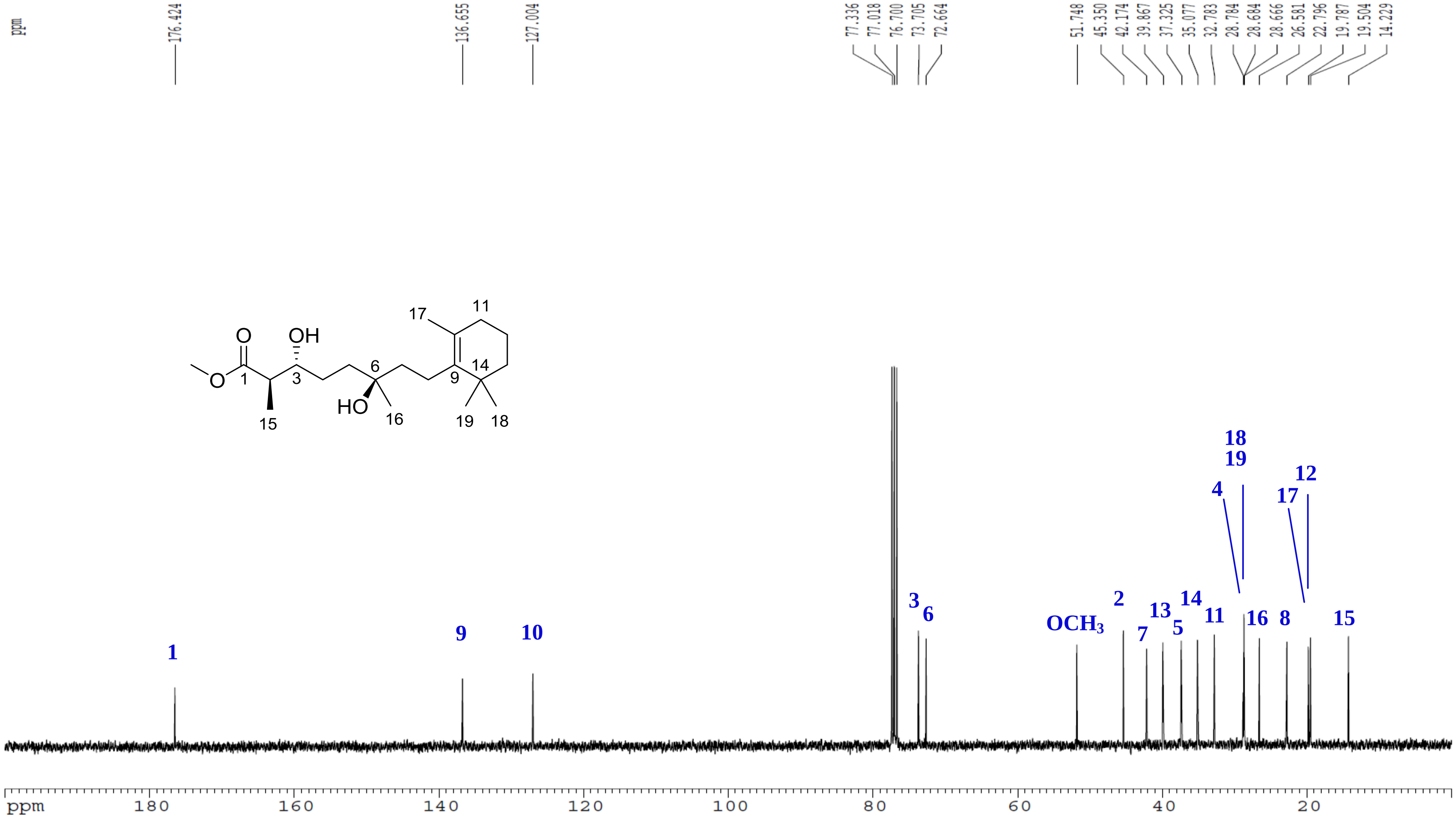

Figure S45. ^{1}H-^{1}H COSY spectrum of diacardiol B (**6**) in $CDCl_3$.

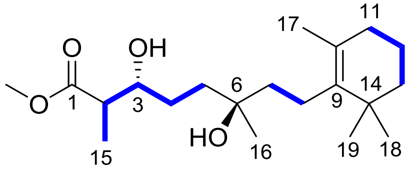

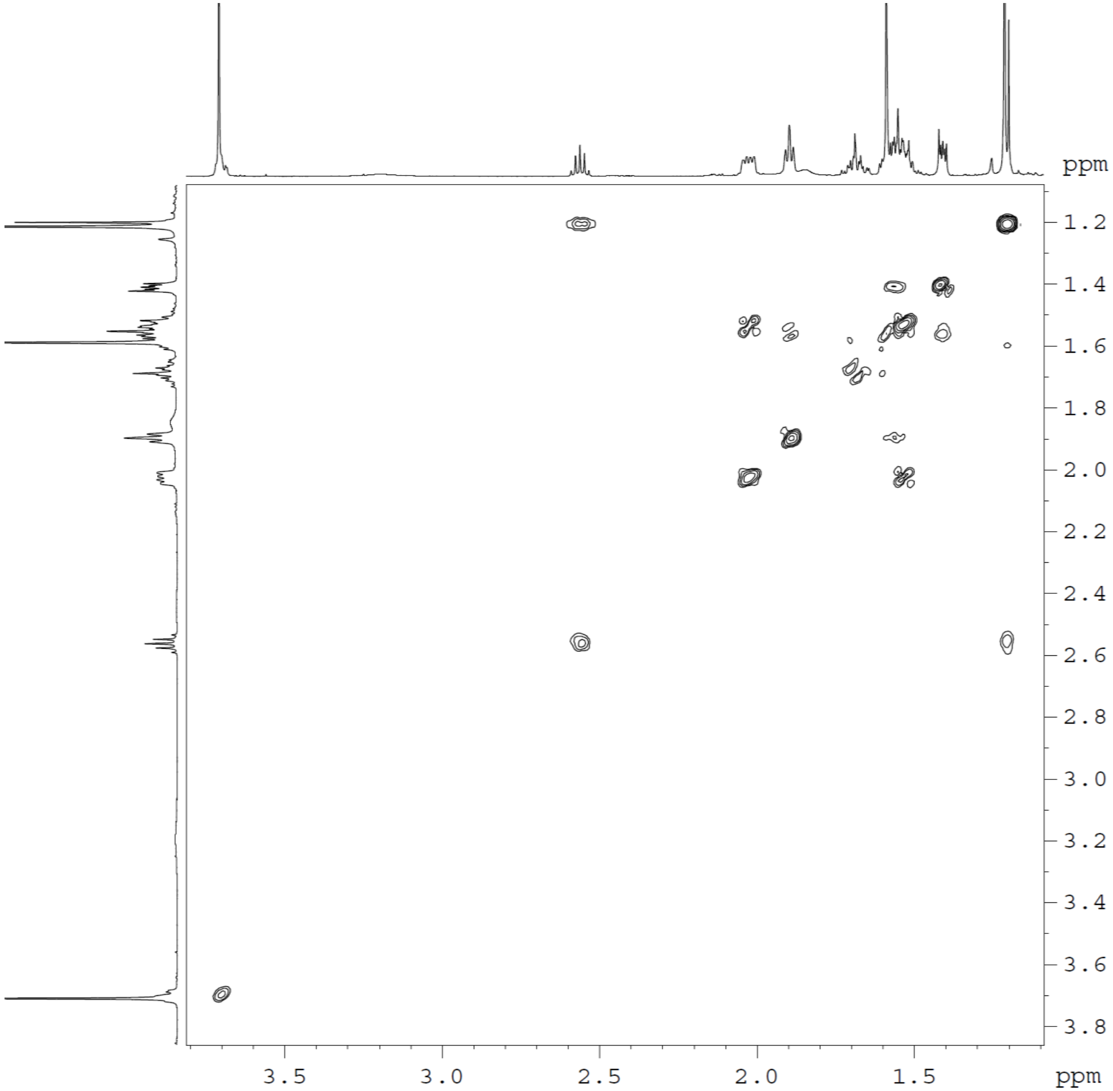

Figure S46. HSQC spectrum of diacardiol B (**6**) in $CDCl_3$.

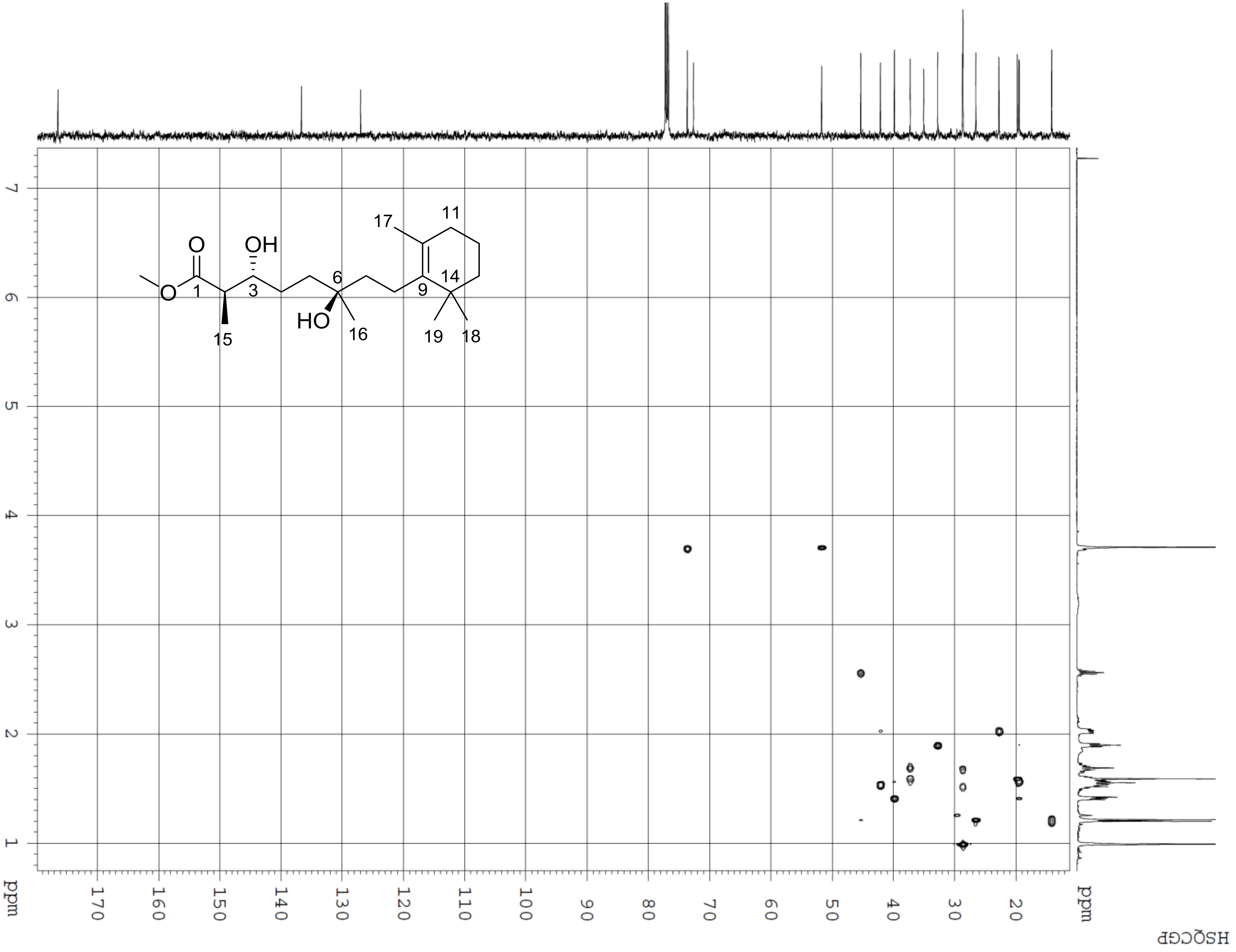

Figure S47. HMBC spectrum of diacardiol B (**6**) in $CDCl_3$.

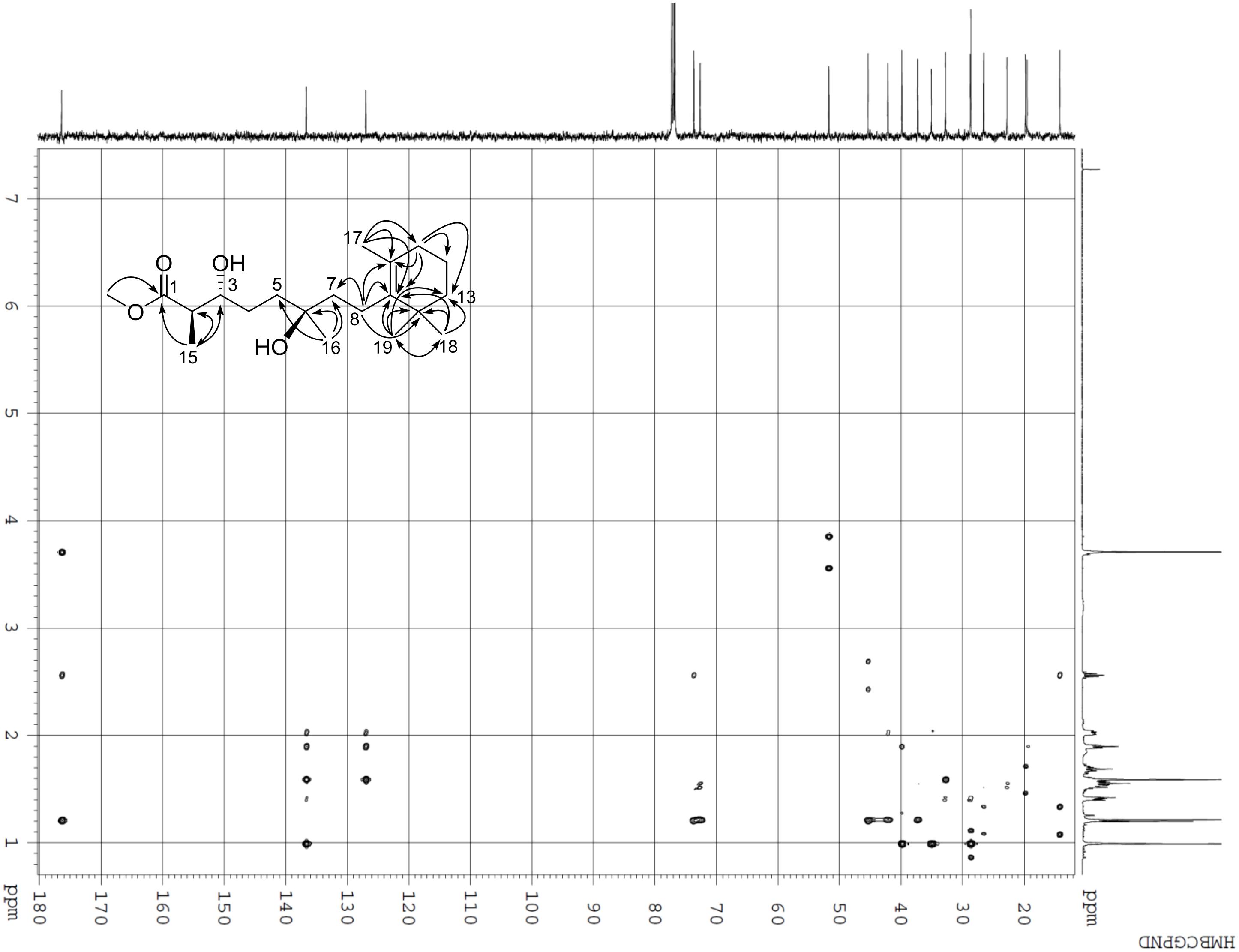

Figure S48. NOESY spectrum of diacardiol B (**6**) in $CDCl_3$.

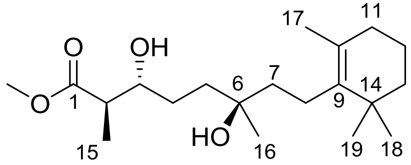

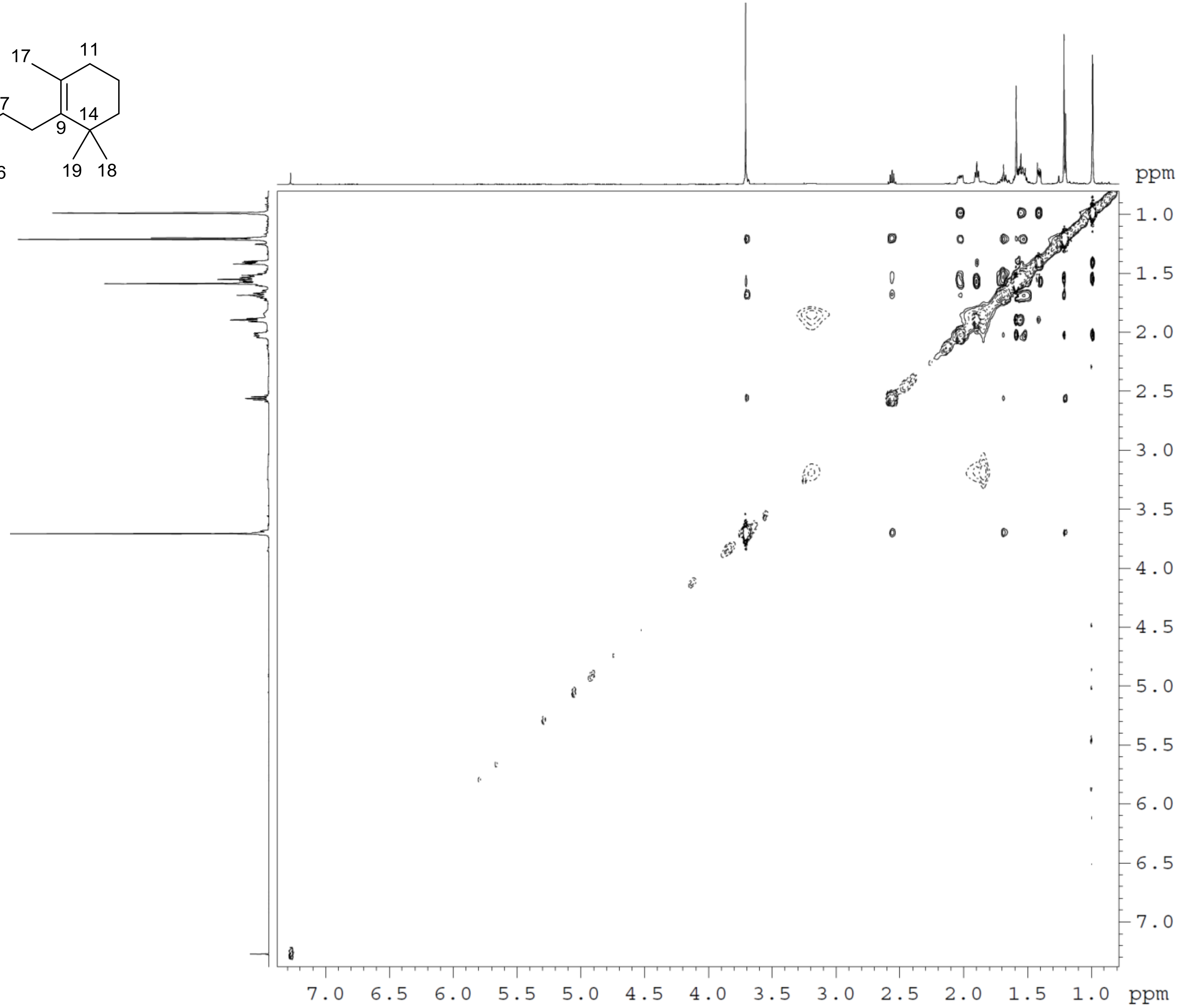

Figure S49. ^{1}H NMR spectrum of (*S*)-MTPA ester **6a** in $CDCl_3$.

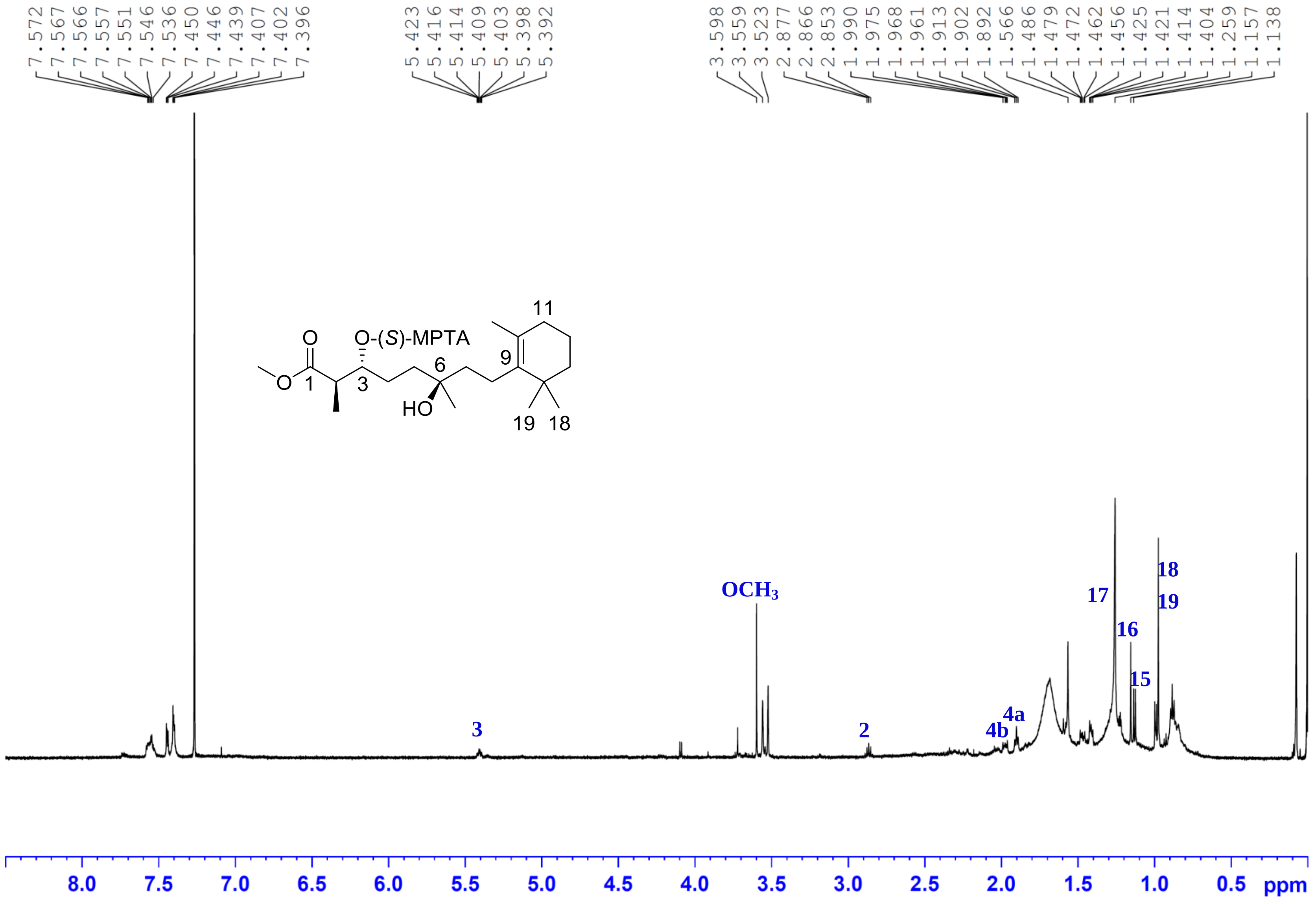

Figure S50. ^{1}H NMR spectrum of (*R*)-MTPA ester **6b** in $CDCl_3$.

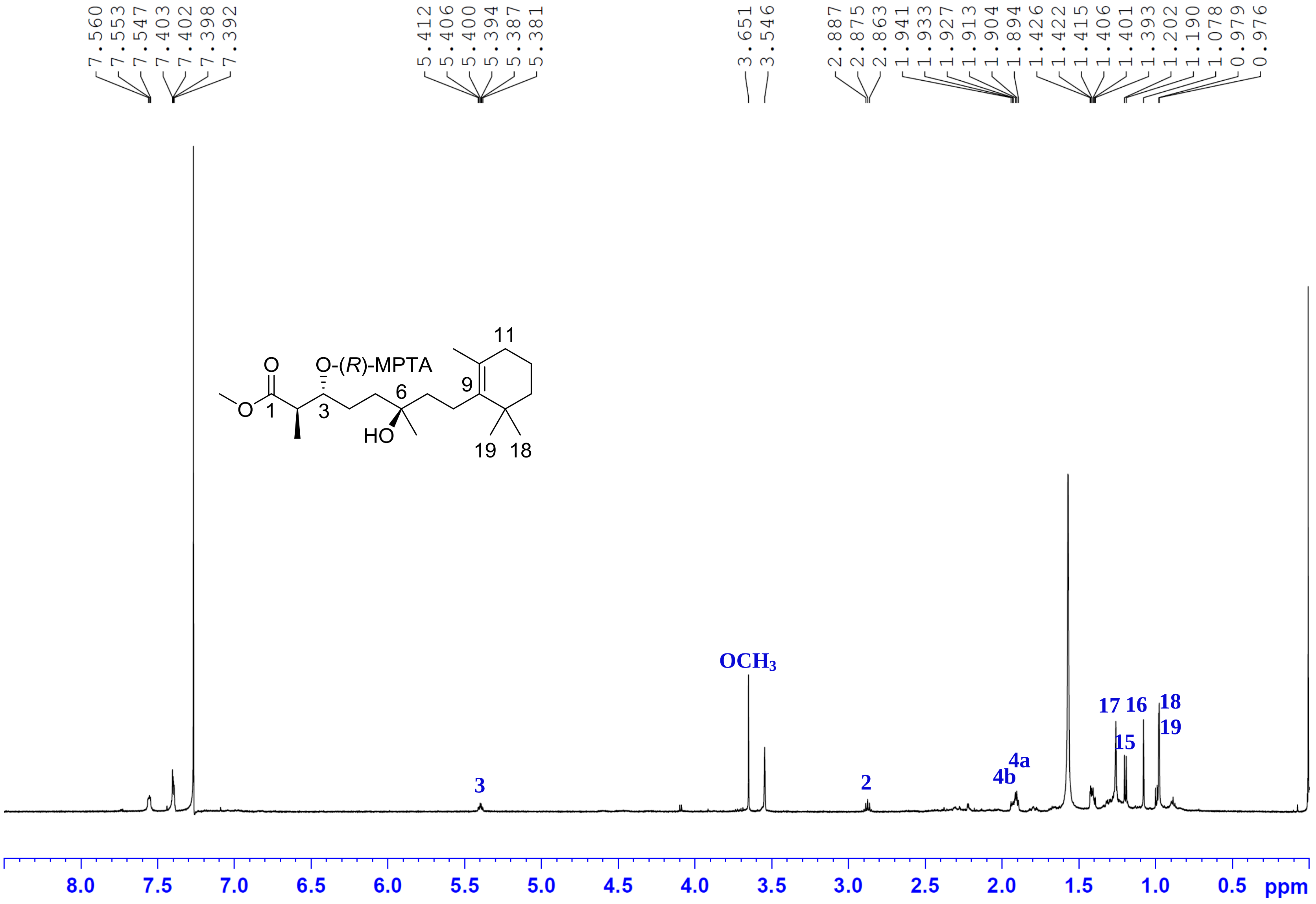

Figure S51. ^{1}H NMR spectrum of diol derived from nuapapuin A methyl ester in $CDCl_3$.

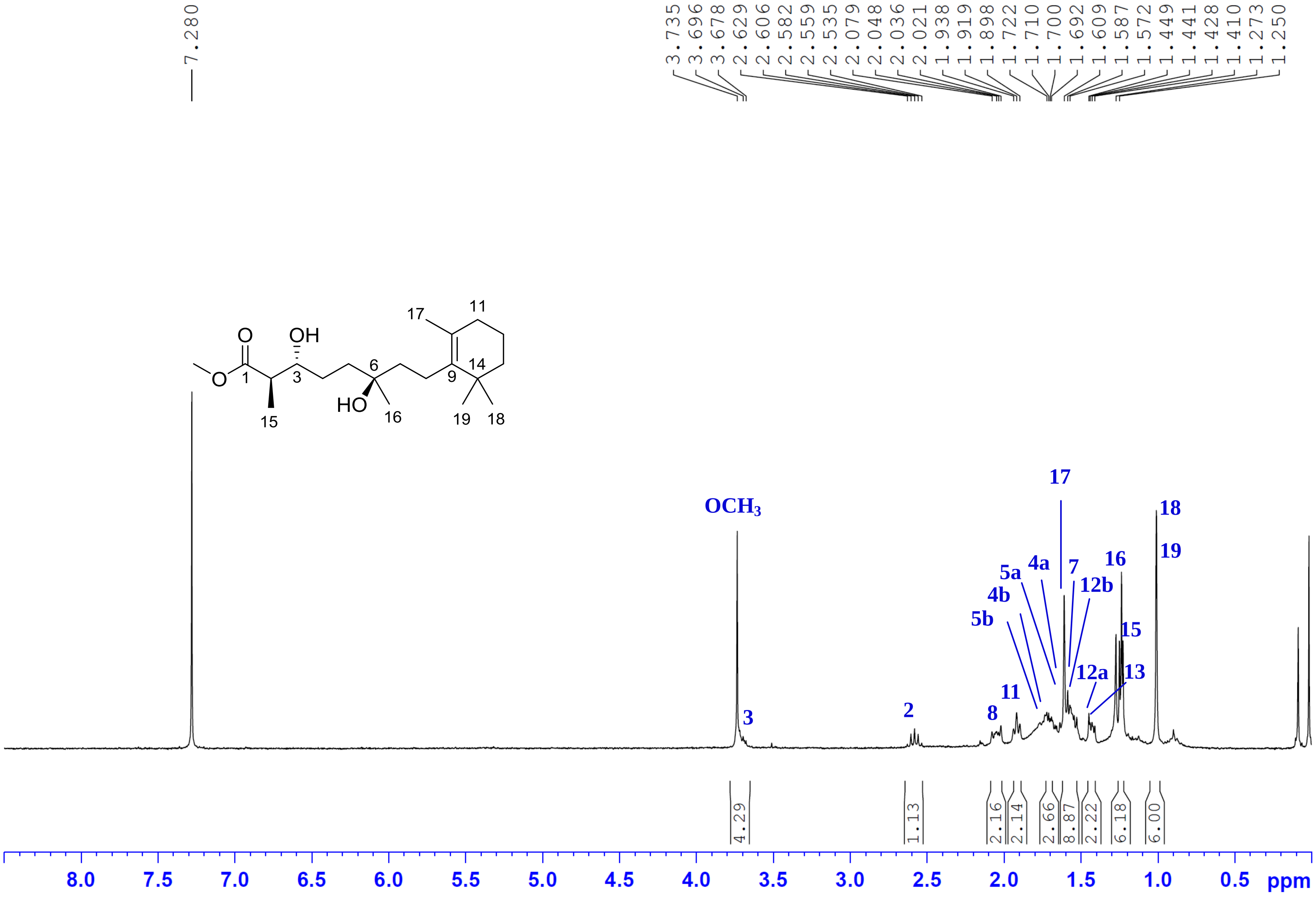

Sections 2. Computation procedure and computational data

Energy minimized structures were used for the DFT calculations applying the PCM solvation models with the dielectric constant representing MeCN or DCM by using Gaussian 09 program. Low energy conformations were optimized by using B3LYP/6-31G(d,p) or MPWLPW91/6-31G(d,p) method applying the PCM solvation model, and the frequency calculations were performed at the same theoretical levels to verify true energy minimal located and generate sets of thermodynamic data at standard condition. The optimized geometries were used for TDDFT calculations at B3LYP/6-31G(d,p) level applying the same solvation model. The generated excitation energies and rotational strengths were Boltzmann averaged and then fitted to Gaussian functions to generate computed ECD spectra normalized and overlaid with the experimental spectra for comparison. The NMR calculations were performed by using the GIAO method at B3LYP/6-31G(d,p) and MPWLPW91/6-31G(d,p) level respectively, and the calculated chemical shifts for TMS at the corresponding theoretical levels were used as the references.

Figure S52. Dominate conformations of compounds **1**–**5**.

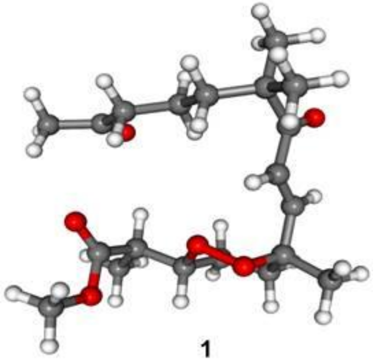

1

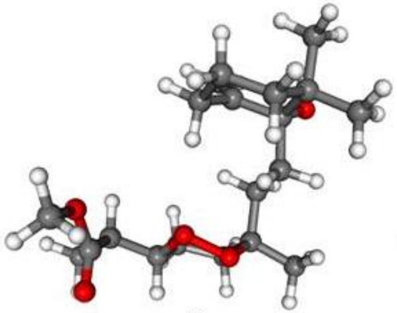

2

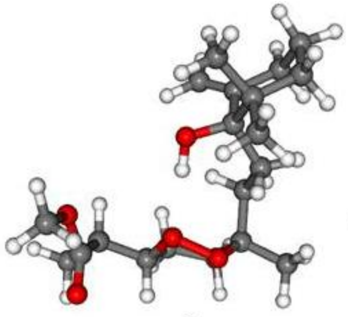

3

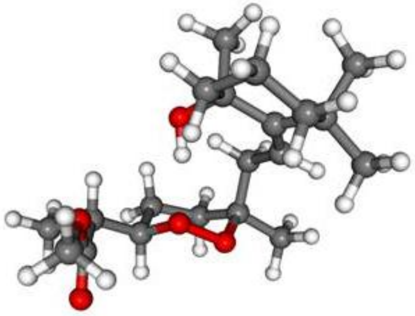

4

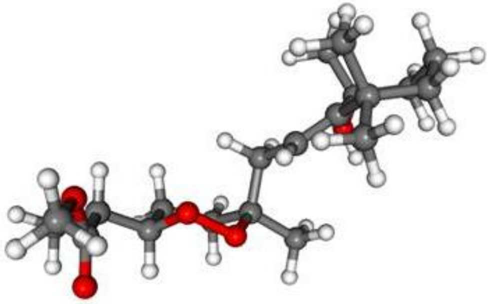

5

Figure S53. Calculated ECD spectra of low energy conformations of **1** at B3LYP/6-31G(d,p) level applying the PCM solvation model (MeCN).

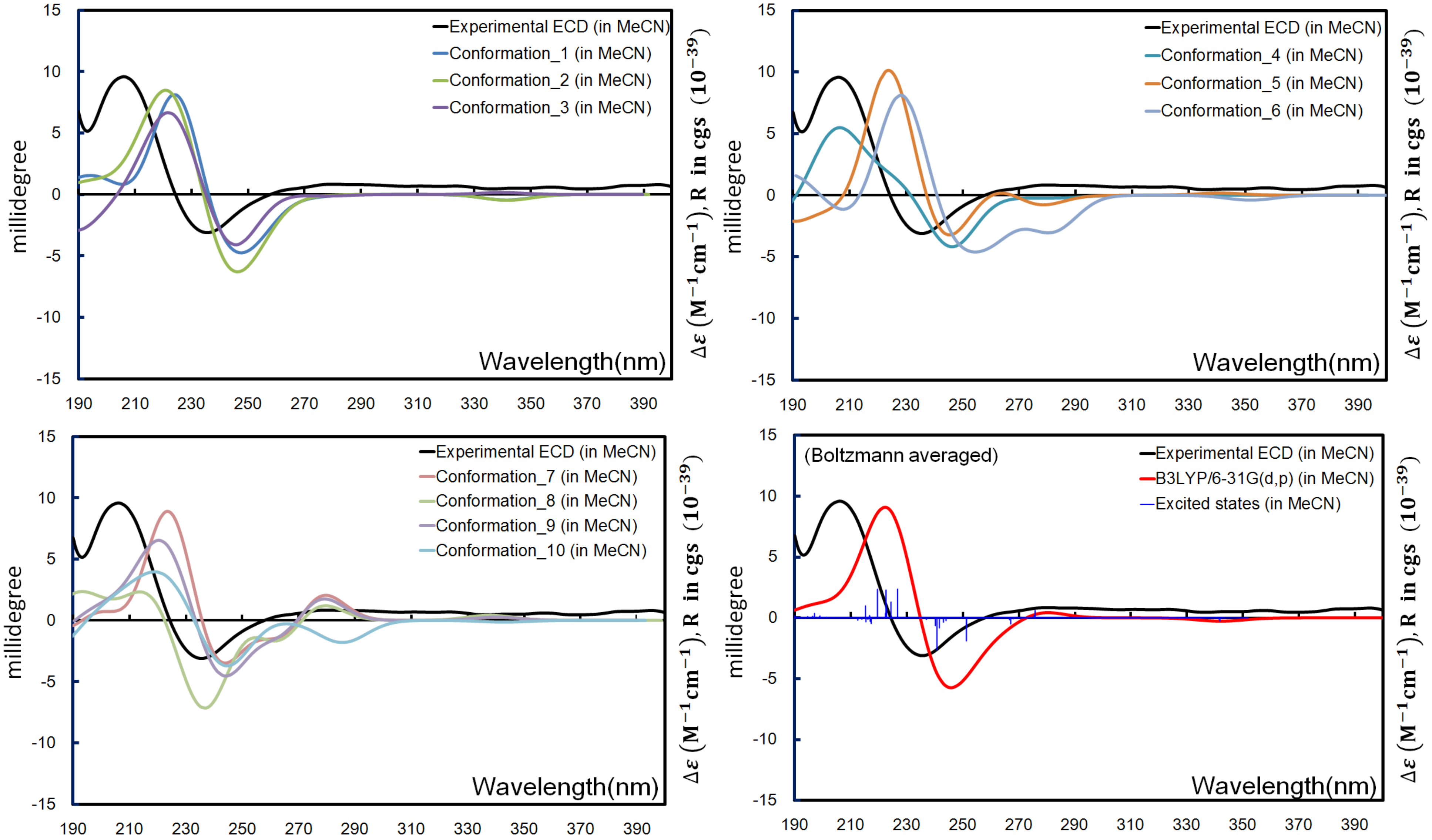

Figure S54. Calculated ECD spectra of low energy conformations of **2** at B3LYP/6-31G(d,p) level applying the PCM solvation model (MeCN).

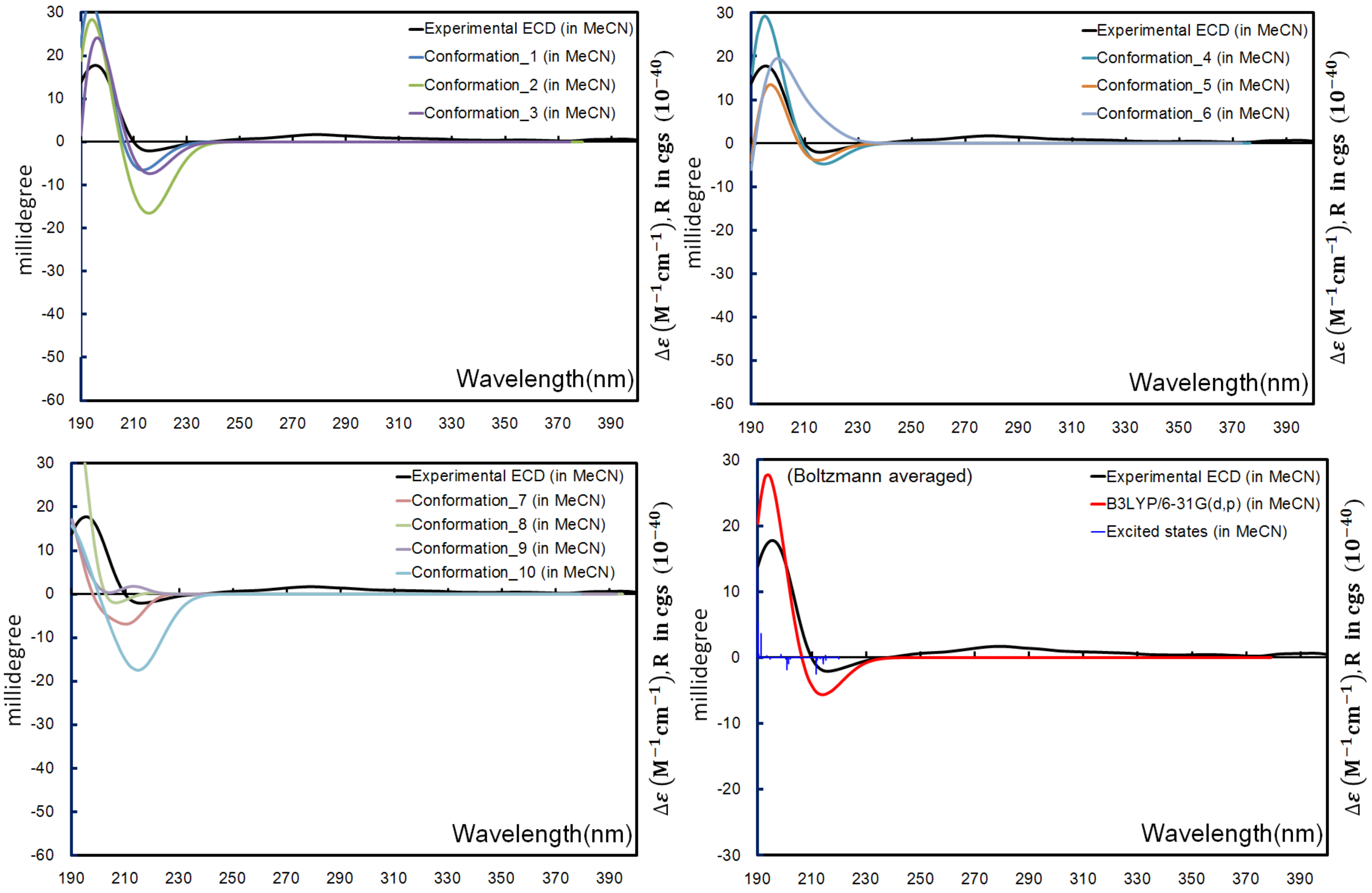

Figure S55. Calculated ECD spectra of low energy conformations of **3** at B3LYP/6-31G(d,p) level applying the PCM solvation model (MeCN).

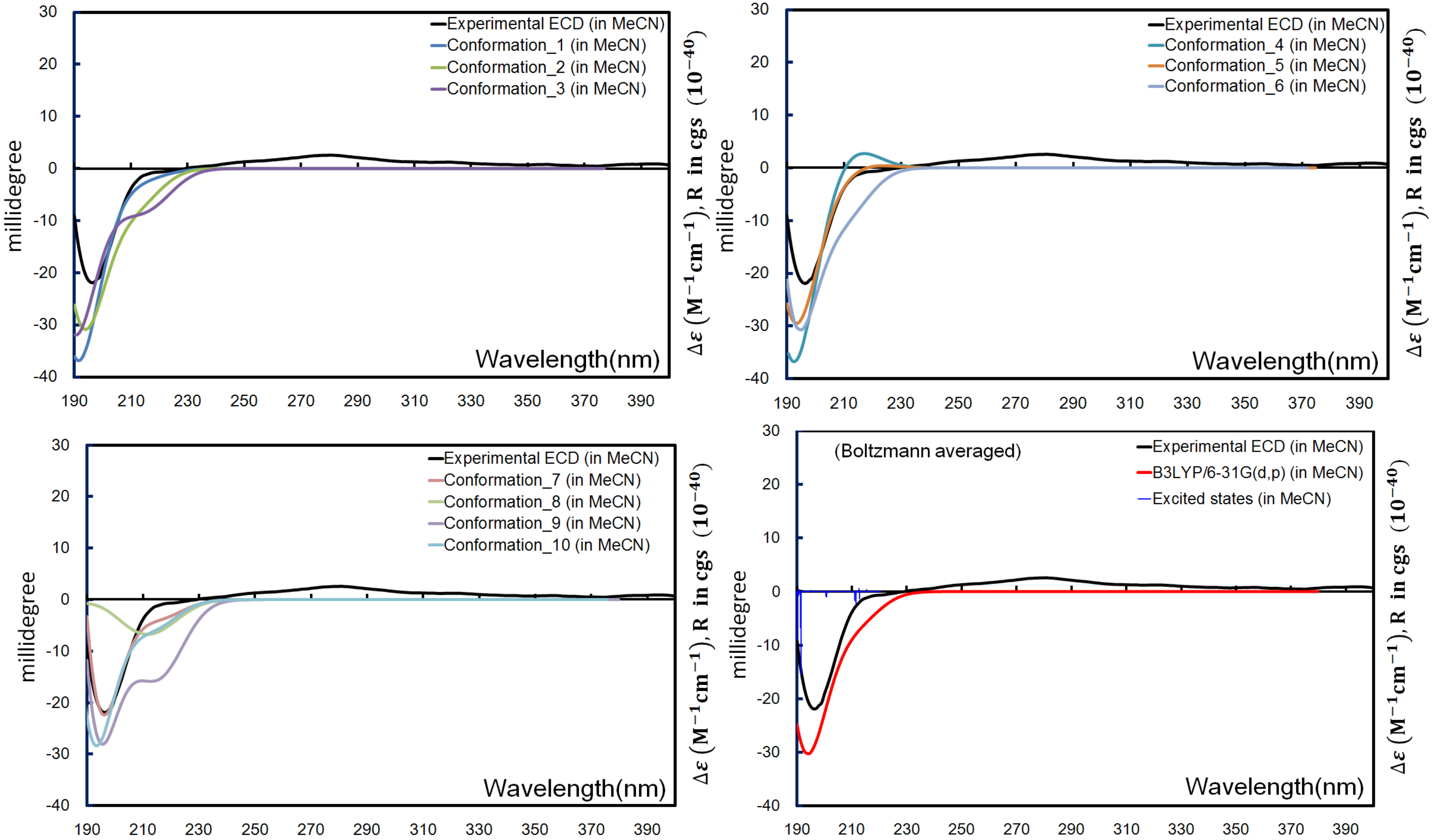

Figure S56. Calculated ECD spectra of low energy conformations of **4** at B3LYP/6-31G(d,p) level applying the PCM solvation model (MeCN).

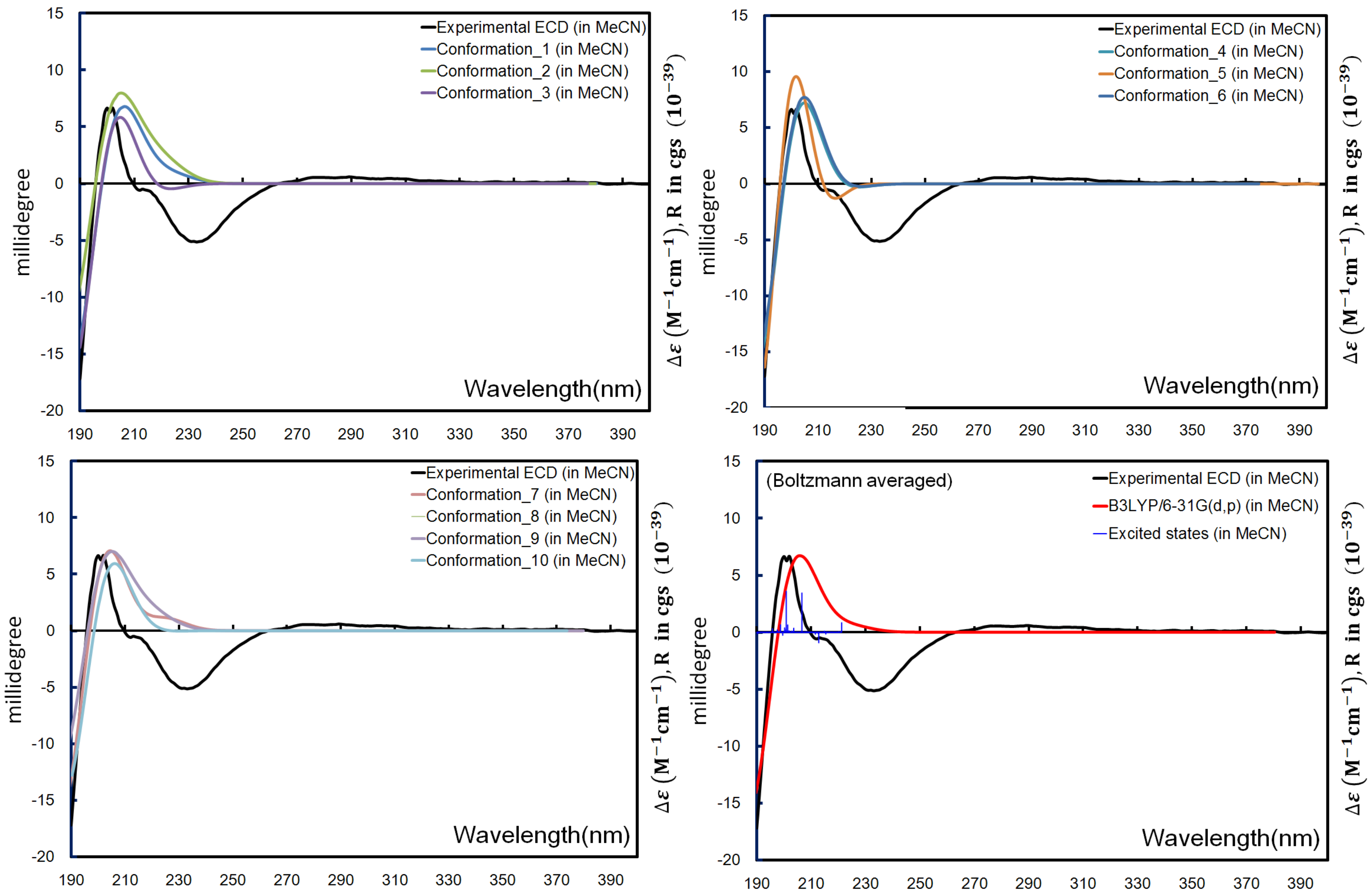

Figure S57. Calculated ECD spectra of low energy conformations of **5** at B3LYP/6-31G(d,p) level applying the PCM solvation model (MeCN).

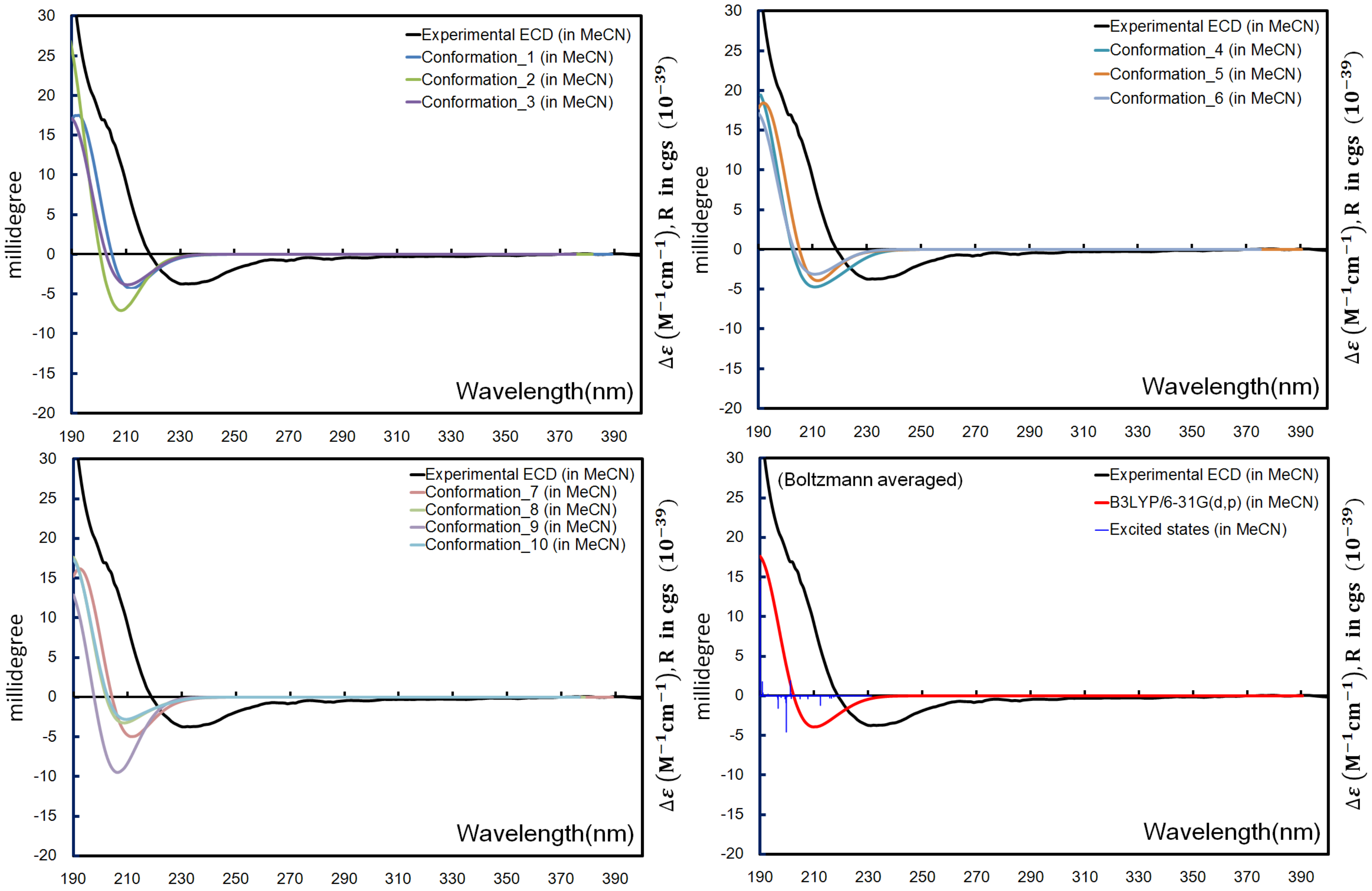

Table S1. Calculated thermodynamic energies (in Hartree) of the low energy conformations of **1** at B3LYP/6-31G(d,p) level applying the PCM solvation model (MeCN).

Species	E_{total}	E_{zpe}	U_{298K}	H_{298K}	G_{298K}
Conf.1	−1232.7235	−1232.2260	−1232.1960	−1232.1951	−1232.2880
Conf.2	−1232.7248	−1232.2269	−1232.1969	−1232.1960	−1232.2889
Conf.3	−1232.7209	−1232.2230	−1232.1933	−1232.1924	−1232.2846
Conf.4	−1232.7214	−1232.2229	−1232.1933	−1232.1924	−1232.2836
Conf.5	−1232.7214	−1232.2236	−1232.1937	−1232.1927	−1232.2868
Conf.6	−1232.7188	−1232.2208	−1232.1910	−1232.1900	−1232.2825
Conf.7	−1232.7207	−1232.2232	−1232.1931	−1232.1921	−1232.2879
Conf.8	−1232.7207	−1232.2226	−1232.1928	−1232.1918	−1232.2856
Conf.9	−1232.7207	−1232.2229	−1232.1930	−1232.1921	−1232.2870
Conf.10	−1232.7207	−1232.2221	−1232.1925	−1232.1915	−1232.2837

Table S2. Calculated relative Boltzmann populations of the low energy conformations of **1** at B3LYP/6-31G(d,p) level applying the PCM solvation model (MeCN).

Species	E_{total}	E_{zpe}	U_{298K}	H_{298K}	G_{298K}
Conf.1	18.83%	25.12%	24.86%	24.84%	18.48%
Conf.2	71.70%	67.33%	66.34%	66.36%	50.41%
Conf.3	1.24%	1.11%	1.44%	1.44%	0.55%
Conf.4	2.04%	0.93%	1.48%	1.48%	0.18%
Conf.5	2.06%	1.97%	2.10%	2.11%	5.40%
Conf.6	0.14%	0.10%	0.12%	0.12%	0.06%
Conf.7	0.97%	1.30%	1.15%	1.15%	16.75%
Conf.8	1.00%	0.71%	0.83%	0.83%	1.59%
Conf.9	1.02%	0.99%	1.06%	1.06%	6.38%
Conf.10	1.01%	0.43%	0.61%	0.61%	0.20%

Table S3. Calculated thermodynamic energies (in Hartree) of the low energy conformations of **2** at B3LYP/6-31G(d,p) level applying the PCM solvation model (MeCN).

Species	E_{total}	E_{zpe}	U_{298K}	H_{298K}	G_{298K}
Conf.1	−1158.6999	−1158.1789	−1158.1518	−1158.1509	−1158.2359
Conf.2	−1158.6977	−1158.1769	−1158.1497	−1158.1488	−1158.2342
Conf.3	−1158.6988	−1158.1780	−1158.1510	−1158.1500	−1158.2343
Conf.4	−1158.6974	−1158.1762	−1158.1493	−1158.1483	−1158.2325
Conf.5	−1158.6964	−1158.1754	−1158.1482	−1158.1473	−1158.2325
Conf.6	−1158.6938	−1158.1727	−1158.1457	−1158.1447	−1158.2296
Conf.7	−1158.6985	−1158.1778	−1158.1506	−1158.1496	−1158.2348
Conf.8	−1158.6992	−1158.1783	−1158.1512	−1158.1502	−1158.2356
Conf.9	−1158.6959	−1158.1753	−1158.1480	−1158.1471	−1158.2328
Conf.10	−1158.6963	−1158.1758	−1158.1485	−1158.1475	−1158.2334

Table S4. Calculated relative Boltzmann populations of the low energy conformations of **2** at B3LYP/6-31G(d,p) level applying the PCM solvation model (MeCN).

Species	E_{total}	E_{zpe}	U_{298K}	H_{298K}	G_{298K}
Conf.1	45.68%	39.86%	41.44%	41.41%	38.85%
Conf.2	4.30%	4.76%	4.58%	4.58%	6.68%
Conf.3	13.09%	15.96%	16.86%	16.86%	7.28%
Conf.4	2.99%	2.22%	2.77%	2.77%	1.09%
Conf.5	1.13%	0.96%	0.93%	0.93%	1.10%
Conf.6	0.07%	0.06%	0.06%	0.06%	0.05%
Conf.7	10.19%	12.53%	11.17%	11.18%	12.29%
Conf.8	20.96%	21.29%	20.22%	20.23%	28.19%
Conf.9	0.62%	0.87%	0.77%	0.77%	1.48%
Conf.10	0.97%	1.47%	1.20%	1.20%	2.99%

Table S5. Calculated thermodynamic energies (in Hartree) of the low energy conformations of **3** at B3LYP/6-31G(d,p) level applying the PCM solvation model (MeCN).

Species	E_{total}	E_{zpe}	U_{298K}	H_{298K}	G_{298K}
Conf.1	−1158.6994	−1158.1780	−1158.1513	−1158.1504	−1158.2329
Conf.2	−1158.6999	−1158.1790	−1158.1520	−1158.1510	−1158.2360
Conf.3	−1158.6999	−1158.1786	−1158.1518	−1158.1509	−1158.2333
Conf.4	−1158.6982	−1158.1770	−1158.1501	−1158.1492	−1158.2329
Conf.5	−1158.6991	−1158.1782	−1158.1511	−1158.1502	−1158.2352
Conf.6	−1158.6994	−1158.1783	−1158.1513	−1158.1503	−1158.2354
Conf.7	−1158.6969	−1158.1760	−1158.1490	−1158.1481	−1158.2323
Conf.8	−1158.6970	−1158.1759	−1158.1491	−1158.1481	−1158.2314
Conf.9	−1158.6964	−1158.1754	−1158.1483	−1158.1474	−1158.2327
Conf.10	−1158.6989	−1158.1776	−1158.1508	−1158.1498	−1158.2331

Table S6. Calculated relative Boltzmann populations of the low energy conformations of **3** at B3LYP/6-31G(d,p) level applying the PCM solvation model (MeCN).

Species	E_{total}	E_{zpe}	U_{298K}	H_{298K}	G_{298K}
Conf.1	14.36%	10.77%	13.35%	13.35%	1.64%
Conf.2	23.97%	30.36%	26.36%	26.34%	46.65%
Conf.3	23.25%	18.59%	23.04%	23.05%	2.69%
Conf.4	3.90%	3.62%	3.82%	3.82%	1.76%
Conf.5	10.41%	12.39%	10.42%	10.43%	19.79%
Conf.6	13.52%	14.66%	12.61%	12.61%	22.77%
Conf.7	0.99%	1.21%	1.18%	1.18%	0.94%
Conf.8	1.12%	1.09%	1.24%	1.24%	0.36%
Conf.9	0.58%	0.68%	0.57%	0.57%	1.37%
Conf.10	7.90%	6.62%	7.42%	7.42%	2.04%

Table S7. Calculated thermodynamic energies (in Hartree) of the low energy conformations of **4** at B3LYP/6-31G(d,p) level applying the PCM solvation model (MeCN).

Species	E_{total}	E_{zpe}	U_{298K}	H_{298K}	G_{298K}
Conf.1	−1158.6984	−1158.1773	−1158.1503	−1158.1493	−1158.2338
Conf.2	−1158.6966	−1158.1751	−1158.1484	−1158.1475	−1158.2300
Conf.3	−1158.6938	−1158.1733	−1158.1461	−1158.1452	−1158.2301
Conf.4	−1158.6950	−1158.1746	−1158.1472	−1158.1463	−1158.2318
Conf.5	−1158.6939	−1158.1739	−1158.1465	−1158.1456	−1158.2318
Conf.6	−1158.6932	−1158.1730	−1158.1456	−1158.1447	−1158.2304
Conf.7	−1158.6928	−1158.1726	−1158.1453	−1158.1443	−1158.2303
Conf.8	−1158.6930	−1158.1728	−1158.1454	−1158.1445	−1158.2308
Conf.9	−1158.6972	−1158.1760	−1158.1492	−1158.1482	−1158.2312
Conf.10	−1158.6910	−1158.1711	−1158.1436	−1158.1427	−1158.2299

Table S8. Calculated relative Boltzmann populations of the low energy conformations of **4** at B3LYP/6-31G(d,p) level applying the PCM solvation model (MeCN).

Species	E_{total}	E_{zpe}	U_{298K}	H_{298K}	G_{298K}
Conf.1	67.83%	66.67%	64.58%	64.58%	69.65%
Conf.2	10.06%	6.75%	9.12%	9.12%	1.18%
Conf.3	0.49%	1.05%	0.83%	0.83%	1.44%
Conf.4	1.80%	3.93%	2.61%	2.61%	7.89%
Conf.5	0.61%	1.98%	1.25%	1.25%	7.82%
Conf.6	0.28%	0.72%	0.48%	0.48%	1.95%
Conf.7	0.18%	0.51%	0.33%	0.33%	1.62%
Conf.8	0.22%	0.60%	0.39%	0.39%	2.87%
Conf.9	18.50%	17.69%	20.36%	20.36%	4.48%
Conf.10	0.03%	0.10%	0.06%	0.06%	1.10%

Table S9. Calculated thermodynamic energies (in Hartree) of the low energy conformations of **5** at B3LYP/6-31G(d,p) level applying the PCM solvation model (MeCN).

Species	E_{total}	E_{zpe}	U_{298K}	H_{298K}	G_{298K}
Conf.1	−1158.6945	−1158.1734	−1158.1464	−1158.1454	−1158.2297
Conf.2	−1158.6961	−1158.1746	−1158.1478	−1158.1468	−1158.2309
Conf.3	−1158.6956	−1158.1753	−1158.1479	−1158.1470	−1158.2336
Conf.4	−1158.6936	−1158.1735	−1158.1460	−1158.1451	−1158.2317
Conf.5	−1158.6939	−1158.1727	−1158.1458	−1158.1448	−1158.2284
Conf.6	−1158.6930	−1158.1729	−1158.1453	−1158.1443	−1158.2314
Conf.7	−1158.6912	−1158.1703	−1158.1431	−1158.1422	−1158.2270
Conf.8	−1158.6937	−1158.1733	−1158.1460	−1158.1451	−1158.2306
Conf.9	−1158.6928	−1158.1726	−1158.1453	−1158.1443	−1158.2305
Conf.10	−1158.6949	−1158.1745	−1158.1472	−1158.1462	−1158.2321

Table S10. Calculated relative Boltzmann populations of the low energy conformations of **5** at B3LYP/6-31G(d,p) level applying the PCM solvation model (MeCN).

Species	E_{total}	E_{zpe}	U_{298K}	H_{298K}	G_{298K}
Conf.1	7.65%	5.13%	6.43%	6.44%	1.05%
Conf.2	41.37%	19.11%	28.03%	28.05%	3.83%
Conf.3	25.18%	39.17%	33.14%	33.12%	61.85%
Conf.4	3.06%	5.89%	4.55%	4.55%	8.79%
Conf.5	4.12%	2.62%	3.51%	3.51%	0.26%
Conf.6	1.72%	3.08%	2.07%	2.07%	6.08%
Conf.7	0.24%	0.20%	0.21%	0.21%	0.06%
Conf.8	3.37%	4.75%	4.67%	4.67%	2.62%
Conf.9	1.37%	2.38%	2.04%	2.04%	2.45%
Conf.10	11.90%	17.66%	15.36%	15.35%	12.99%

Table S11. Cartesian coordinates of the lowest energy conformation of **1**–**5** after DFT optimization at B3LYP/6-31G(d,p) level applying the PCM solvation model (MeCN).

Compound 1_Conf.2				Compound 2_Conf.1				Compound 3_Conf.2				Compound 4_Conf.1				Compound 5_Conf.3			
**Symbol**	**X**	**Y**	**Z**	**Symbol**	**X**	**Y**	**Z**	**Symbol**	**X**	**Y**	**Z**	**Symbol**	**X**	**Y**	**Z**	**Symbol**	**X**	**Y**	**Z**
C	1.986029	1.450780	−0.480225	C	2.780733	0.958869	−0.105408	C	2.747271	−0.911767	0.138818	C	−2.690534	0.780467	0.054862	C	3.125244	−0.995662	−0.060984
C	1.349962	2.071760	−1.723414	C	2.353533	2.065902	−1.068819	C	2.317569	−2.099891	0.998569	C	−2.521006	1.895266	1.084837	C	2.513871	−2.229746	0.600221
C	0.561916	3.329603	−1.332757	C	1.478723	3.100216	−0.341198	C	1.527465	−3.120727	0.160334	C	−1.780930	3.090824	0.461899	C	1.381417	−2.791973	−0.275096
C	−0.366232	3.081961	−0.124703	C	0.385165	2.451535	0.532378	C	0.453455	−2.455999	−0.721591	C	−0.523073	2.669245	−0.321533	C	0.414108	−1.701791	−0.778016
O	0.416132	2.489827	0.939768	O	1.034321	1.457126	1.378545	O	1.112907	−1.397601	−1.479629	O	−0.905869	1.610780	−1.246140	O	1.214585	−0.640561	−1.373979
O	0.956138	1.206347	0.497964	O	1.611281	0.414649	0.538272	O	1.593347	−0.379423	−0.554381	O	−1.397152	0.455959	−0.507090	O	2.074848	−0.052628	−0.353737
C	2.623625	0.068390	−0.707542	C	−0.733869	1.812934	−0.315624	C	−0.716682	−1.891141	0.109830	C	0.624757	2.223607	0.625554	C	−0.491859	−1.156386	0.351761
C	3.061915	−0.544525	0.617538	C	−1.744058	0.986105	0.490322	C	−1.646556	−0.932250	−0.666631	C	1.831896	−2.199982	0.760355	C	−5.070003	−0.594560	0.028366
O	3.951037	0.234076	1.258627	C	−2.763020	0.198983	−0.372794	C	−2.035130	0.339734	0.139114	C	3.182715	−2.590377	0.168384	C	−5.578119	0.843797	0.046990
O	2.686824	−1.620334	1.045917	C	−2.034546	−0.710271	−1.371595	C	−2.689878	−0.059278	1.467516	C	3.493984	−1.679812	−1.016958	C	−4.621839	1.717043	−0.761833
C	4.437655	−0.263643	2.522037	C	−2.208269	−2.738837	0.127979	C	−5.029409	0.183954	0.566479	C	3.527007	−0.168121	−0.666299	C	−3.164840	1.719187	−0.231062
C	−1.566224	2.240510	−0.487145	C	−2.949598	−1.868330	1.149334	C	−4.425420	0.604913	−0.778809	C	2.268862	0.256069	0.152408	C	−2.645209	0.280255	0.064841
C	−2.055202	1.223796	0.233458	C	3.445031	−0.255791	−0.774227	O	−0.845681	1.102421	0.390058	C	1.739696	−0.728673	1.229093	C	−3.645926	−0.776267	0.600570
C	−3.239723	0.447960	−0.229702	C	3.756473	−1.299507	0.291376	C	3.310483	0.287356	0.918753	C	−3.200623	−0.557695	0.618495	C	4.108543	−0.206459	0.818387
C	−3.681963	−0.760328	0.619071	O	3.175624	−2.481746	0.022335	C	3.640097	1.404389	−0.065086	C	−4.600320	−0.425014	1.245567	C	5.315980	−1.059241	1.251738
C	−2.474906	−1.683264	0.954579	C	3.441143	−3.542844	0.963723	O	2.975856	2.536722	0.220117	C	−3.250319	−1.577048	−0.514223	C	4.610064	1.003183	0.038905
C	−1.630142	−2.149648	−0.238843	O	4.467371	−1.101951	1.258651	C	3.241175	3.659458	−0.647552	O	−3.941184	−1.462029	−1.508710	O	5.165912	0.941518	−1.041590
C	−0.468783	−3.046144	0.195450	C	4.747417	0.122828	−1.503723	O	4.426518	1.295026	−0.986639	O	−2.451625	−2.631739	−0.277696	O	4.396481	2.152274	0.702694
C	0.485041	−3.422657	−0.925973	C	−0.150372	3.455017	1.557865	C	4.580427	−0.082167	1.707881	C	−2.446304	−3.658291	−1.292787	C	4.869639	3.351966	0.055105
O	−3.847445	0.767025	−1.247672	C	−2.052809	−0.490432	−2.690839	C	−0.023559	−3.400275	−1.826983	C	2.462295	−0.537735	2.575019	O	−3.241987	−2.118800	0.249632
C	−4.262300	−0.205702	1.944625	H	3.233915	2.564880	−1.485590	C	−2.110609	0.173514	2.649446	O	0.355336	−0.498779	1.546244	C	−3.673272	−0.788977	2.137695
C	−4.778665	−1.540508	−0.123380	H	1.810698	1.615456	−1.908126	H	3.198798	−2.587978	1.425375	C	4.814535	0.148267	0.140120	C	−2.311904	2.446873	−1.294514
C	1.533964	−4.465174	−0.602737	H	2.116316	3.707232	0.312596	H	1.715765	−1.733305	1.838040	C	3.643084	0.586535	−2.009894	C	−3.093592	2.562579	1.069632
O	0.435304	−2.885778	−2.024476	H	1.018322	3.782247	−1.064235	H	2.220373	−3.658390	−0.497367	C	1.742101	1.477541	−0.063268	C	−1.324301	0.037445	−0.045120
C	−0.837678	4.409886	0.490008	H	−1.253254	2.615999	−0.849539	H	1.061119	−3.864555	0.815235	C	−0.083903	3.779802	−1.281289	C	−0.404098	−2.206227	−1.968030
H	2.726339	2.131495	−0.040635	H	−0.278691	1.179844	−1.079708	H	−1.280278	−2.742952	0.505190	H	−3.339496	1.100575	−0.769924	H	3.618986	−1.262702	−1.004595
H	2.118815	2.336202	−2.455563	H	−2.317390	1.647147	1.144542	H	−0.312331	−1.377197	0.984030	H	−3.499472	2.223957	1.447313	H	3.276300	−3.001391	0.745079
H	0.689151	1.331611	−2.190684	H	−1.197968	0.295250	1.136184	H	−1.133397	−0.596519	−1.571091	H	−1.971446	1.499134	1.946224	H	2.140856	−1.953128	1.593488
H	1.261562	4.125564	−1.053816	H	−2.927241	−3.278959	−0.499233	H	−2.544302	−1.459524	−0.996692	H	−2.456096	3.607873	−0.230315	H	1.821228	−3.281487	−1.152373

Table S11. *Cont.*

Compound 1_Conf.2				Compound 2_Conf.1				Compound 3_Conf.2				Compound 4_Conf.1				Compound 5_Conf.3			
Symbol	X	Y	Z	Symbol	X	Y	Z	Symbol	X	Y	Z	Symbol	X	Y	Z	Symbol	X	Y	Z
H	−0.024050	3.696288	−2.181724	H	−1.614572	−3.500638	0.646274	H	−5.973247	−0.349255	0.402694	H	−1.504896	3.810736	1.239899	H	0.821948	−3.557773	0.272809
H	4.942871	−1.221779	2.384816	H	2.914939	−4.414732	0.578440	H	−5.271444	1.070105	1.165017	H	1.040829	3.150026	1.047289	H	−1.105879	−1.976839	0.719097
H	3.611632	−0.386835	3.225426	H	4.513533	−3.738895	1.025113	H	−4.351229	−0.277321	−1.427758	H	0.227634	1.651841	1.458729	H	0.159736	−0.834785	1.173483
H	5.137121	0.488562	2.883086	H	3.065107	−3.276146	1.953625	H	−5.102313	1.299433	−1.292639	H	1.057086	−2.348398	−0.004258	H	−5.739979	−1.271557	0.570245
H	−2.079171	2.516121	−1.407562	H	4.551041	0.827924	−2.314274	H	−0.068493	0.603533	0.092582	H	1.560584	−2.838064	1.609297	H	−5.054983	−0.938563	−1.015586
H	−1.558916	0.924711	1.148836	H	5.461586	0.574714	−0.809704	H	2.961366	3.419586	−1.675346	H	3.972646	−2.526322	0.926823	H	−6.584986	0.888595	−0.384202
H	−1.818640	−1.177392	1.673232	H	5.210681	−0.766304	−1.940452	H	4.299472	3.925980	−0.613073	H	3.159276	−3.636572	−0.158898	H	−5.669761	1.212467	1.076052
H	−2.874484	−2.557108	1.486130	H	0.666391	3.854446	2.165499	H	2.629213	4.475017	−0.265863	H	4.455583	−1.949936	−1.470610	H	−4.618217	1.355367	−1.799688
H	−2.250819	−2.690230	−0.962063	H	−0.631506	4.287934	1.036716	H	4.365452	−0.845409	2.458595	H	2.728956	−1.842850	−1.789029	H	−4.977124	2.754235	−0.796433
H	−1.220614	−1.285381	−0.772482	H	−0.883157	2.995310	2.224831	H	5.357398	−0.457025	1.035903	H	−2.490379	−0.912419	1.371646	H	3.575149	0.151312	1.704211
H	−0.830317	−3.964599	0.676169	H	−1.540148	−1.158449	−3.377657	H	4.971987	0.796652	2.227413	H	−5.322019	−0.065591	0.506953	H	6.010625	−0.459481	1.846387
H	0.148038	−2.541633	0.953526	H	−2.557388	0.365696	−3.126187	H	−0.453625	−4.299173	−1.375906	H	−4.943201	−1.395530	1.614620	H	4.996856	−1.905109	1.864206
H	−4.580671	−1.036239	2.583630	O	−3.518365	1.216630	−1.044859	H	−0.786057	−2.928516	−2.452088	H	−3.447033	−4.077365	−1.415819	H	5.853426	−1.440224	0.378743
H	−5.133860	0.429709	1.757201	H	−4.116116	0.778200	−1.666097	H	0.812676	−3.701180	−2.464372	H	−2.105839	−3.248331	−2.245771	H	4.615170	4.168317	0.729141
H	−3.525630	0.380871	2.502251	H	2.737895	−0.691097	−1.486661	H	−2.593372	−0.117828	3.578900	H	−1.754817	−4.419430	−0.935353	H	5.949999	3.303330	−0.096116
H	−5.110904	−2.386633	0.486625	C	−1.293599	−1.884449	−0.767244	H	−1.146681	0.664935	2.713599	H	3.507199	−0.849392	2.535250	H	4.375139	3.483987	−0.909436
H	−4.424793	−1.926698	−1.082751	H	−0.456301	−1.507431	−0.163623	H	2.542066	0.649450	1.608621	H	2.427197	0.510524	2.883527	H	−3.116192	−2.131588	−0.710001
H	−5.642002	−0.902214	−0.326685	H	−0.854089	−2.493829	−1.564002	C	−4.052301	−0.708684	1.353251	H	1.956560	−1.141686	3.335538	H	−4.298871	−1.618966	2.482163
H	1.071530	−5.377246	−0.211535	C	−3.734103	−0.689392	0.517145	H	−4.445771	−0.929565	2.351136	H	−0.124943	−0.335608	0.715608	H	−2.665442	−0.920524	2.539559
H	2.194977	−4.070692	0.176640	H	−3.652314	−2.482470	1.726610	H	−3.964020	−1.672511	0.830992	H	4.899620	−0.437637	1.057505	H	−4.083877	0.138358	2.541857
H	2.119743	−4.697590	−1.493548	H	−2.227412	−1.473452	1.875015	C	−3.031517	1.275758	−0.662756	H	5.699286	−0.068428	−0.469870	H	−2.212400	1.847411	−2.204851
H	−1.433480	4.964340	−0.239981	C	−4.876666	−1.244252	−0.366106	C	−3.160030	2.625337	0.078195	H	4.843051	1.206994	0.416309	H	−2.796601	3.392220	−1.561402
H	−1.453208	4.225845	1.373878	H	−4.510249	−1.742903	−1.267517	H	−3.452600	2.501754	1.123846	H	4.451809	0.144330	−2.601384	H	−1.308796	2.693992	−0.936349
H	0.024604	5.020187	0.772510	H	−5.562409	−0.448040	−0.674862	H	−3.913352	3.250652	−0.413736	H	2.718837	0.514342	−2.591480	H	−3.733537	2.172075	1.864787
H	1.862897	−0.607663	−1.109258	H	−5.467725	−1.971011	0.200804	H	−2.208272	3.160925	0.064475	H	3.886410	1.645247	−1.881034	H	−2.068334	2.588722	1.451399
C	3.812130	0.137611	−1.686311	C	−4.380336	0.142304	1.642101	C	−2.498167	1.570372	−2.077685	H	2.208925	2.067271	−0.846190	H	−3.410364	3.592759	0.868058
H	3.478867	0.458881	−2.675701	H	−3.660100	0.427453	2.414399	H	−3.108645	2.345888	−2.553414	H	0.149898	4.682365	−0.709531	H	−0.707729	0.845104	−0.428125
H	4.271833	−0.848613	−1.794838	H	−5.167878	−0.441797	2.130811	H	−2.527358	0.690037	−2.726999	H	0.801600	3.492580	−1.853260	H	−0.983806	−3.083968	−1.666259
H	4.575137	0.834859	−1.330362	H	−4.834945	1.052856	1.240705	H	−1.466753	1.932817	−2.033764	H	−0.886908	4.016057	−1.984911	H	−1.093375	−1.436493	−2.324746
				H	3.446734	1.352373	0.673883	H	3.472170	−1.221358	−0.624301	H	−4.586008	0.266916	2.090194	H	0.252719	−2.498345	−2.792105

Figure S58. *A*-value (Boltzmann averaged) study of the endoperoxide core: two types (**type I** and **type II**) of simplified substitutions on the C6 position were modeled mimicking the olefinic attachment on **1** and the alkyl attachment on **2**–**5**, respectively.

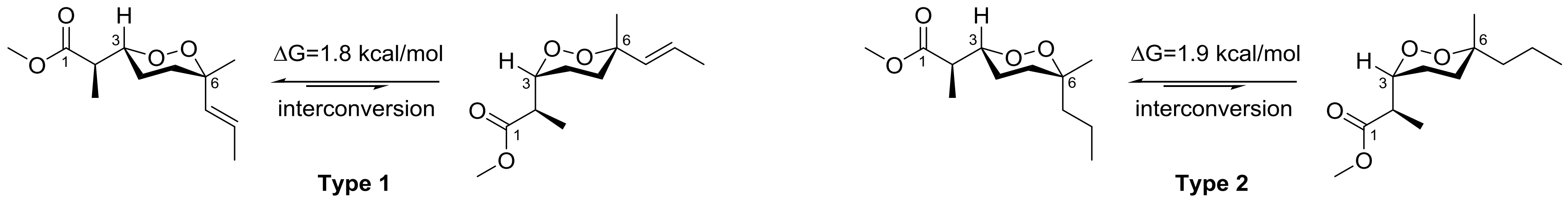

Table S12. Calculated thermodynamic energies (in Hartree) of the low energy conformations of **Type I** and **Type II** model by using B3LYP/6-31G(d,p) method applying the PCM solvation model.

**Species**	$\mathbf{E_{total}}$	$\mathbf{E_{zpe}}$	$\mathbf{U_{298K}}$	$\mathbf{H_{298K}}$	$\mathbf{G_{298K}}$
Type I_6-Methyl_ax_Conf1	−770.1588	−769.8500	−769.8320	−769.8311	−769.8965
Type I _6-Methyl_ax_Conf2	−770.1593	−769.8502	−769.8324	−769.8315	−769.8962
Type I _6-Methyl_ax_Conf3	−770.1595	−769.8505	−769.8326	−769.8317	−769.8967
Type I _6-Methyl_eq_Conf1	−770.1622	−769.8534	−769.8355	−769.8345	−769.8998
Type I _6-Methyl_eq_Conf2	−770.1600	−769.8511	−769.8332	−769.8323	-769.8974
Type II_6-Methyl_eq_Conf3	−770.1603	−769.8516	−769.8336	−769.8327	−769.8986
Type II _6-Methyl_ax_Conf1	−771.3934	−771.0604	−771.0422	−771.0413	−771.1075
Type II _6-Methyl_ax_Conf2	−771.3933	−771.0603	−771.0421	−771.0411	−771.1071
Type II _6-Methyl_ax_Conf3	−771.3926	−771.0597	−771.0415	−771.0406	−771.1068
Type II _6-Methyl_eq_Conf1	−771.3957	−771.0629	−771.0446	−771.0436	−771.1104
Type II _6-Methyl_eq_Conf2	−771.3933	−771.0603	−771.0421	−771.0412	−771.1073
Type II _6-Methyl_eq_Conf3	−771.3927	−771.0597	−771.0415	−771.0405	−771.1066

Table S13. Cartesian coordinates of the lowest energy conformation of **type I** model after DFT optimization at B3LYP/6-31G(d,p) level applying the PCM solvation model.

**Type I 6-Methyl_ax_Conf3**				**Type I 6-Methyl_eq_Conf1**			
**Symbol**	**X**	**Y**	**Z**	**Symbol**	**X**	**Y**	**Z**
C	0.946020	−1.311704	−0.322758	C	0.520402	−0.833434	0.335624
C	−0.135406	−2.258061	0.210483	C	−0.644200	−1.101516	1.288476
C	−1.367629	−1.484158	0.701855	C	−1.887961	−1.535599	0.497600
C	−1.826115	−0.404413	−0.306634	C	−2.160344	−0.620152	−0.709050
O	−0.652470	0.399197	−0.660056	O	−0.928092	−0.537716	−1.483698
O	0.354120	−0.444878	−1.311010	O	0.097355	0.106464	−0.672043
C	1.723403	−0.492157	0.728592	C	1.744522	−0.164977	0.981072
C	2.704455	0.408986	−0.008455	C	2.806839	0.064309	−0.087144
O	2.551244	1.703114	0.322544	O	3.201152	1.348532	−0.135613
O	3.550271	0.010768	−0.787972	O	3.272057	−0.810694	−0.792906
C	3.459942	2.627126	−0.311593	C	4.235198	1.653953	−1.094686
C	−2.688516	0.616593	0.401192	C	−2.628986	0.781414	−0.378675
C	−3.960352	0.918630	0.126708	C	−3.265007	1.184378	0.723730
C	−2.455143	−0.989815	−1.570517	C	−3.125921	−1.278313	−1.705567
H	1.674718	−1.869822	−0.922517	H	0.836606	−1.757026	−0.166919
H	0.259283	−2.887914	1.012875	H	−0.375623	−1.883979	2.004936
H	−2.195931	−2.173186	0.896949	H	−0.851815	−0.188122	1.856982
H	4.492906	2.385611	−0.052316	H	−1.734876	−2.549910	0.111158
H	3.342722	2.592290	−1.396694	H	−2.764065	−1.576840	1.152021
H	3.191007	3.611028	0.069554	H	4.433651	2.718634	−0.982492
H	−2.191829	1.115750	1.233623	H	5.134953	1.072239	−0.884044
H	−3.339978	−1.583245	−1.324700	H	3.891382	1.433835	−2.107469
H	−2.748932	−0.189126	−2.254213	H	−2.448473	1.496203	−1.182240
H	−1.742989	−1.634037	−2.087535	H	−4.079054	−1.478181	−1.209130
H	1.027556	0.143115	1.279367	H	−3.311211	−0.621089	−2.559629
C	2.507118	−1.382878	1.710651	H	−2.713970	−2.223794	−2.071232
H	3.091884	−0.769809	2.402545	H	1.441234	0.807988	1.379279
H	3.196779	−2.040633	1.173089	C	2.349037	−1.018947	2.111925
H	1.830603	−1.999807	2.306496	H	3.235825	−0.529835	2.524555
C	−4.783999	1.909845	0.895921	H	2.643971	−2.003464	1.737893
H	−5.667134	1.429445	1.335224	H	1.632821	−1.152518	2.925423
H	−5.158288	2.702563	0.236413	C	−3.767361	2.580375	0.957902
H	−4.210432	2.375375	1.702500	H	−3.322841	3.013114	1.862806
H	−0.414209	−2.932983	−0.605437	H	−4.853745	2.588150	1.111773
H	−1.138585	−0.989014	1.652301	H	−3.537714	3.239014	0.115143
H	−4.467134	0.420819	−0.698227	H	−3.454142	0.473943	1.528034

Table S14. Cartesian coordinates of the lowest energy conformation of **type II** model after DFT optimization at B3LYP/6-31G(d,p) level applying the PCM solvation model.

**Type II 6-Methyl_ax_Conf1**				**Type II 6-Methyl_eq_Conf1**			
**Symbol**	**X**	**Y**	**Z**	**Symbol**	**X**	**Y**	**Z**
C	1.000446	−1.337659	−0.381617	C	−0.644290	−1.024364	−0.056076
C	0.038448	−2.397205	0.170195	C	0.336159	−1.828650	−0.908137
C	−1.226047	−1.759472	0.764951	C	1.665791	−2.005412	−0.155882
C	−1.822119	−0.674073	−0.155784	C	2.180164	−0.692891	0.471354
O	−0.746059	0.241081	−0.510436	O	1.086111	−0.112996	1.239199
O	0.274790	−0.472086	−1.278627	O	−0.016054	0.210834	0.340816
C	1.782216	−0.512032	0.661670	C	−1.925948	−0.580003	−0.779837
C	2.634270	0.509082	−0.078883	C	−2.783616	0.226310	0.187967
O	2.425429	1.760067	0.368665	O	−3.074281	1.447410	−0.292746
O	3.437135	0.233433	−0.951112	O	−3.185695	−0.189870	1.258215
C	3.224053	2.791708	−0.247081	C	−3.909608	2.271528	0.547295
C	−2.466882	−1.260527	−1.415774	C	3.220870	−0.988813	1.555303
H	1.726950	−1.802791	−1.058463	H	−0.917133	−1.577719	0.852286
H	−0.228592	−3.062194	−0.657712	H	−0.080937	−2.813139	−1.141814
H	0.536531	−3.020821	0.918146	H	0.490080	−1.307656	−1.860245
H	−1.977819	−2.532360	0.959039	H	1.519654	−2.730687	0.653421
H	−0.989943	−1.297894	1.730824	H	2.427852	−2.420393	−0.824501
H	2.931951	3.719719	0.241977	H	−4.042463	3.204979	0.002743
H	4.287013	2.595492	−0.091564	H	−4.873355	1.787659	0.718745
H	3.019647	2.843832	−1.318493	H	−3.421330	2.454477	1.506715
H	−3.359933	−1.834203	−1.148902	H	4.093964	−1.468999	1.103607
H	−2.760246	−0.465833	−2.105582	H	3.549650	−0.075664	2.056350
H	−1.778454	−1.923213	−1.942530	H	2.807048	−1.665482	2.308157
H	1.080070	0.030980	1.296892	H	−1.647952	0.068007	−1.616590
C	2.705141	−1.383844	1.534440	C	−2.745126	−1.775326	−1.301280
H	3.292581	−0.759827	2.214230	H	−3.662832	−1.426176	−1.782761
H	3.399801	−1.957534	0.913525	H	−3.022138	−2.441771	−0.479520
H	2.126223	−2.080977	2.144556	H	−2.177331	−2.345680	−2.039471
C	−2.787769	0.224347	0.641913	C	2.700342	0.295313	−0.591604
H	−2.224571	0.653373	1.480613	H	1.937299	0.409285	−1.368550
H	−3.547391	−0.432764	1.085449	H	3.567054	−0.171194	−1.078114
C	−3.473552	1.356988	−0.135066	C	3.083111	1.689755	−0.078884
H	−2.713493	1.959113	−0.645690	H	2.233185	2.115058	0.466273
H	−4.116354	0.938537	−0.917921	H	3.907417	1.617105	0.640566
C	−4.314559	2.254580	0.778589	C	3.493958	2.632248	−1.215128
H	−3.695960	2.724208	1.551784	H	2.678883	2.763621	−1.936022
H	−5.098317	1.680529	1.286229	H	4.358900	2.240865	−1.763003
H	−4.801932	3.053323	0.210356	H	3.762983	3.622300	−0.832695

Figure S59. Study of the 1,3-diaxial interaction *vs.* 1,3-allylic strain in the monoterpene ring of **4** and **5**: C7 positions were replaced with methyl groups for the model study represented as **m1** and **m2**.

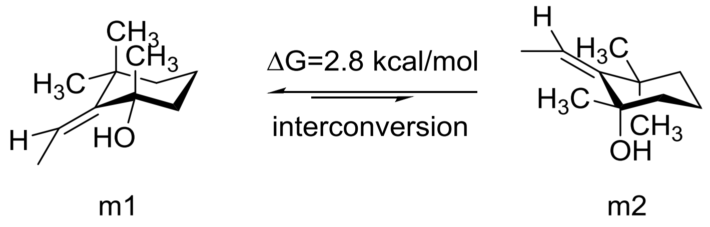

Table S15. Calculated thermodynamic energies (in Hartree) of **m1**and **m2** by using B3LYP/6-31G(d,p) method applying the PCM solvation model.

**Species**	E_{total}	E_{zpe}	U_{298K}	H_{298K}	G_{298K}
**m1**	−506.4543	−506.1633	−506.1499	−506.1489	−506.2013
**m2**	−506.4510	−506.1593	−506.1461	−506.1452	−506.1969

Table S16. Cartesian coordinates of the lowest energy conformations of **m1** and **m2** after DFT optimization at B3LYP/6-31G(d,p) level applying the PCM solvation model.

**m1**				**m2**			
**Symbol**	**X**	**Y**	**Z**	**Symbol**	**X**	**Y**	**Z**
C	0.033819	−1.966160	−0.647839	C	0.034090	−1.904423	−0.757396
H	−0.373336	−2.956759	−0.415600	H	0.269158	−1.710138	−1.811786
C	−0.878771	−0.909213	0.015719	H	0.391274	−2.915776	−0.530567
C	−0.291753	0.522388	−0.079647	C	0.850035	−0.916798	0.100909
C	1.251972	0.699417	0.028187	C	0.321880	0.530996	−0.051467
C	2.016688	−0.467040	−0.650723	C	−1.225438	0.704888	0.042208
H	3.082803	−0.365491	−0.413226	C	−1.942185	−0.374320	−0.813850
H	1.929470	−0.356964	−1.740861	H	−1.770051	−0.143924	−1.875129
C	1.506511	−1.854181	−0.266033	H	−3.023588	−0.287599	−0.648630
H	1.645642	−2.038358	0.806317	C	−1.472494	−1.801073	−0.542361
H	2.086803	−2.625526	−0.785859	H	−1.992364	−2.499609	−1.209030
C	−1.070670	1.618476	−0.158233	H	−1.720511	−2.101265	0.481525
H	−0.555856	2.573248	−0.231397	C	1.088424	1.630848	−0.190560
C	−2.568196	1.789443	−0.118574	H	0.548285	2.569418	−0.255366
H	−2.821670	2.563968	0.616412	C	2.586297	1.847801	−0.259155
H	−3.104990	0.876354	0.124922	H	3.065942	1.309490	−1.081118
H	−2.942289	2.157426	−1.084217	H	3.100220	1.553019	0.661961
O	−2.156189	−1.006119	−0.650134	H	2.788464	2.911768	−0.409121
H	−2.042461	−0.627051	−1.533532	O	0.645190	−1.359275	1.470137
C	−1.192783	−1.295229	1.470650	H	1.049758	−0.692793	2.043377
H	−0.284784	−1.407639	2.066160	C	2.333715	−1.135746	−0.230227
H	−1.728749	−2.250037	1.482669	H	2.577492	−0.833420	−1.252218
H	−1.820563	−0.535559	1.943205	H	2.549021	−2.203982	−0.131633
C	1.655985	0.777345	1.524242	H	2.992105	−0.603249	0.457509
H	1.430986	−0.139785	2.073645	C	−1.717171	2.070015	−0.485542
H	1.128844	1.598762	2.019336	H	−1.367774	2.263543	−1.504875
H	2.733488	0.958854	1.614637	H	−1.405048	2.904998	0.149123
C	1.747956	1.998993	−0.644484	H	−2.812387	2.073206	−0.499135
H	1.434807	2.898763	−0.107376	C	−1.688028	0.600580	1.519097
H	1.394522	2.075767	−1.677646	H	−1.213808	1.385627	2.118595
H	2.843189	2.002671	−0.659788	H	−1.428264	−0.360257	1.962148
H	−0.050885	−1.839126	−1.736330	H	−2.774189	0.738533	1.583333

Table S17. Bioactivities of the compounds **1**–**6** against A549 (lung carcinoma).

**Species**	**0.5 μg/mL (Inhibition, %)**	**1 μg/mL (Inhibition, %)**	**10 μg/mL (Inhibition, %)**	**50 μg/mL (Inhibition, %)**	**100 μg/mL (Inhibition, %)**
**1**	−22.09	−13.85	−3.16	24.74	73.61
**2**	−47.21	−18.58	−5.46	3.69	47.33
**3**	18.81	22.12	27.70	58.60	84.34
**4**	13.89	21.59	23.86	29.28	18.24
**5**	−11.98	−0.51	0.03	3.83	17.37
**6**	17.37	16.80	23.35	48.41	65.42

Table S18. Bioactivities of the compounds **1**–**6** against HeLa (cervical cancer).

Species	0.5 µg/mL (Inhibition, %)	1 µg/mL (Inhibition, %)	10 µg/mL (Inhibition, %)	50 µg/mL (Inhibition, %)	100 µg/mL (Inhibition, %)
**1**	−6.64	−1.03	7.18	38.67	67.37
**2**	−10.47	−8.09	−0.06	19.02	43.31
**3**	21.46	21.94	29.22	58.07	76.25
**4**	18.05	17.39	31.90	36.19	40.69
**5**	−3.27	−5.84	5.43	31.00	43.38
**6**	21.46	21.55	29.61	43.56	53.07

Table S19. Bioactivities of the compounds **1**–**6** against QGY-7703 (hepatocarcinoma).

Species	0.5 µg/mL (Inhibition, %)	1 µg/mL (Inhibition, %)	10 µg/mL (Inhibition, %)	50 µg/mL (Inhibition, %)	100 µg/mL (Inhibition, %)
**1**	−23.35	−3.94	4.79	22.90	37.92
**2**	−10.69	−2.46	7.56	19.68	26.52
**3**	6.40	11.76	17.40	28.35	69.95
**4**	11.49	9.79	13.55	23.93	27.91
**5**	−26.17	−15.96	−6.68	7.83	17.81
**6**	24.98	19.04	19.48	35.58	44.94

Table S20. Bioactivities of the compounds **1**–**6** against MDA-MB-231 (human breast adenocarcinoma).

Species	0.5 µg/mL (Inhibition, %)	1 µg/mL (Inhibition, %)	10 µg/mL (Inhibition, %)	50 µg/mL (Inhibition, %)	100 µg/mL (Inhibition, %)
**1**	−70.26	−50.50	−49.31	−43.75	2.45
**2**	−65.04	−34.17	−36.22	−52.02	−52.15
**3**	−18.37	−14.94	−17.18	−25.25	41.97
**4**	−13.48	−4.36	−9.52	−6.08	−30.27
**5**	−49.56	−27.59	−32.00	−38.24	−25.36
**6**	−7.53	−7.39	0.75	3.19	−12.81

© 2014 by the authors; licensee MDPI, Basel, Switzerland. This article is an open access article distributed under the terms and conditions of the Creative Commons Attribution license (http://creativecommons.org/licenses/by/3.0/).
